# Supplementary figures and images for: The cuproptosis-related gene ITGB6 and LTBP1 may be associated with diabetic kidney disease progression and immune cell infiltration
Source: PeerJ. 2025 Nov 11;13:e20346. doi: 10.7717/peerj.20346 (PMC12617370; doi:10.7717/peerj.20346)

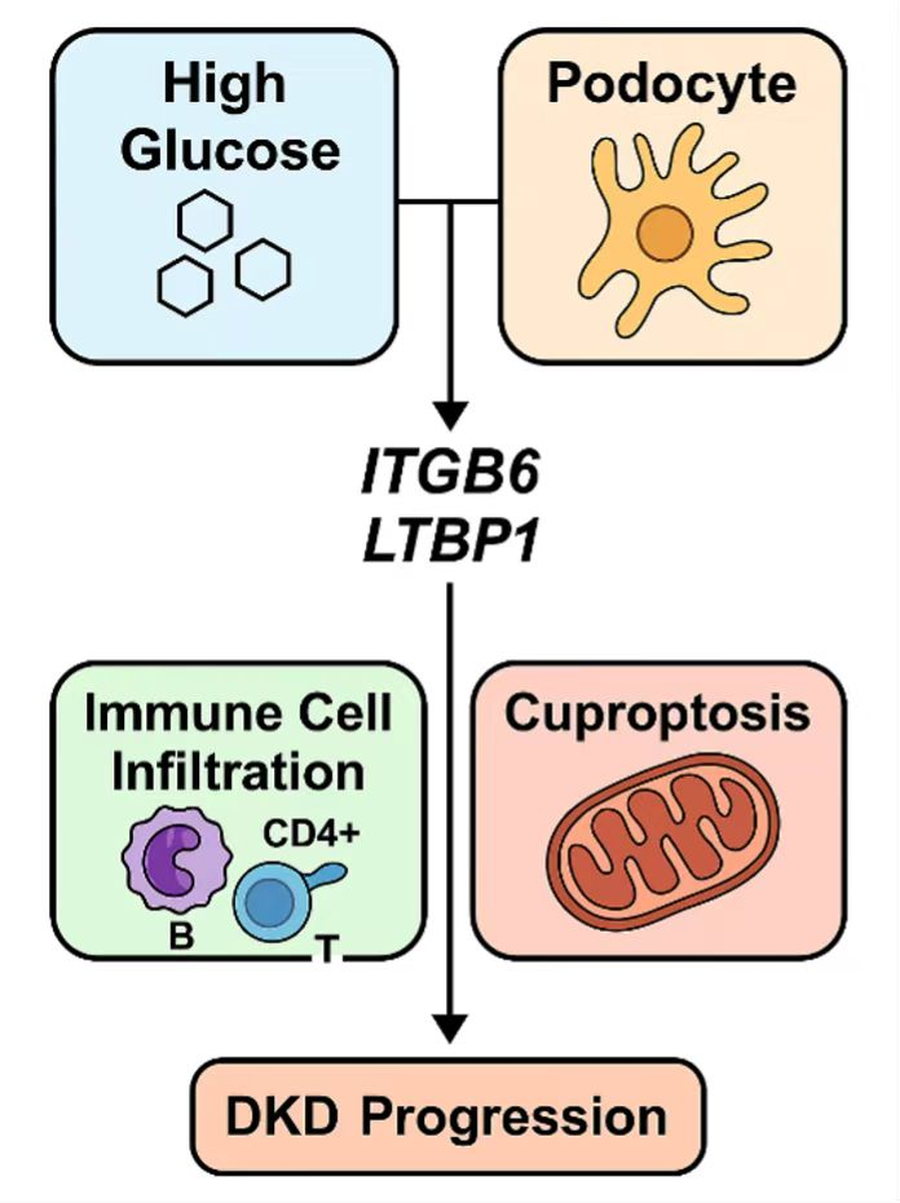

Supplement: Supplemental Information 1 — High glucose induces podocyte activation and upregulation of ITGB6 and LTBP1, which correlate with immune cell infiltration and cuproptosis, contributing to diabetic kidney disease (DKD) progression. [file peerj-13-20346-s001.png]

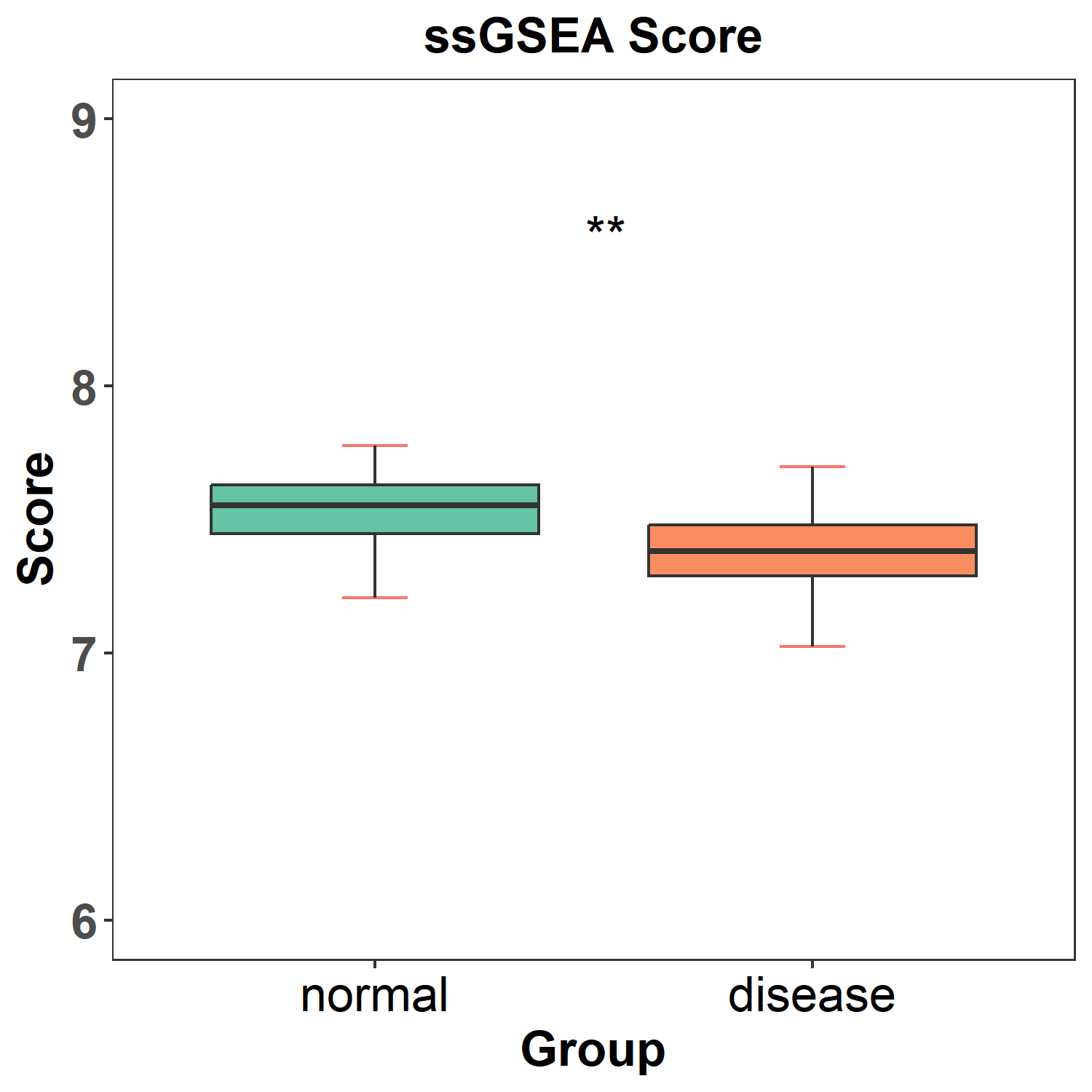

Supplement: Supplemental Information 3 [file peerj-13-20346-s003.zip › supplementary file/02_ssGSEA_score/01.ssGSEA_Score.png]

# ssGSEA Score

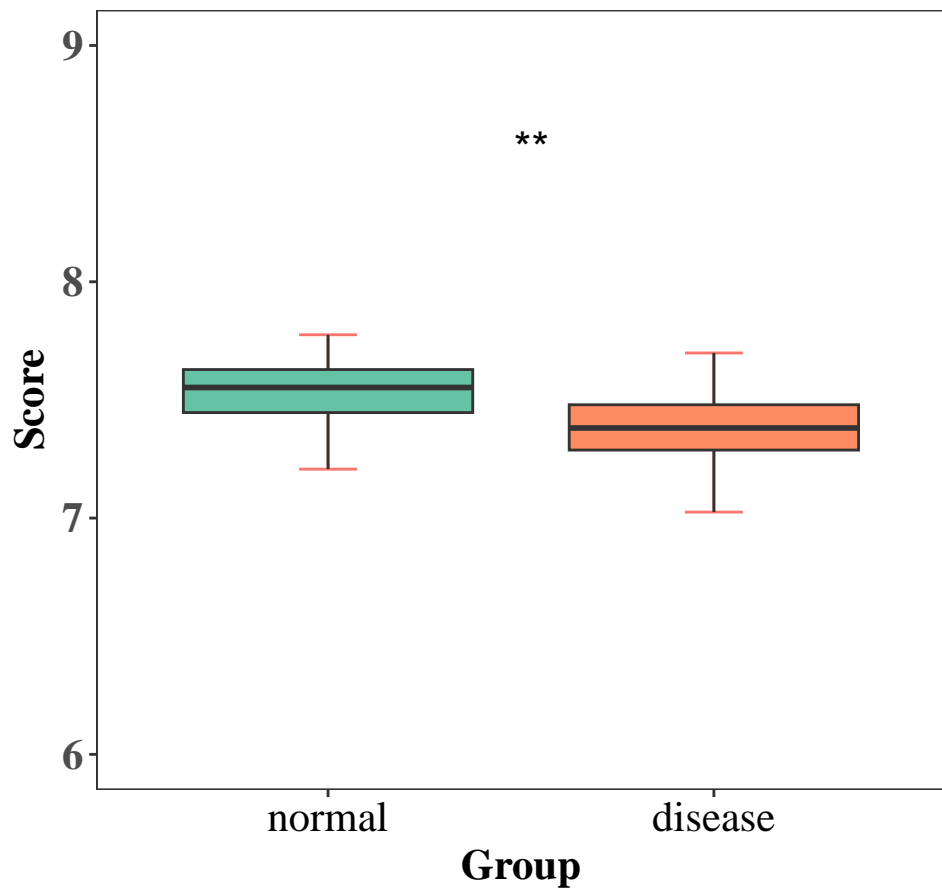

Supplement: Supplemental Information 3 [file peerj-13-20346-s003.zip › supplementary file/02_ssGSEA_score/01.ssGSEA_Score.pdf]

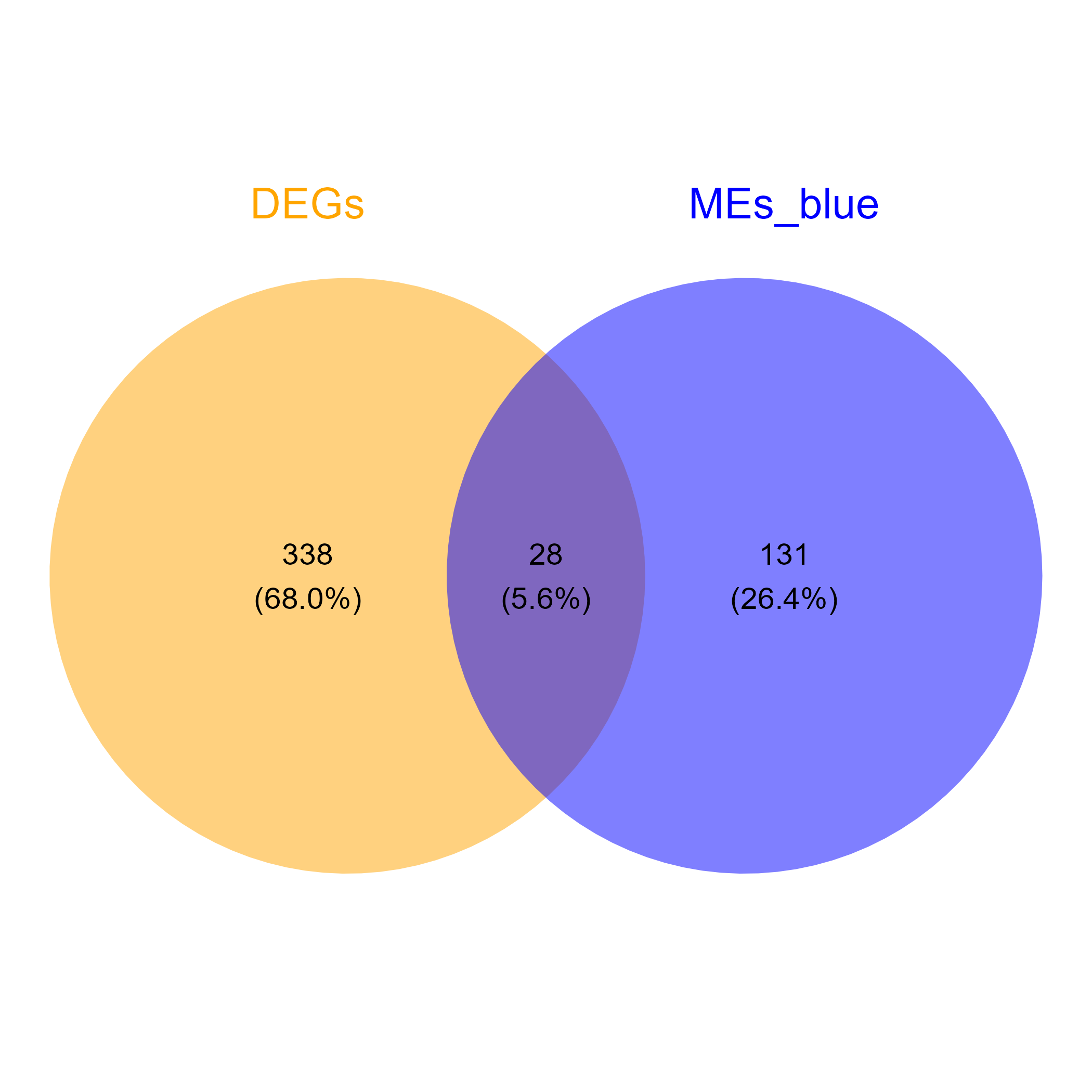

Supplement: Supplemental Information 3 [file peerj-13-20346-s003.zip › supplementary file/04_venn/01.Veen_blue.png]

DEGs

MEs\_blue

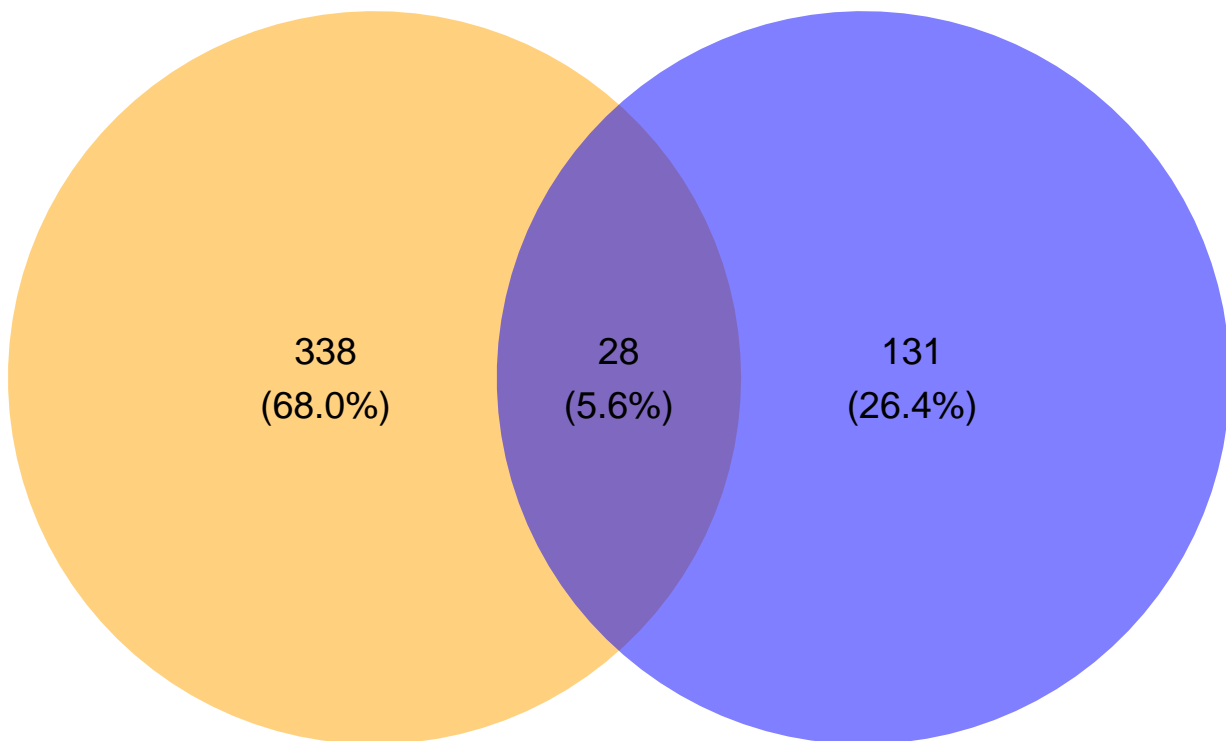

Supplement: Supplemental Information 3 [file peerj-13-20346-s003.zip › supplementary file/04_venn/01.Veen_blue.pdf]

DEGs

MEs\_brown

242  
(38.3%)

124  
(19.6%)

266  
(42.1%)

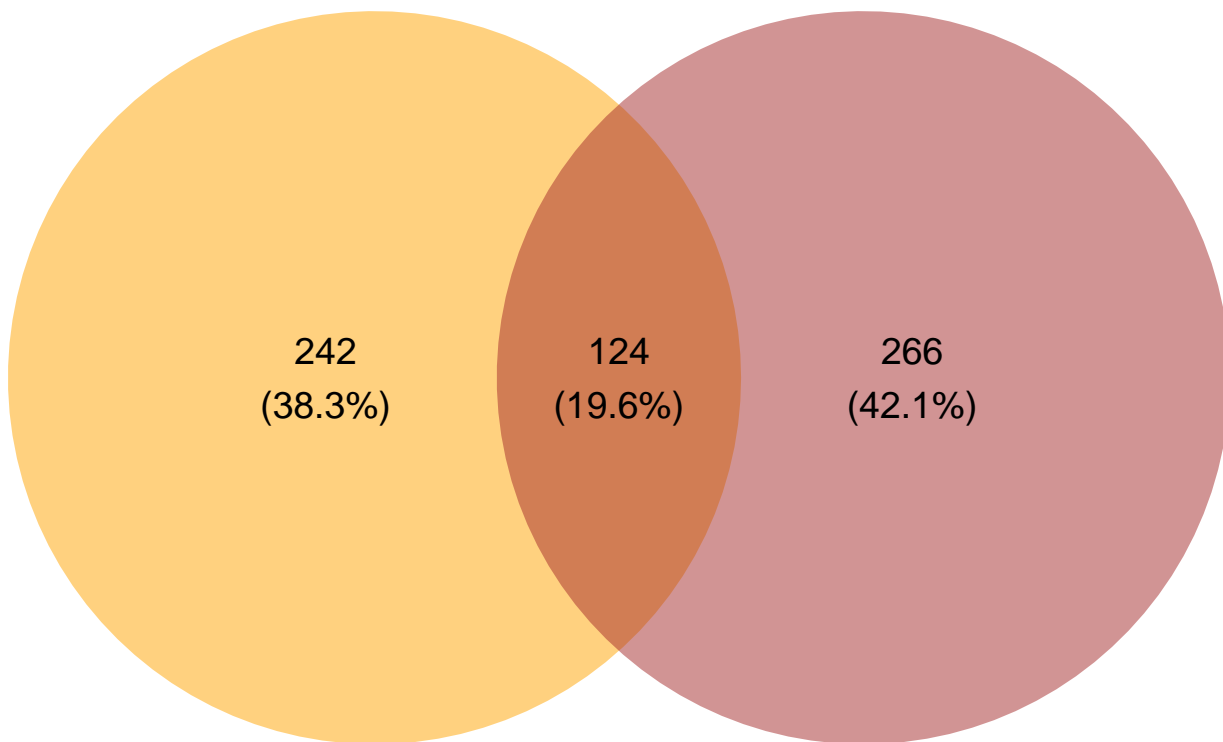

Supplement: Supplemental Information 3 [file peerj-13-20346-s003.zip › supplementary file/04_venn/01.Veen_brown.pdf]

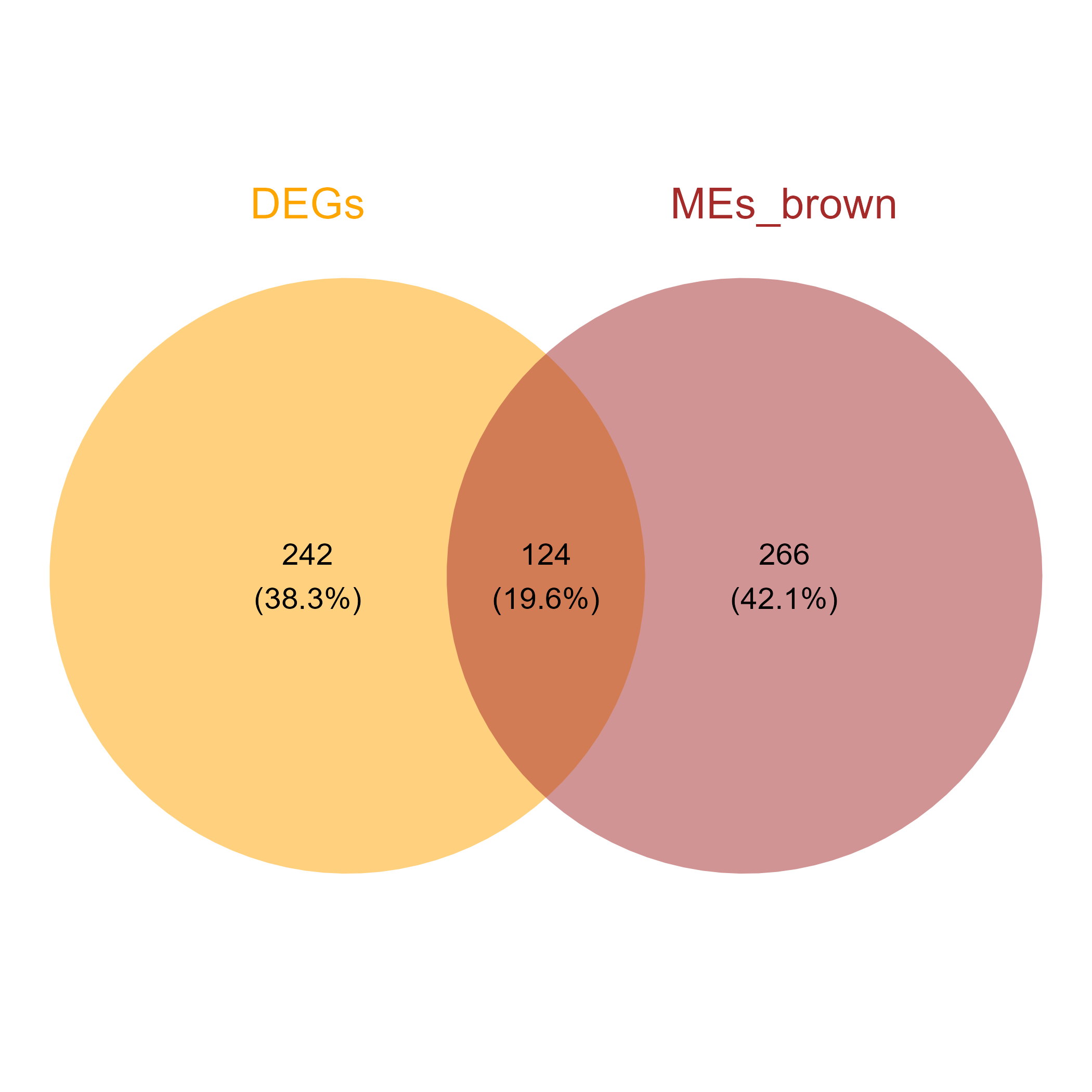

Supplement: Supplemental Information 3 [file peerj-13-20346-s003.zip › supplementary file/04_venn/01.Veen_brown.png]

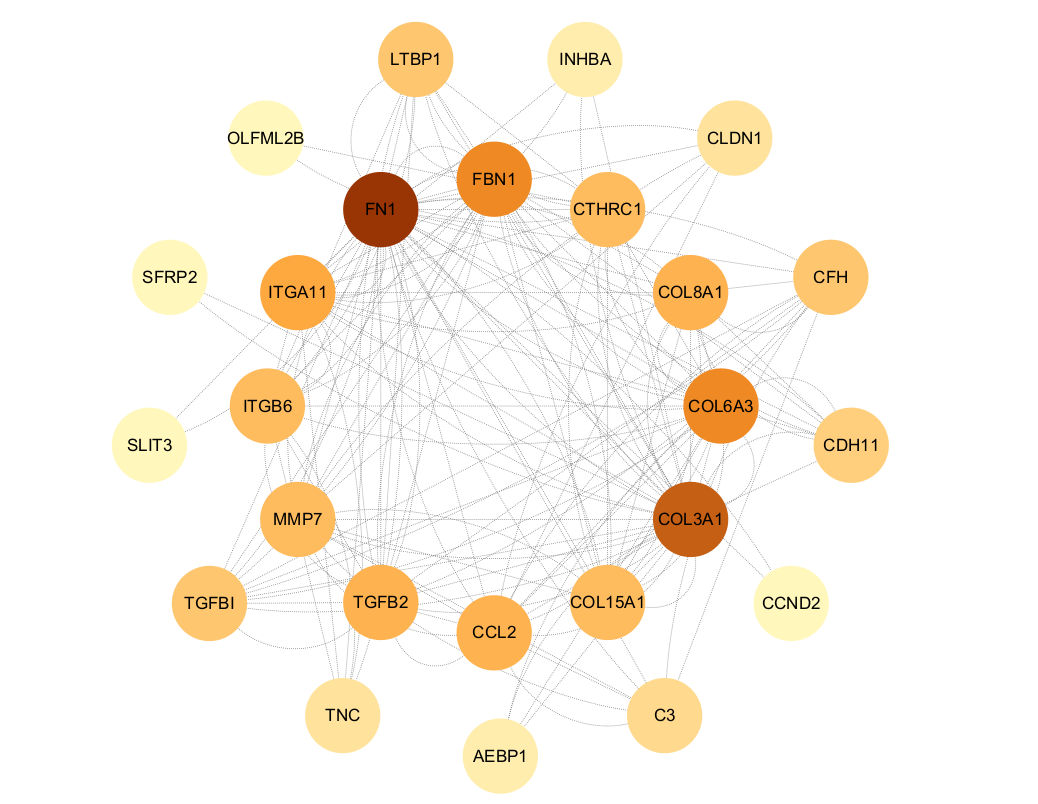

Supplement: Supplemental Information 3 [file peerj-13-20346-s003.zip › supplementary file/10_ppi/string_interactions.tsv.png]

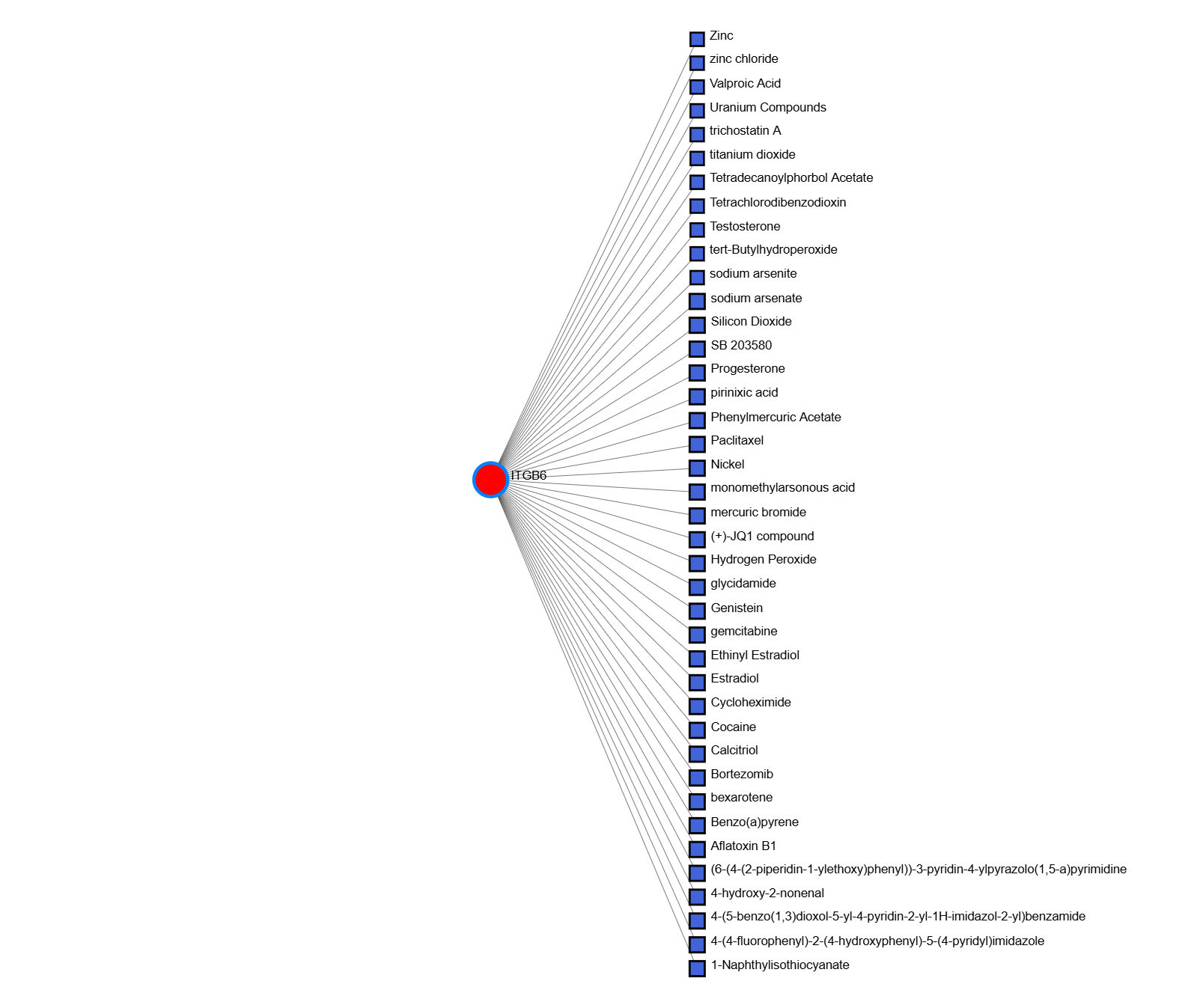

Supplement: Supplemental Information 3 [file peerj-13-20346-s003.zip › supplementary file/12_drug_prediction/ITGB6_chemicals.png]

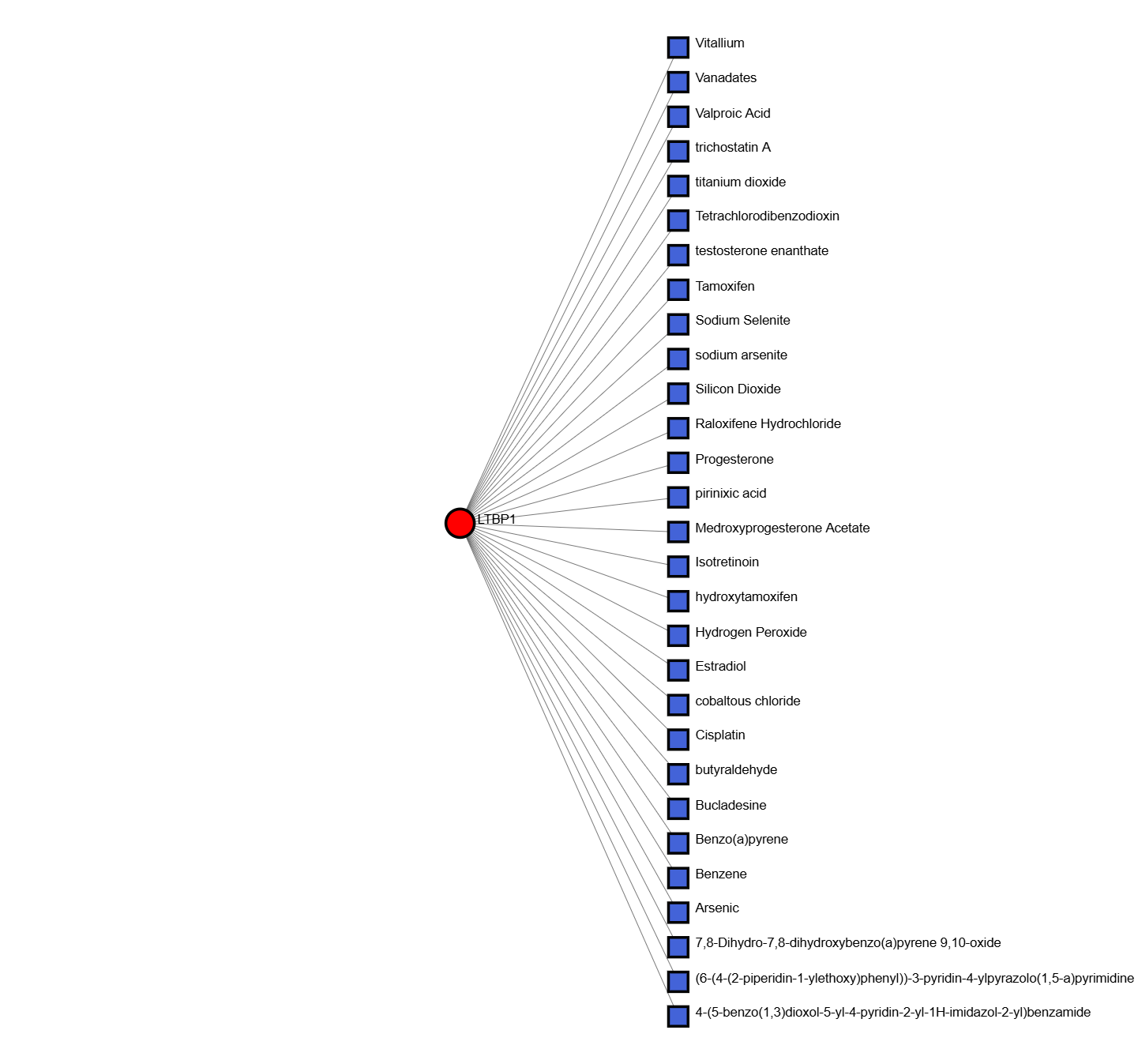

Supplement: Supplemental Information 3 [file peerj-13-20346-s003.zip › supplementary file/12_drug_prediction/LTBP1_chemicals.png]

lasso

xgboost

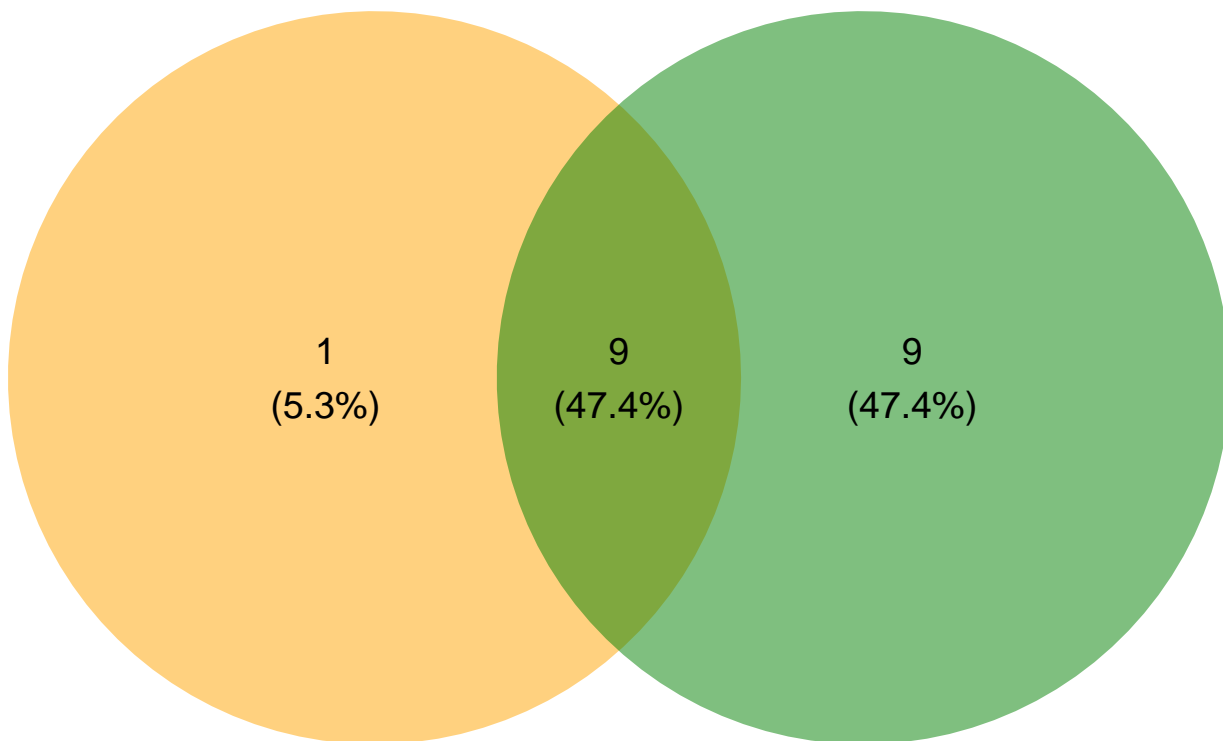

Supplement: Supplemental Information 3 [file peerj-13-20346-s003.zip › supplementary file/05_Machine_learning/blue_hub2.pdf]

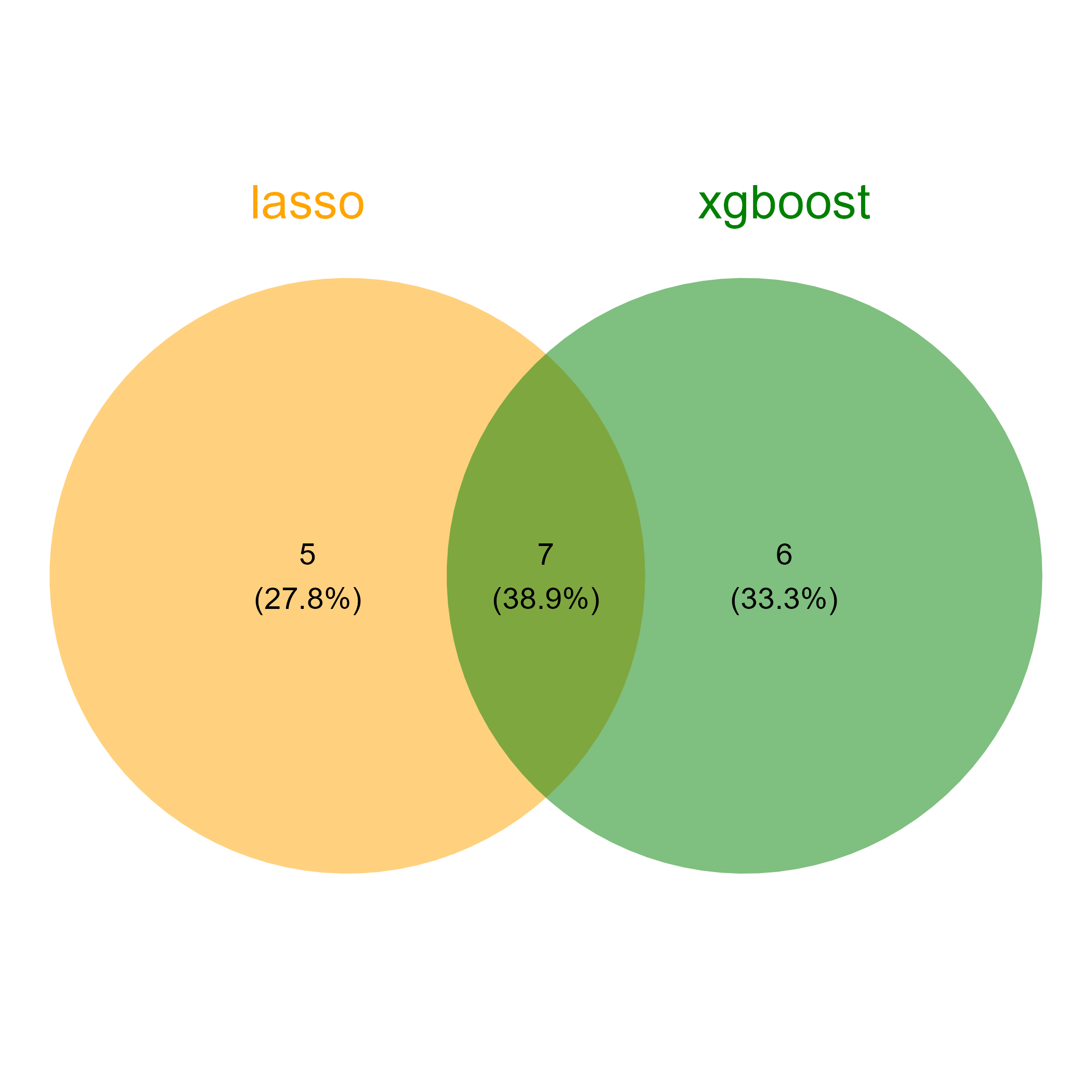

Supplement: Supplemental Information 3 [file peerj-13-20346-s003.zip › supplementary file/05_Machine_learning/brown_hub2.png]

lasso

xgboost

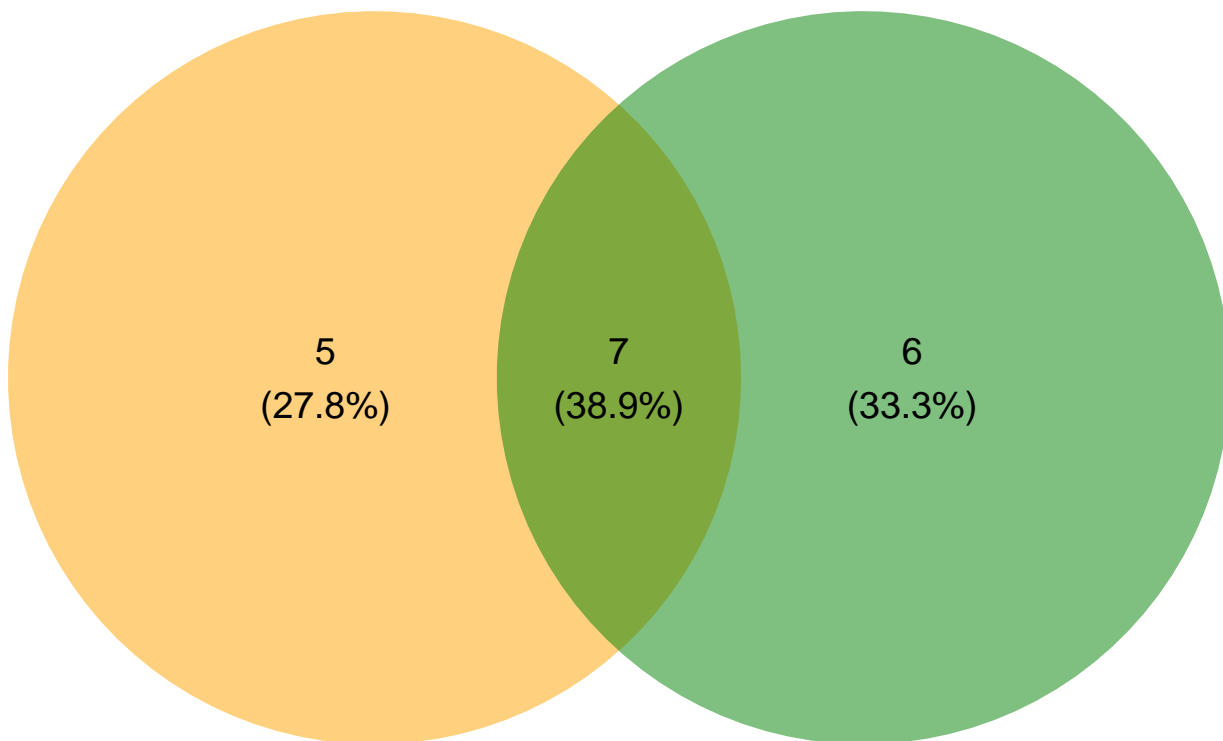

Supplement: Supplemental Information 3 [file peerj-13-20346-s003.zip › supplementary file/05_Machine_learning/brown_hub2.pdf]

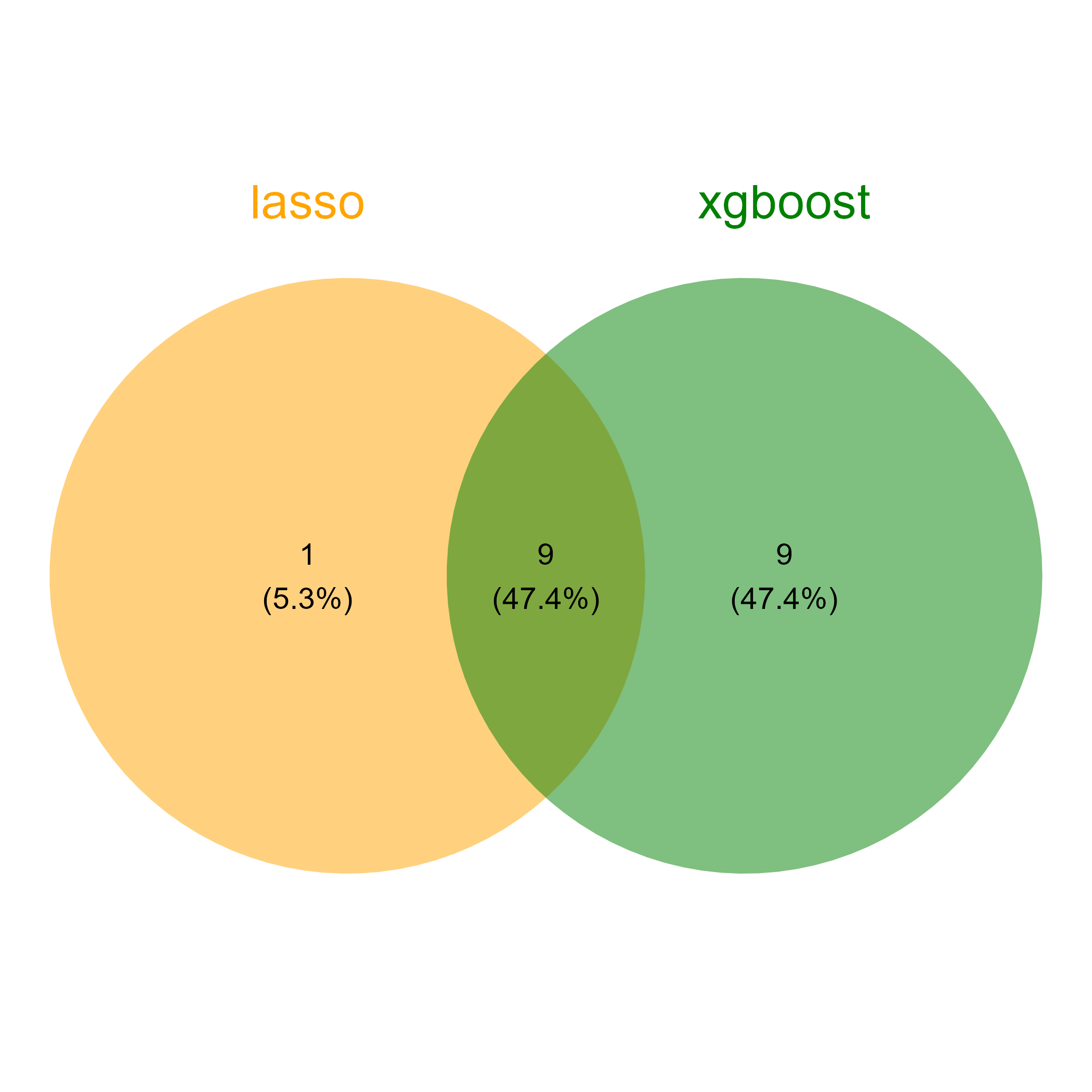

Supplement: Supplemental Information 3 [file peerj-13-20346-s003.zip › supplementary file/05_Machine_learning/blue_hub2.png]

Distribution as heatmap

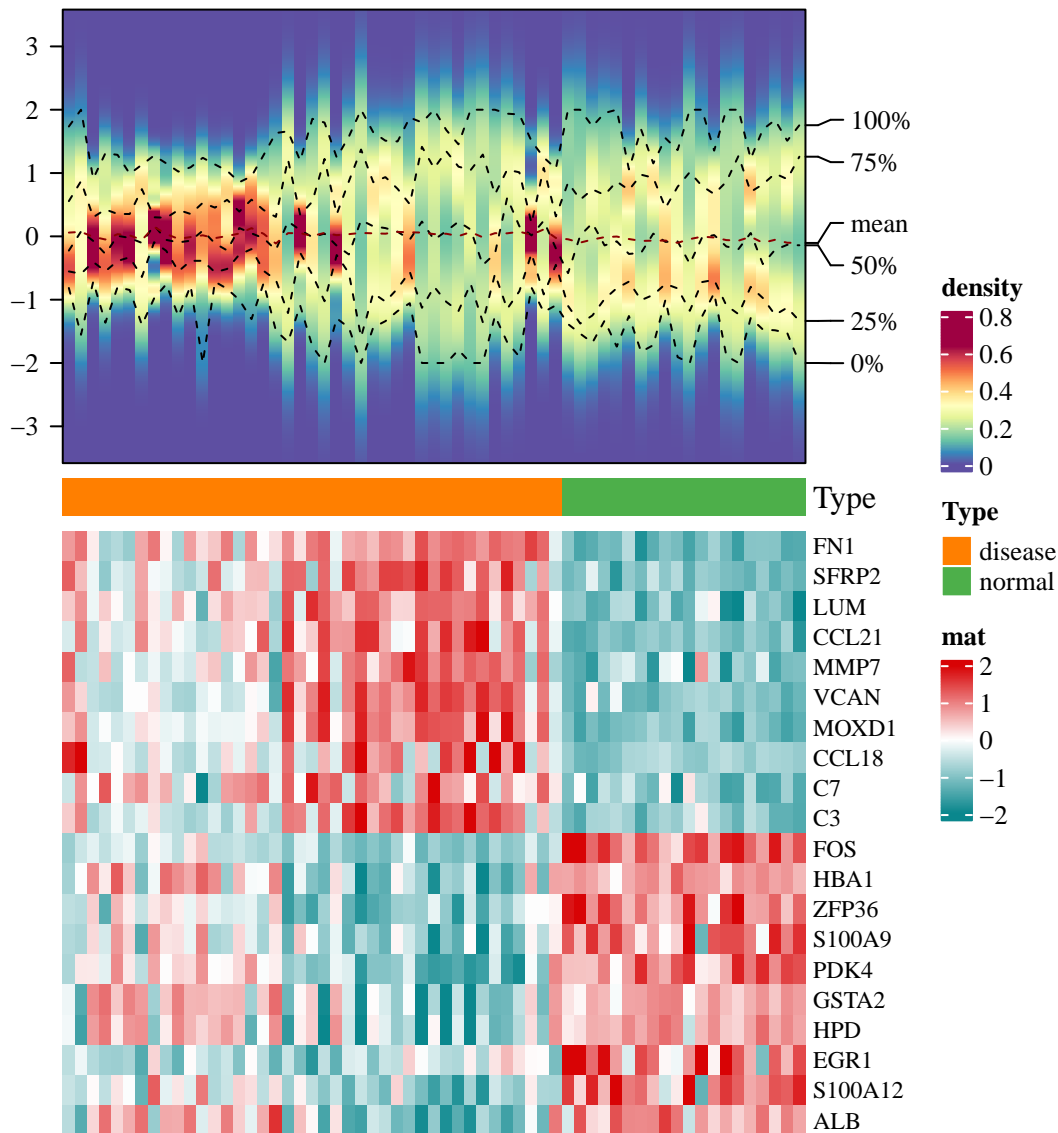

Supplement: Supplemental Information 3 [file peerj-13-20346-s003.zip › supplementary file/01_DEGs/05.GSE96804_heat.pdf]

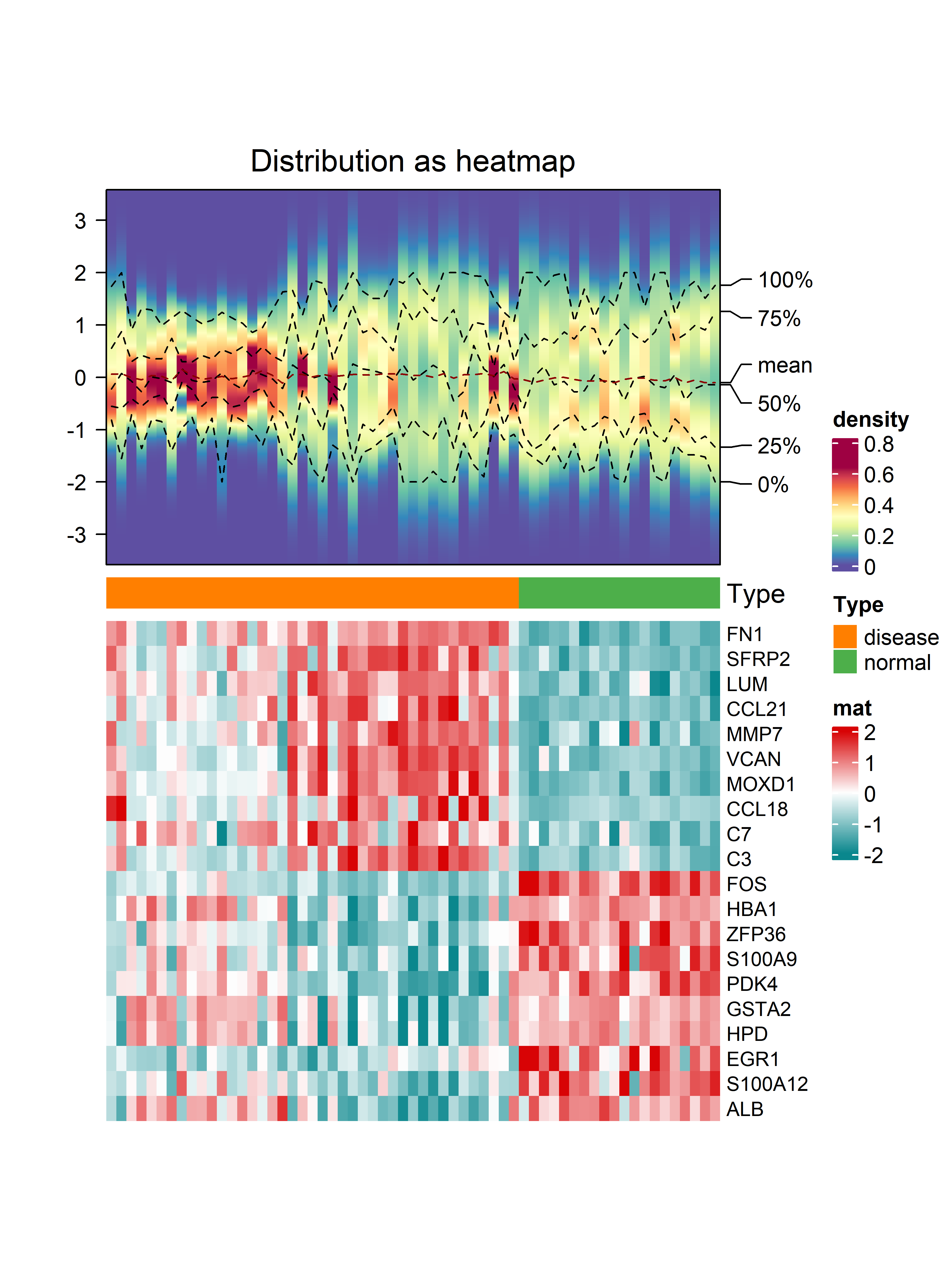

Supplement: Supplemental Information 3 [file peerj-13-20346-s003.zip › supplementary file/01_DEGs/05.GSE96804_heat.png]

Distribution as heatmap

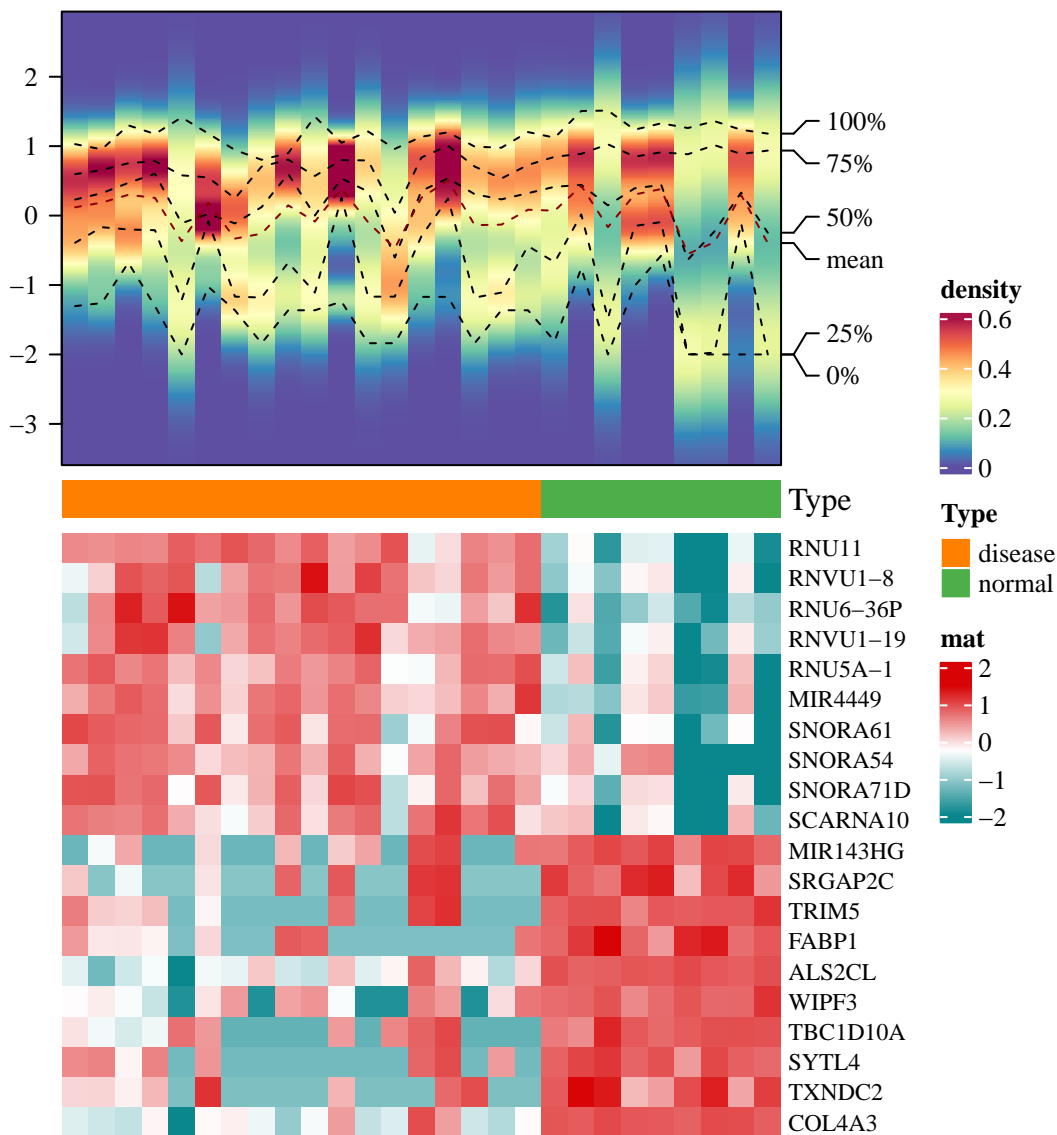

Supplement: Supplemental Information 3 [file peerj-13-20346-s003.zip › supplementary file/01_DEGs/05.GSE162830_heat.pdf]

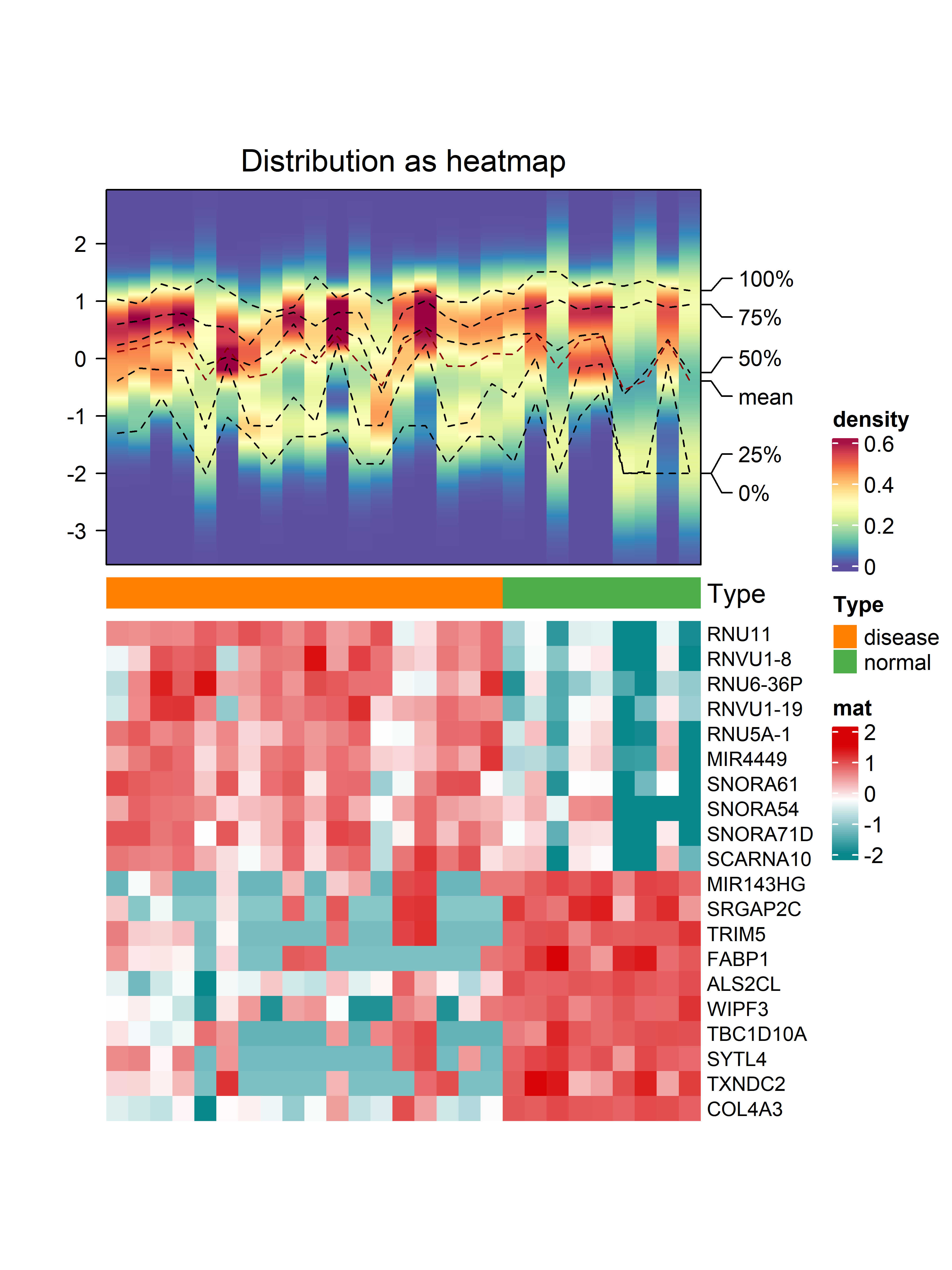

Supplement: Supplemental Information 3 [file peerj-13-20346-s003.zip › supplementary file/01_DEGs/05.GSE162830_heat.png]

# Diabetic Nephropathy vs Normal

*padj: 0.05; logFC: 1.00; Up: 107; Down: 259; Total: 366*

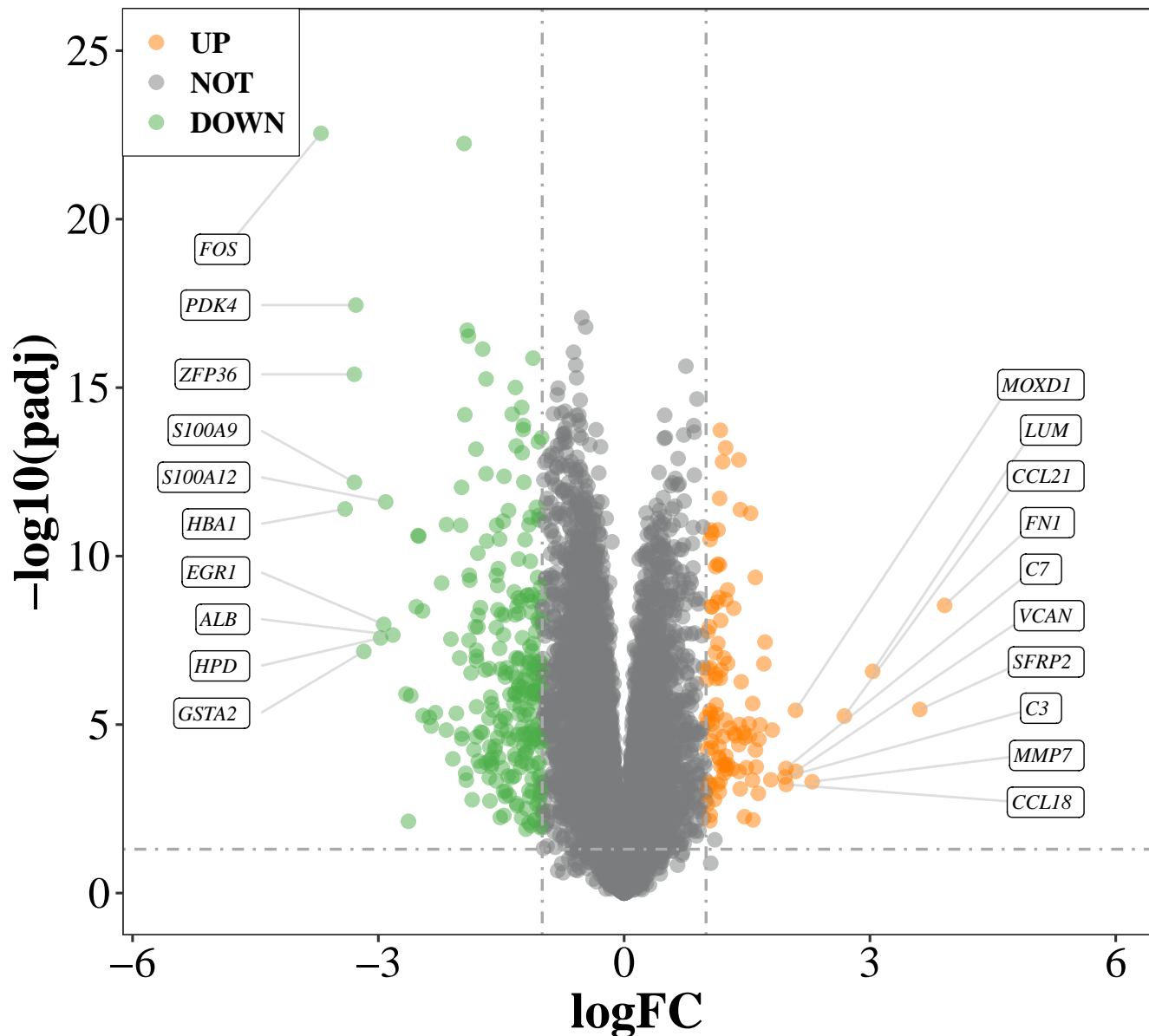

Supplement: Supplemental Information 3 [file peerj-13-20346-s003.zip › supplementary file/01_DEGs/04.GSE96804_volcano.pdf]

# Diabetic Nephropathy vs Normal

*Pvalue: 0.05; logFC: 2.00; Up: 229; Down: 2854; Total: 3083*

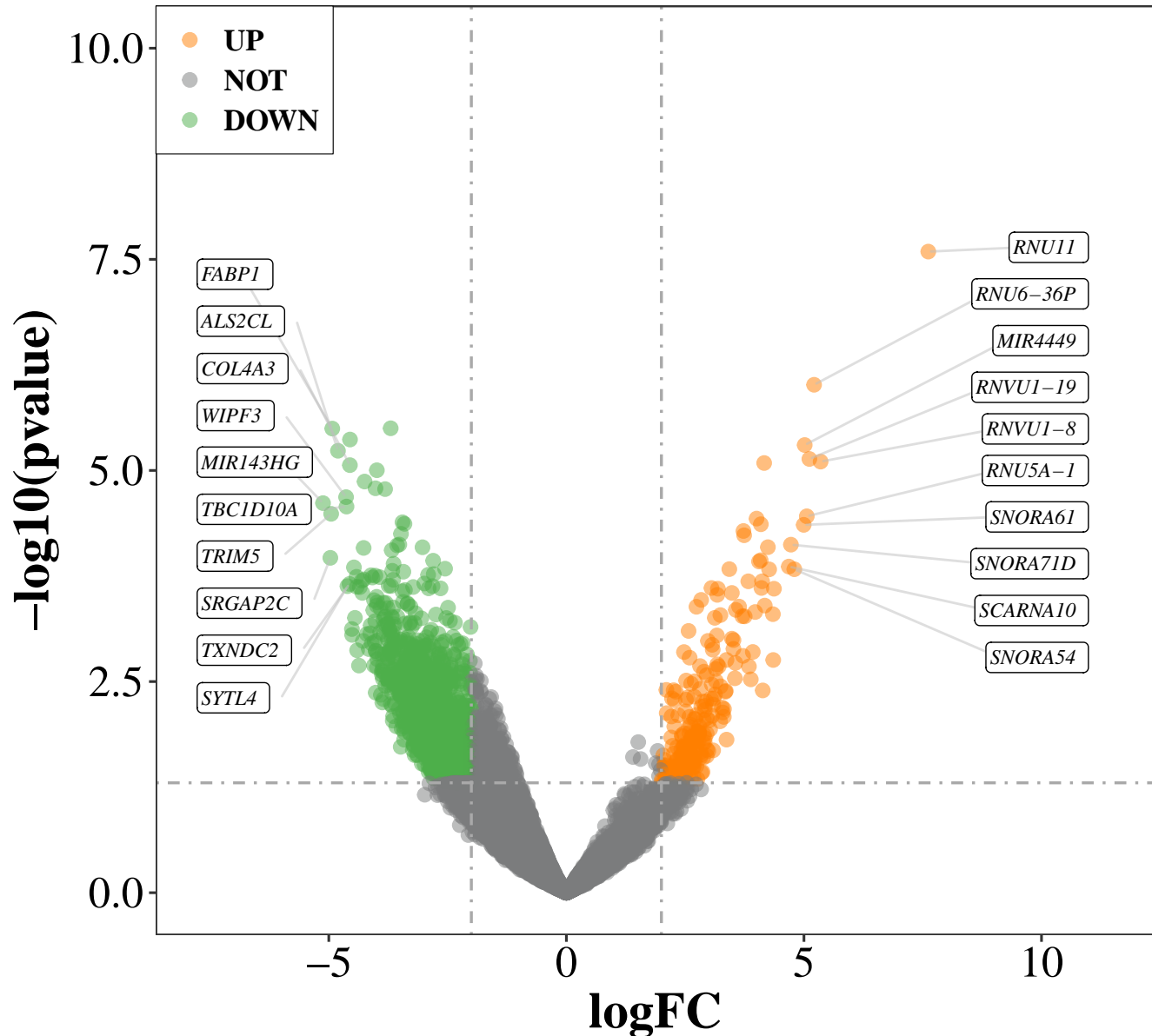

Supplement: Supplemental Information 3 [file peerj-13-20346-s003.zip › supplementary file/01_DEGs/04.GSE162830_volcano.pdf]

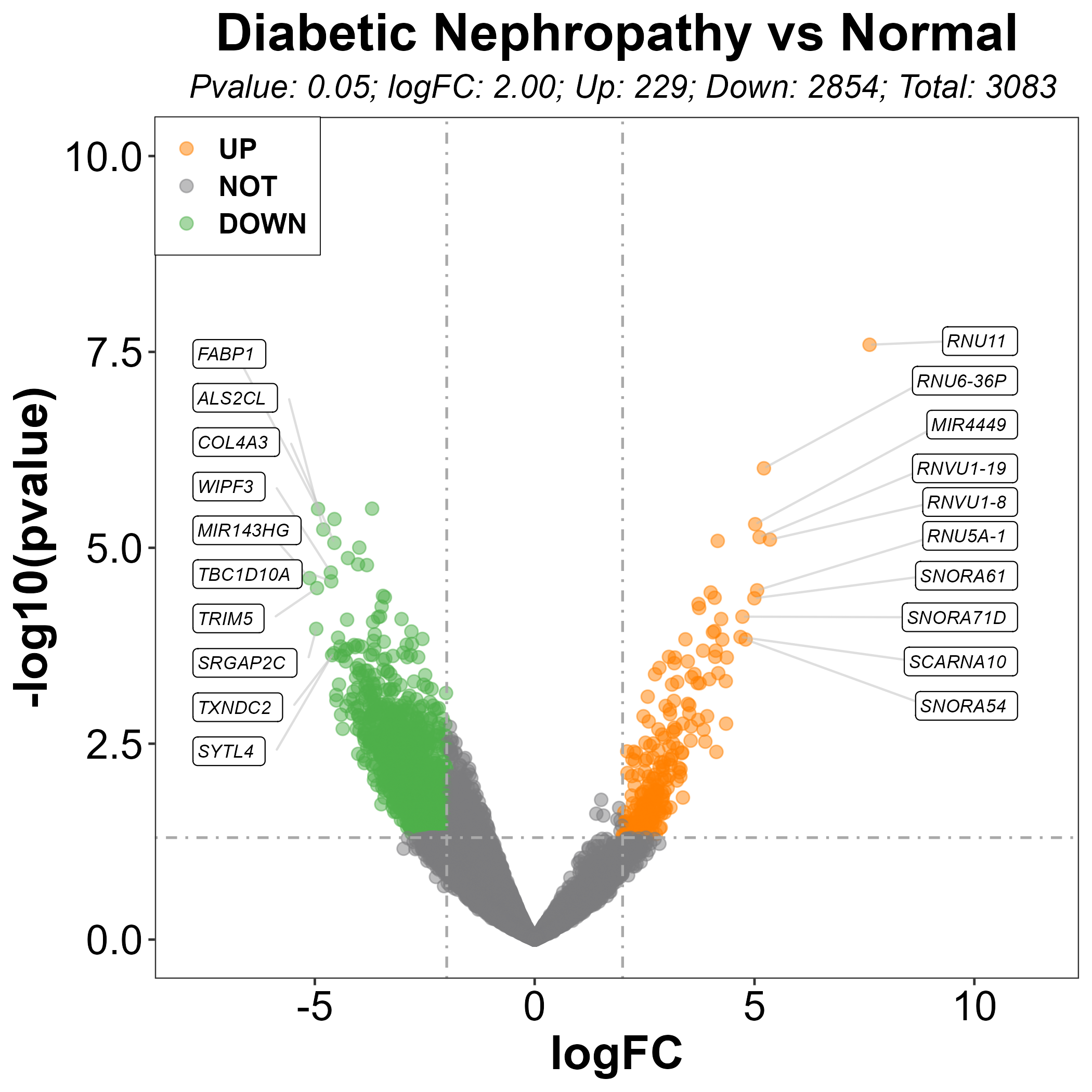

Supplement: Supplemental Information 3 [file peerj-13-20346-s003.zip › supplementary file/01_DEGs/04.GSE162830_volcano.png]

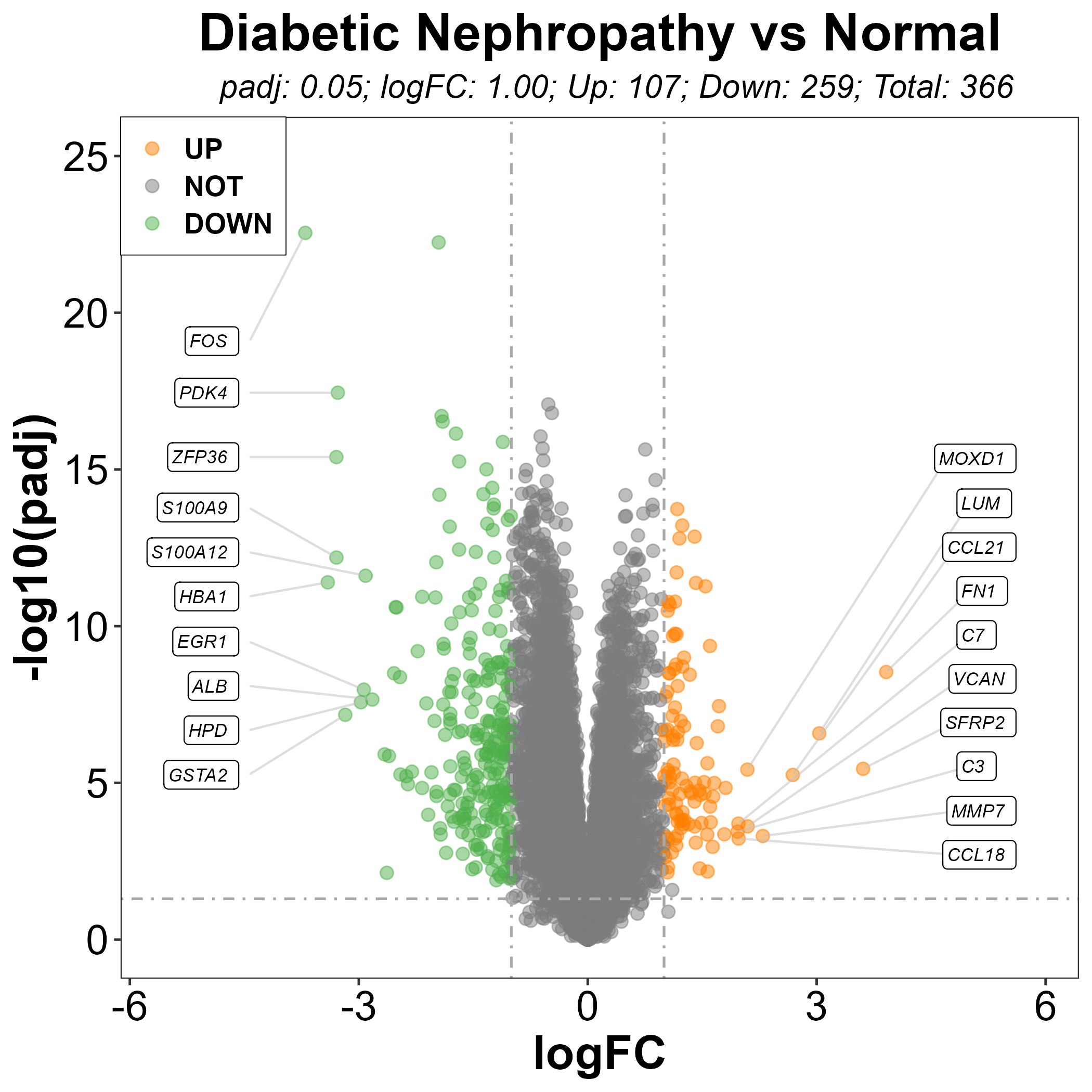

Supplement: Supplemental Information 3 [file peerj-13-20346-s003.zip › supplementary file/01_DEGs/04.GSE96804_volcano.png]

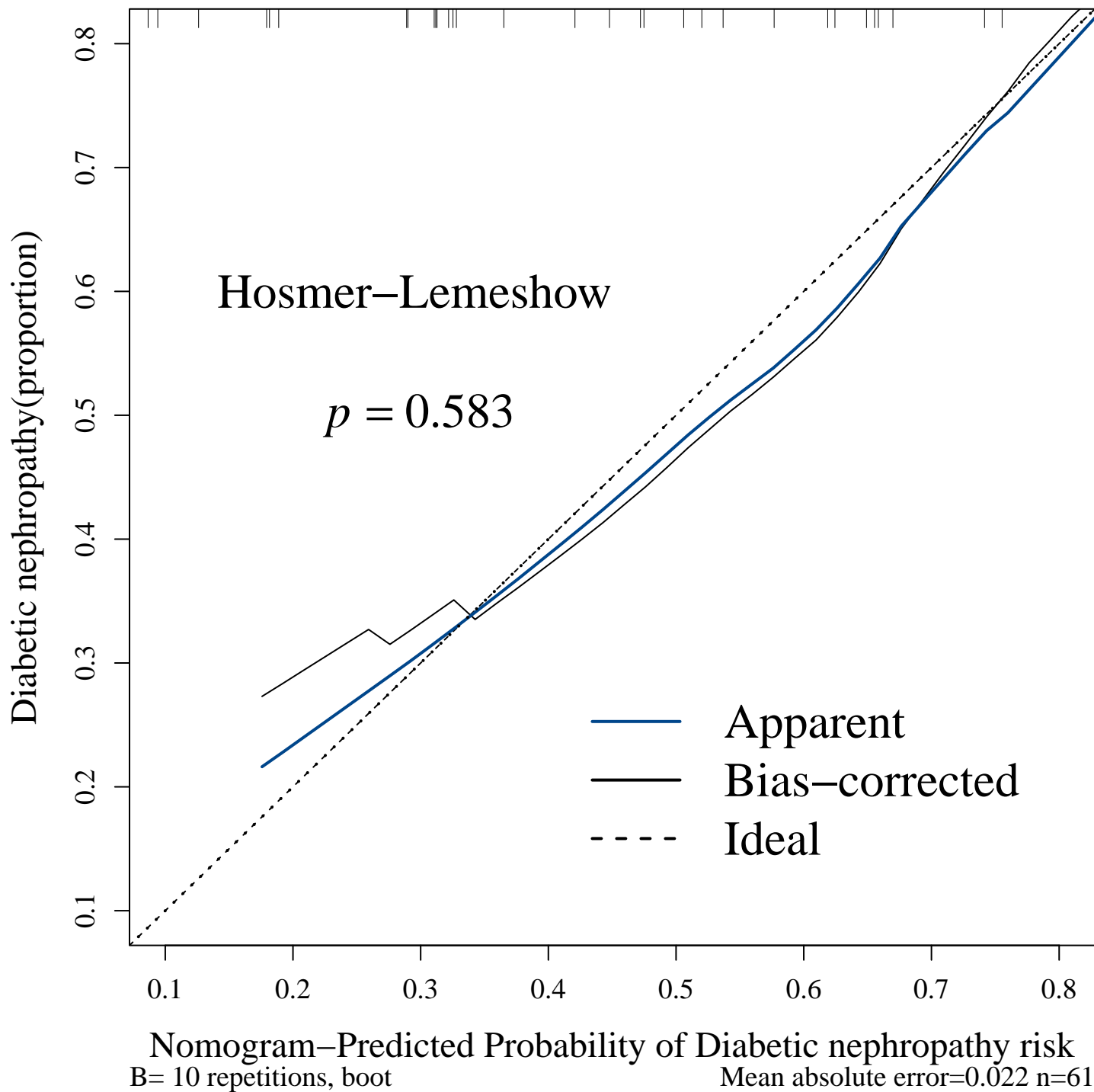

Supplement: Supplemental Information 3 [file peerj-13-20346-s003.zip › supplementary file/08_nomogram/03.calibrate.pdf]

# ***nomogram***

*Points*

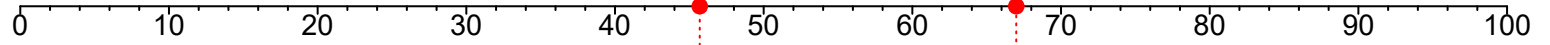

ITGB6

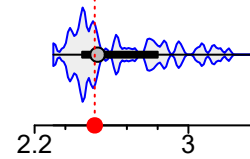

LTBP1

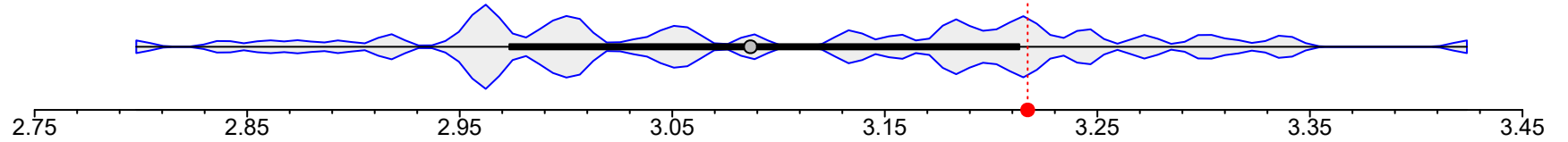

***Total points***

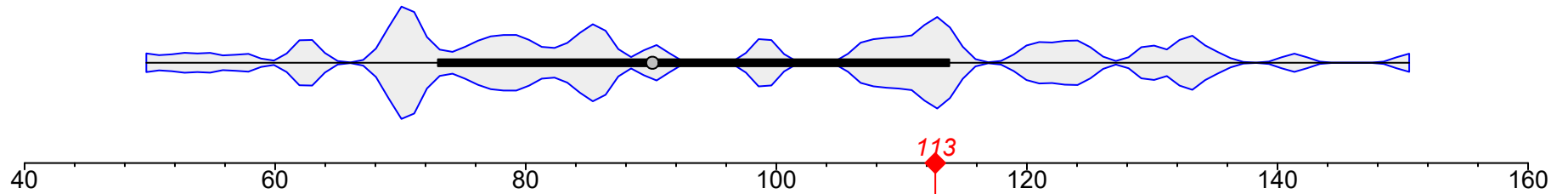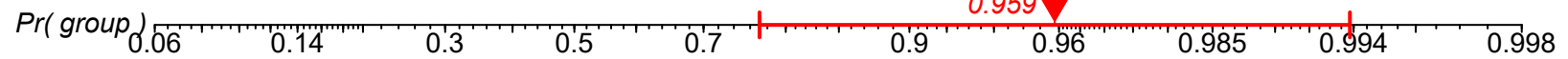

Supplement: Supplemental Information 3 [file peerj-13-20346-s003.zip › supplementary file/08_nomogram/01_nomogram.pdf]

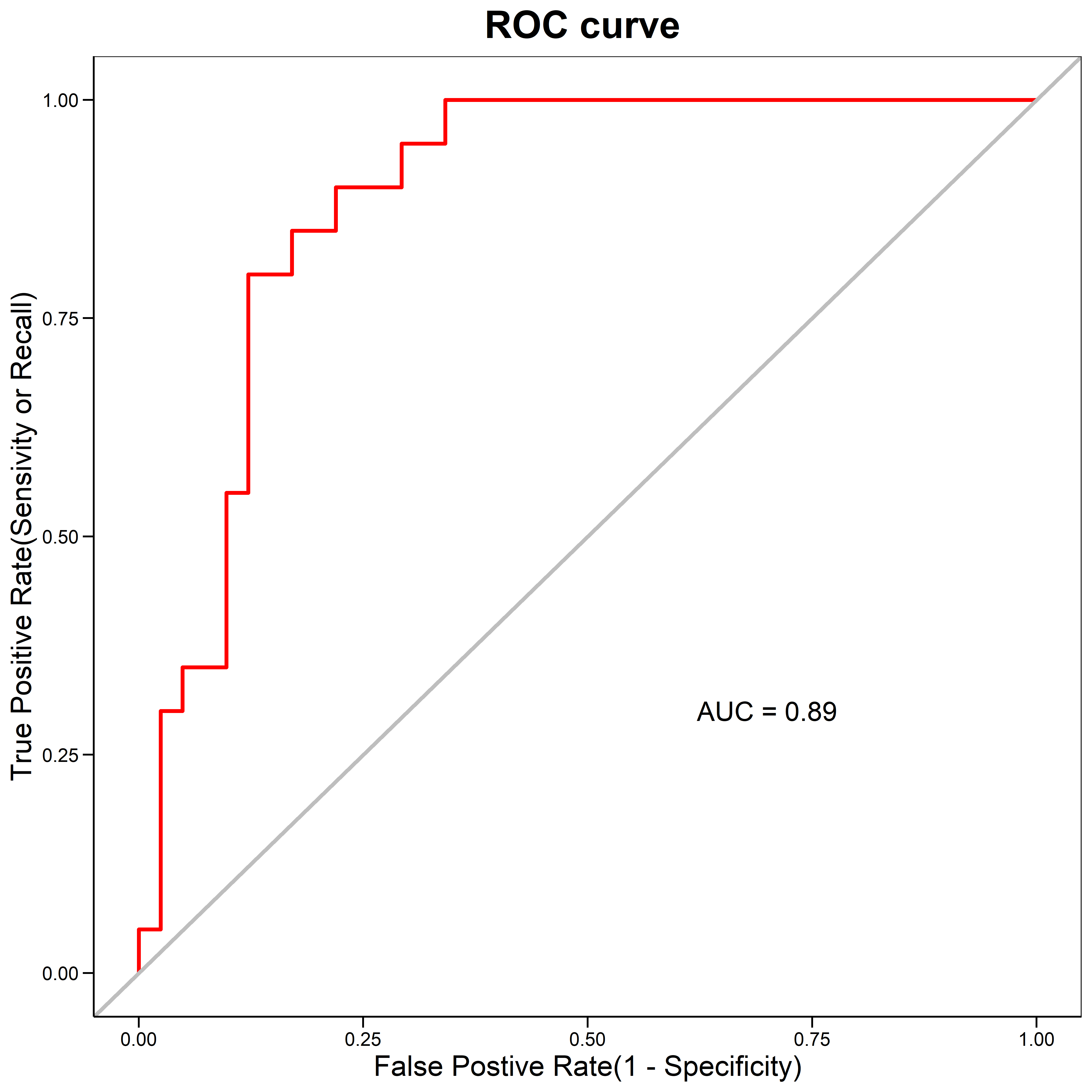

Supplement: Supplemental Information 3 [file peerj-13-20346-s003.zip › supplementary file/08_nomogram/03.calibrate.png]

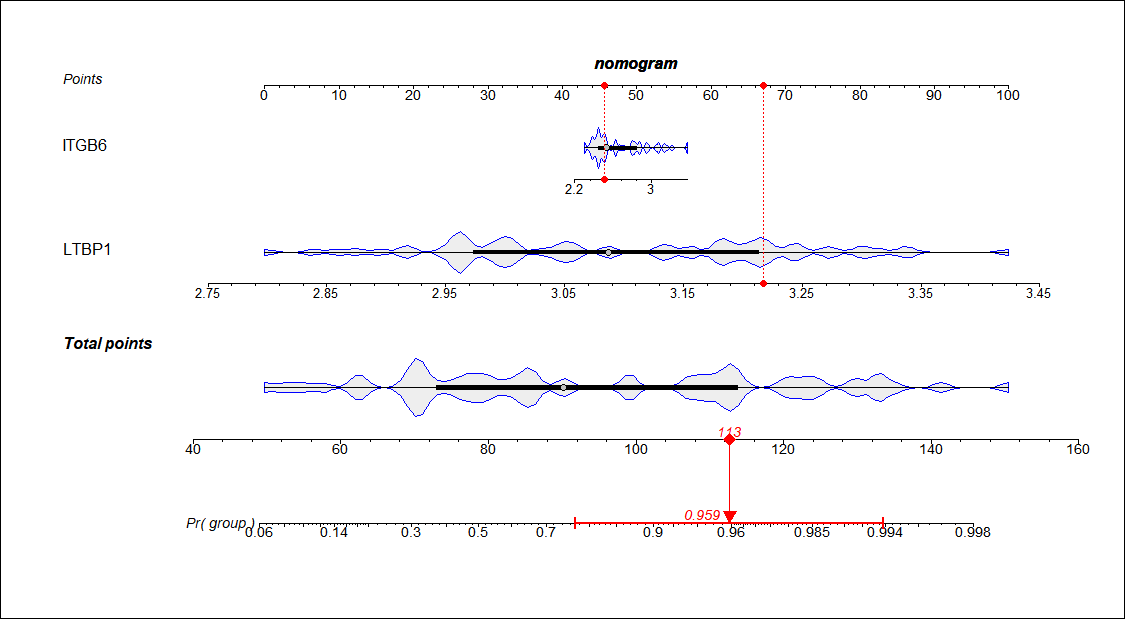

Supplement: Supplemental Information 3 [file peerj-13-20346-s003.zip › supplementary file/08_nomogram/01_nomogram.png]

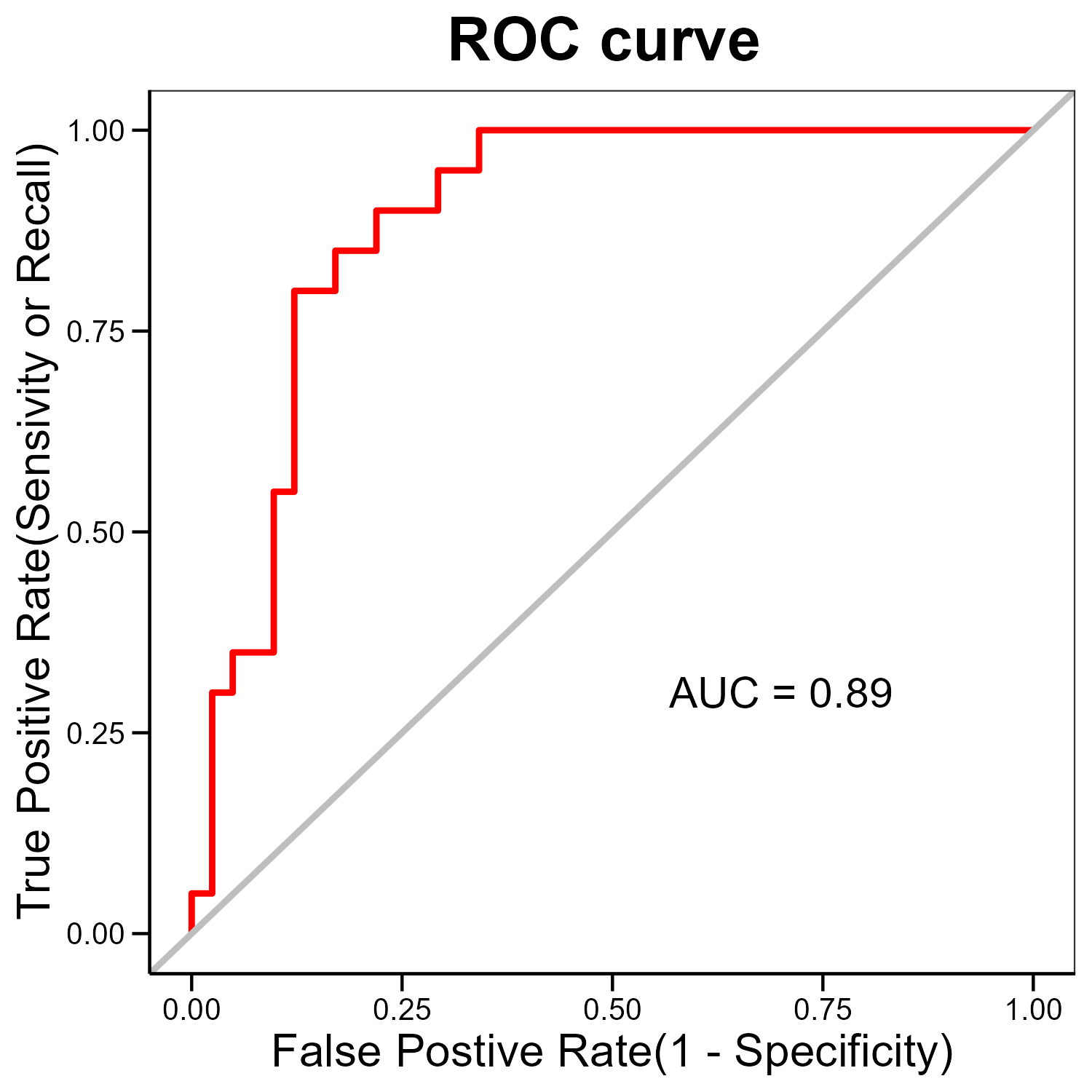

Supplement: Supplemental Information 3 [file peerj-13-20346-s003.zip › supplementary file/08_nomogram/04.model_ROC.png]

# ROC curve

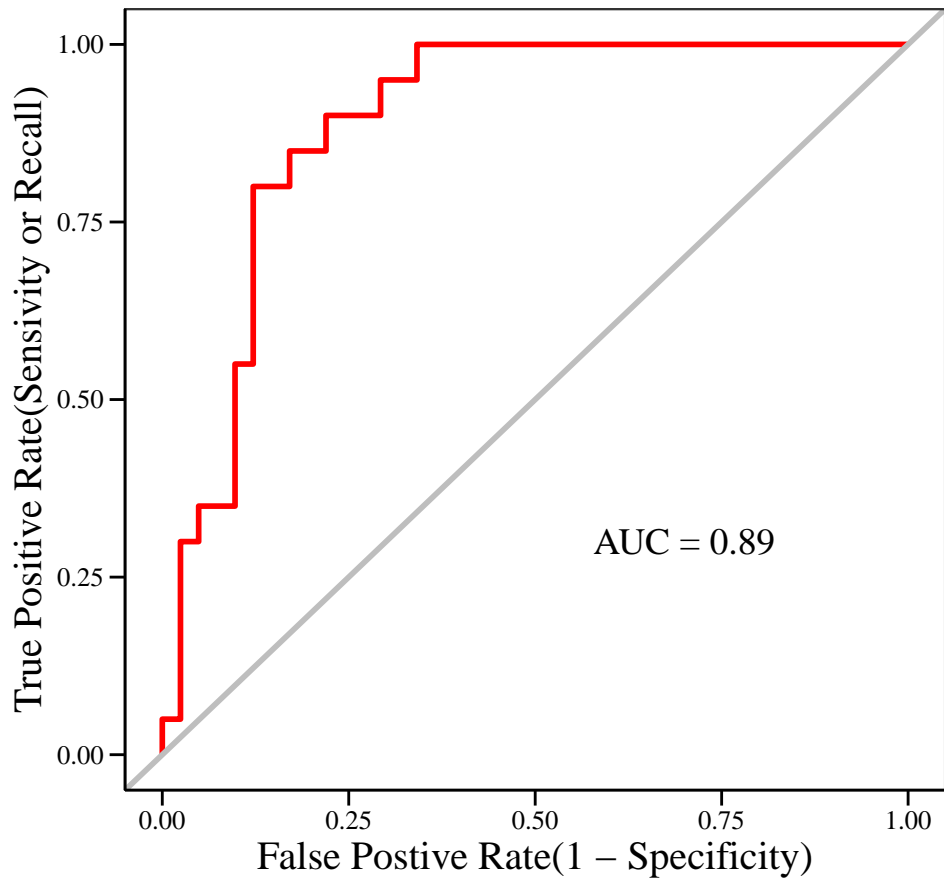

Supplement: Supplemental Information 3 [file peerj-13-20346-s003.zip › supplementary file/08_nomogram/04.model_ROC.pdf]

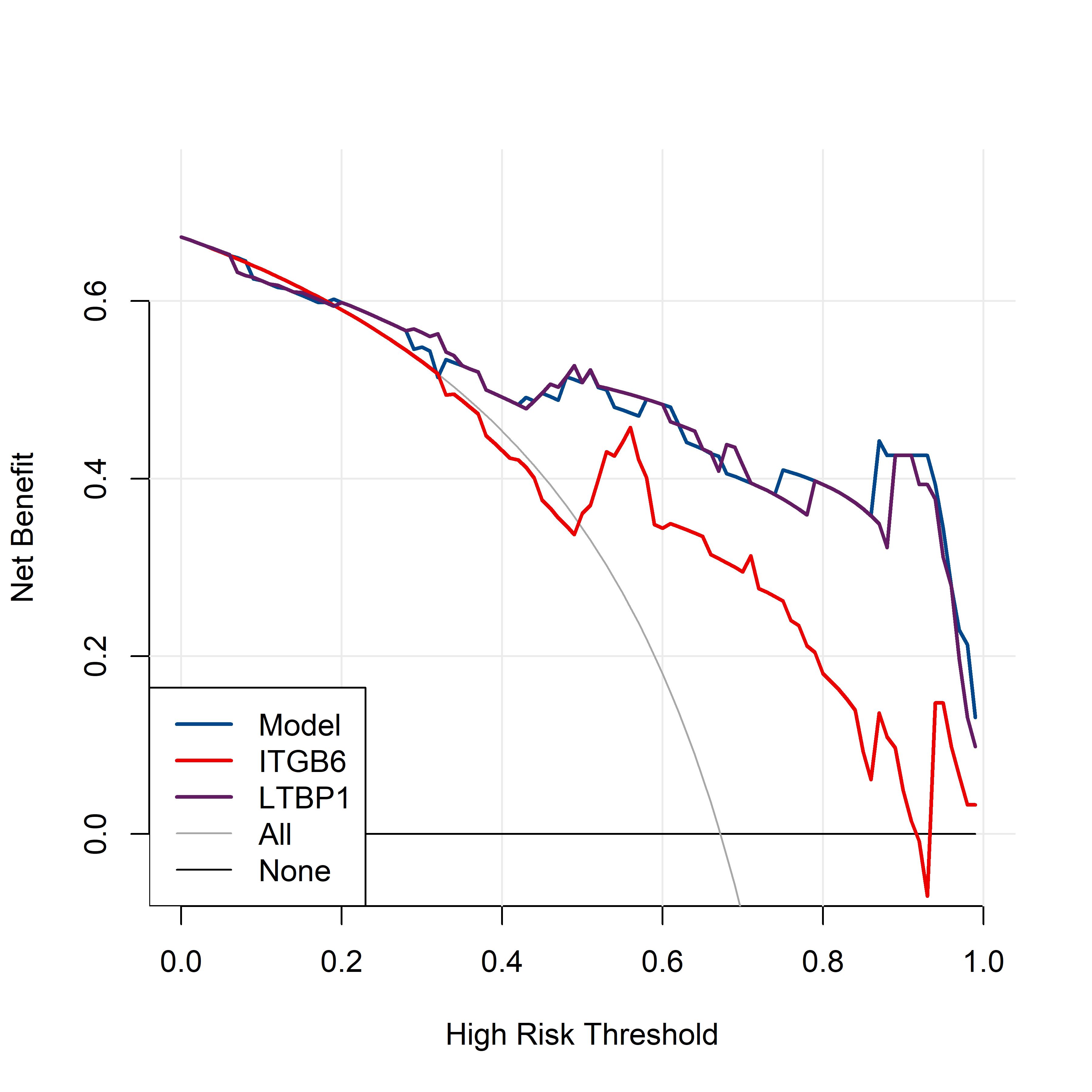

Supplement: Supplemental Information 3 [file peerj-13-20346-s003.zip › supplementary file/08_nomogram/05.Model_DCA.png]

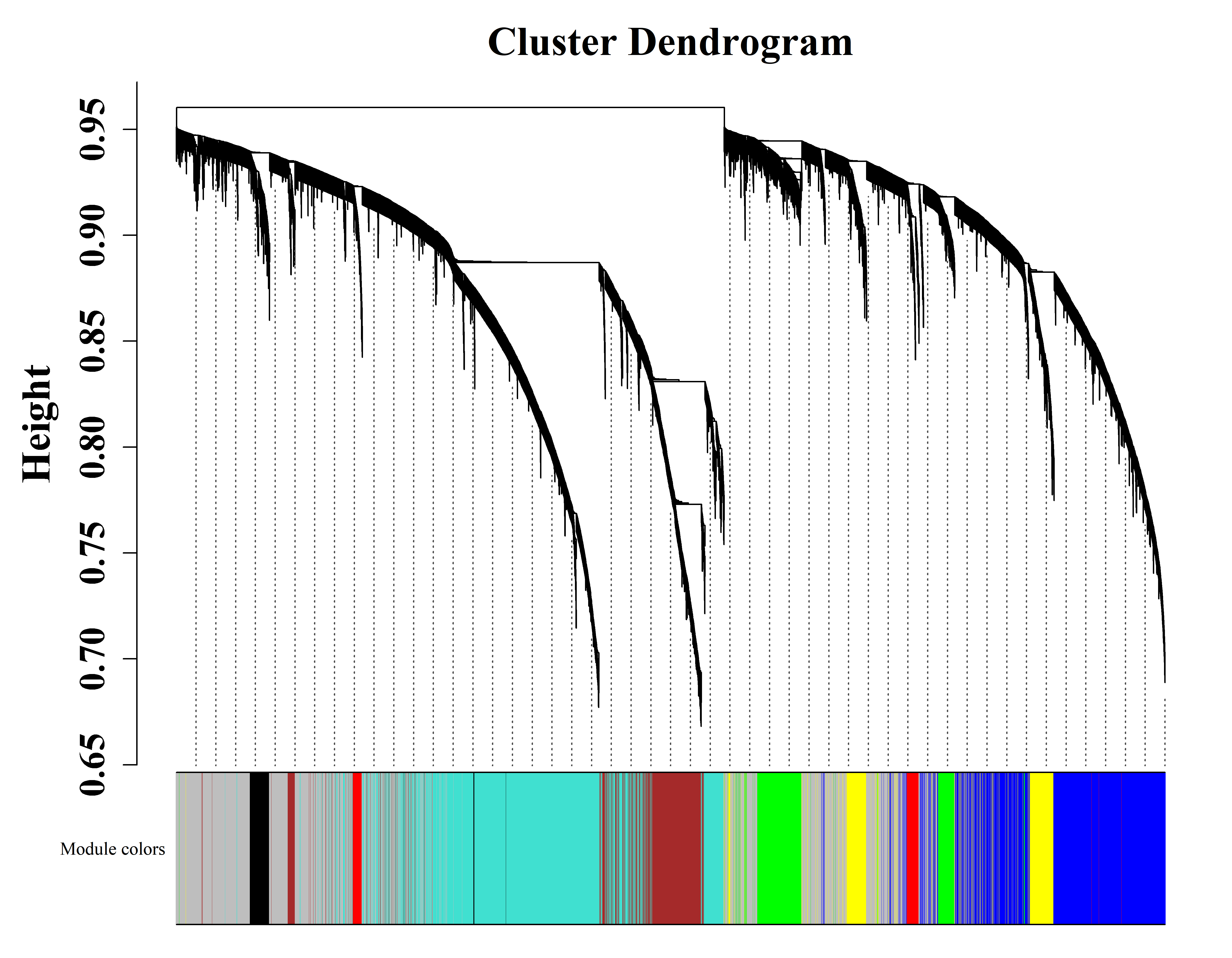

Supplement: Supplemental Information 3 [file peerj-13-20346-s003.zip › supplementary file/03_WGCNA/04.wgcna.dendroColors.png]

# Cluster Dendrogram

Height

0.65 0.70 0.75 0.80 0.85 0.90 0.95

Module colors

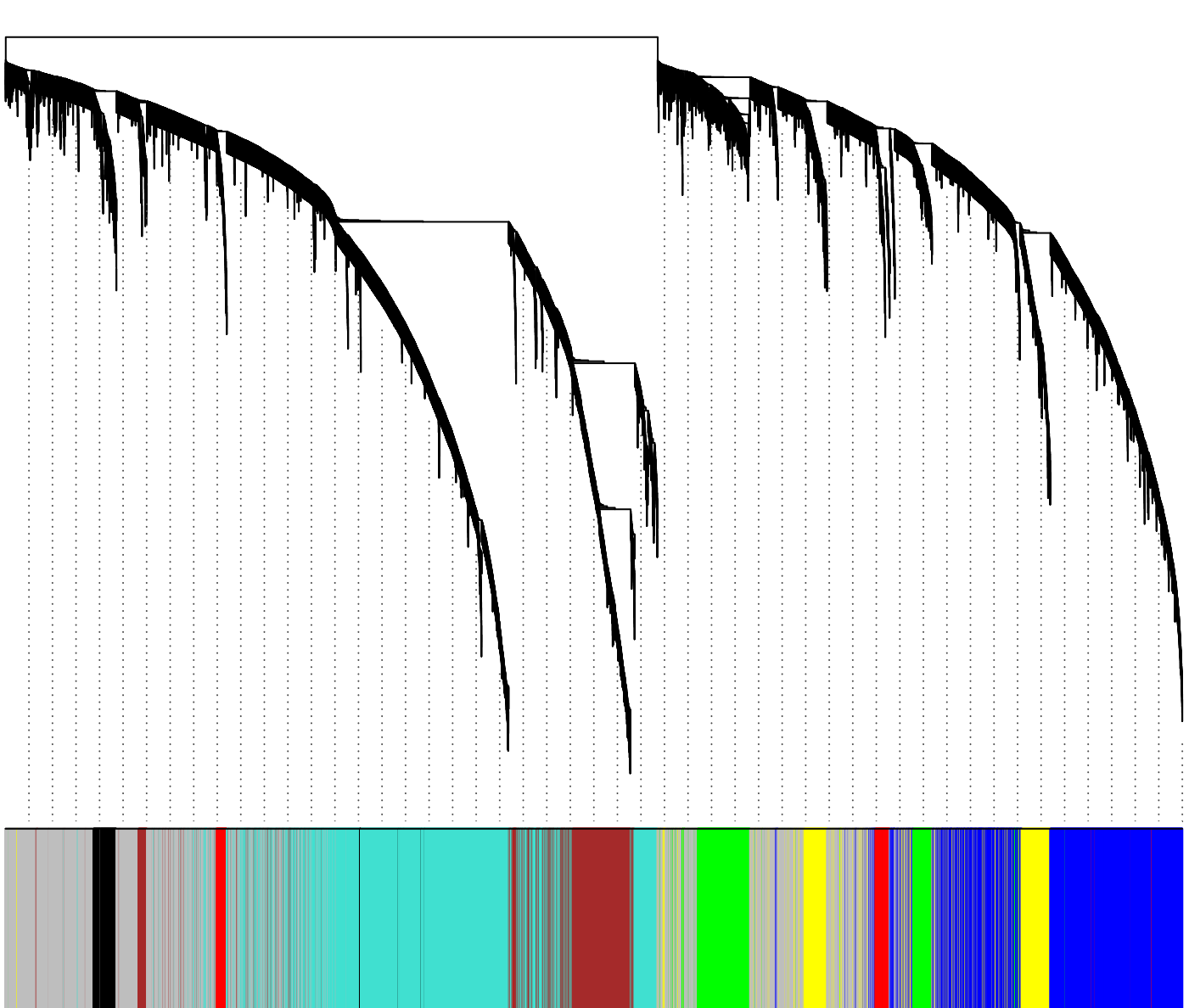

Supplement: Supplemental Information 3 [file peerj-13-20346-s003.zip › supplementary file/03_WGCNA/04.wgcna.dendroColors.pdf]

# Module membership vs. gene significance

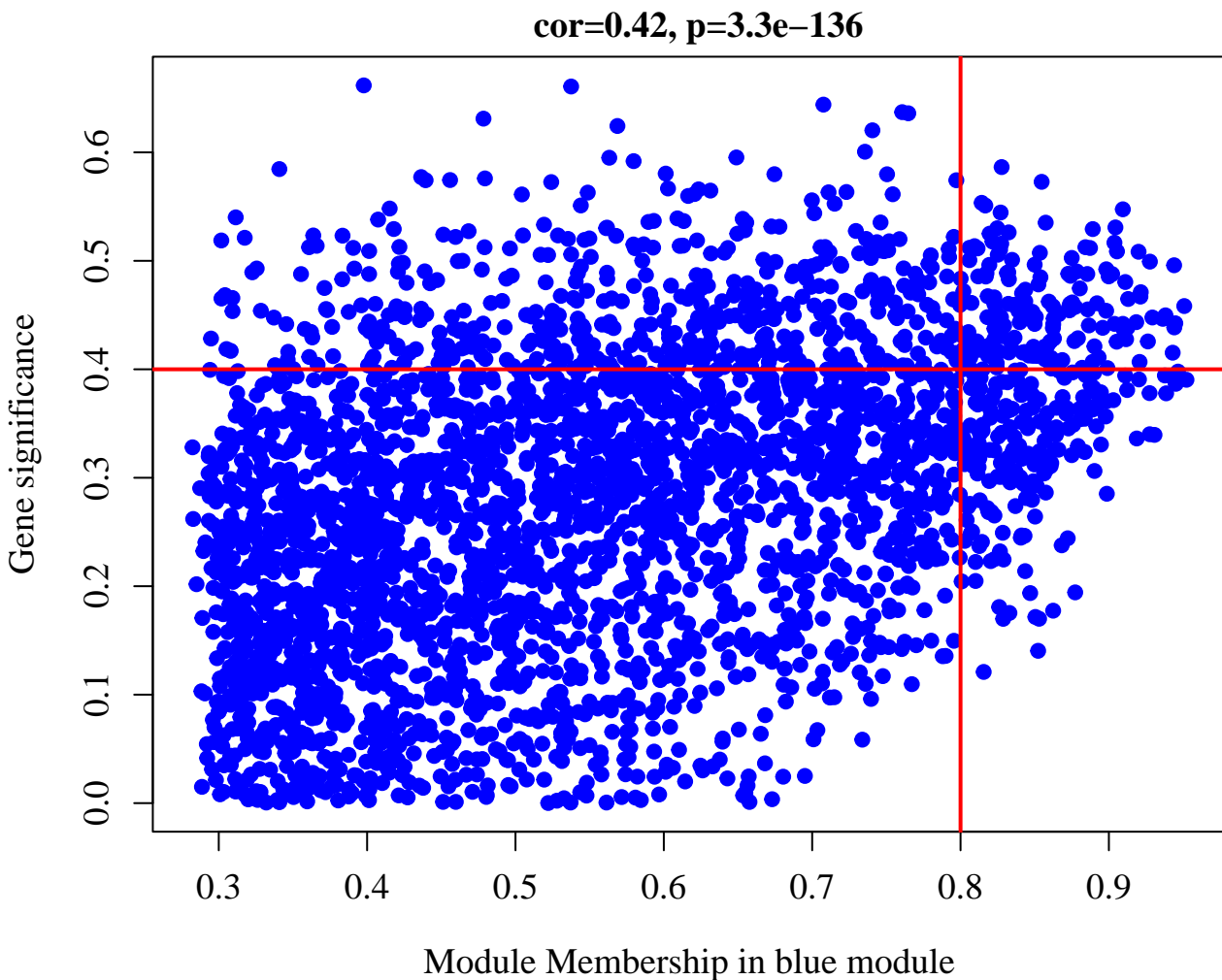

Supplement: Supplemental Information 3 [file peerj-13-20346-s003.zip › supplementary file/03_WGCNA/06.GS_MM.blue.pdf]

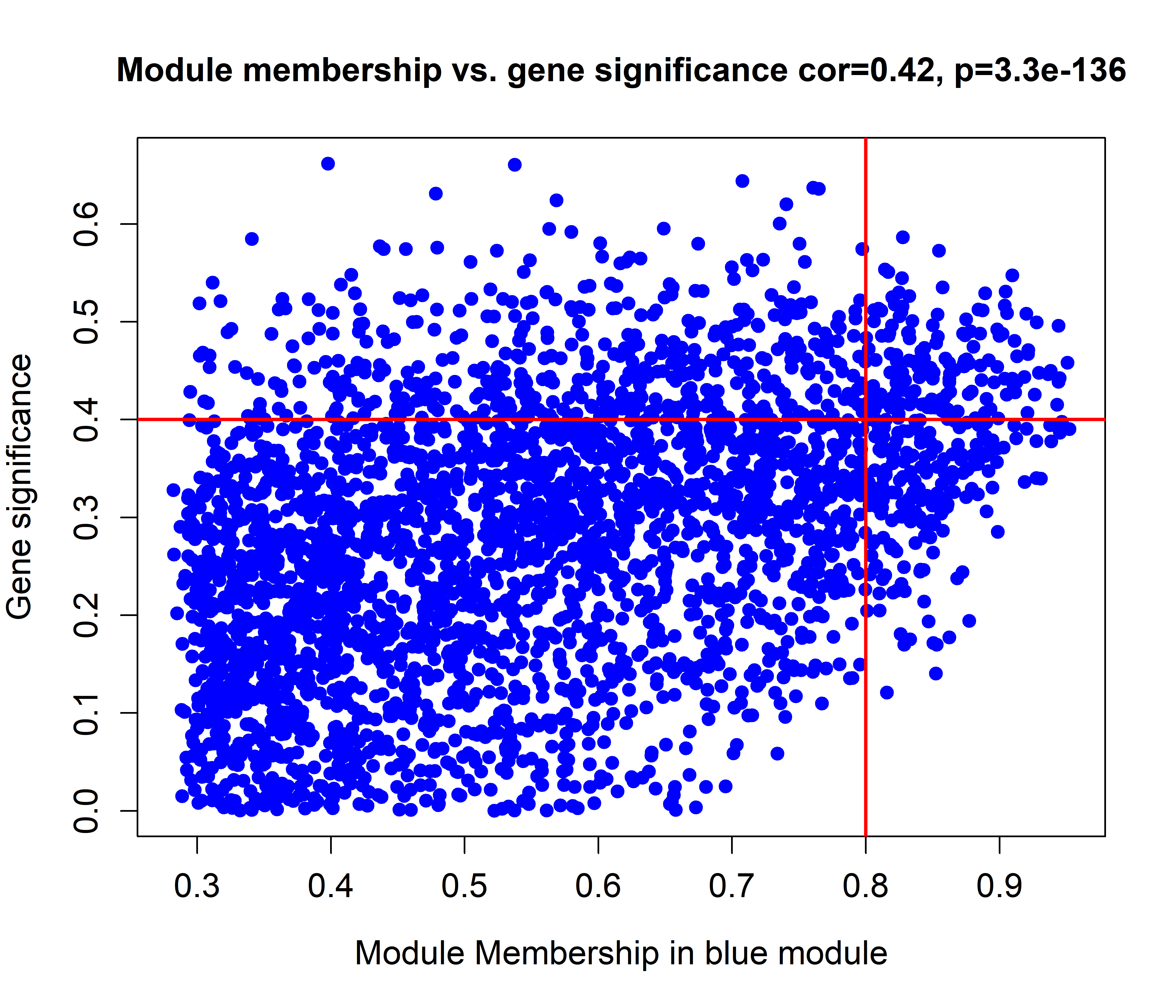

Supplement: Supplemental Information 3 [file peerj-13-20346-s003.zip › supplementary file/03_WGCNA/06.GS_MM.blue.png]

# Module membership vs. gene significance

$\text{cor}=0.89, p<1\text{e-}200$

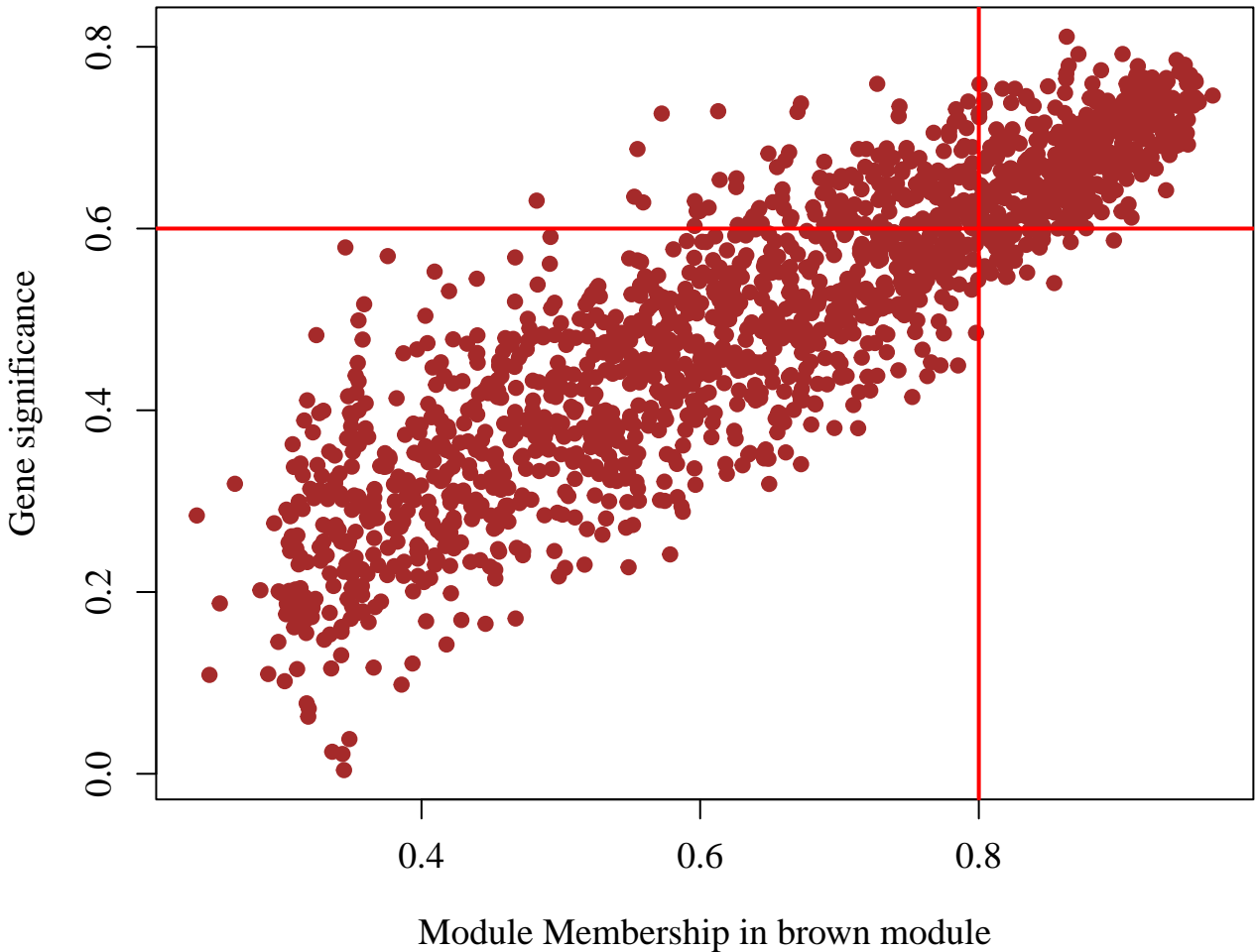

Supplement: Supplemental Information 3 [file peerj-13-20346-s003.zip › supplementary file/03_WGCNA/06.GS_MM.brown.pdf]

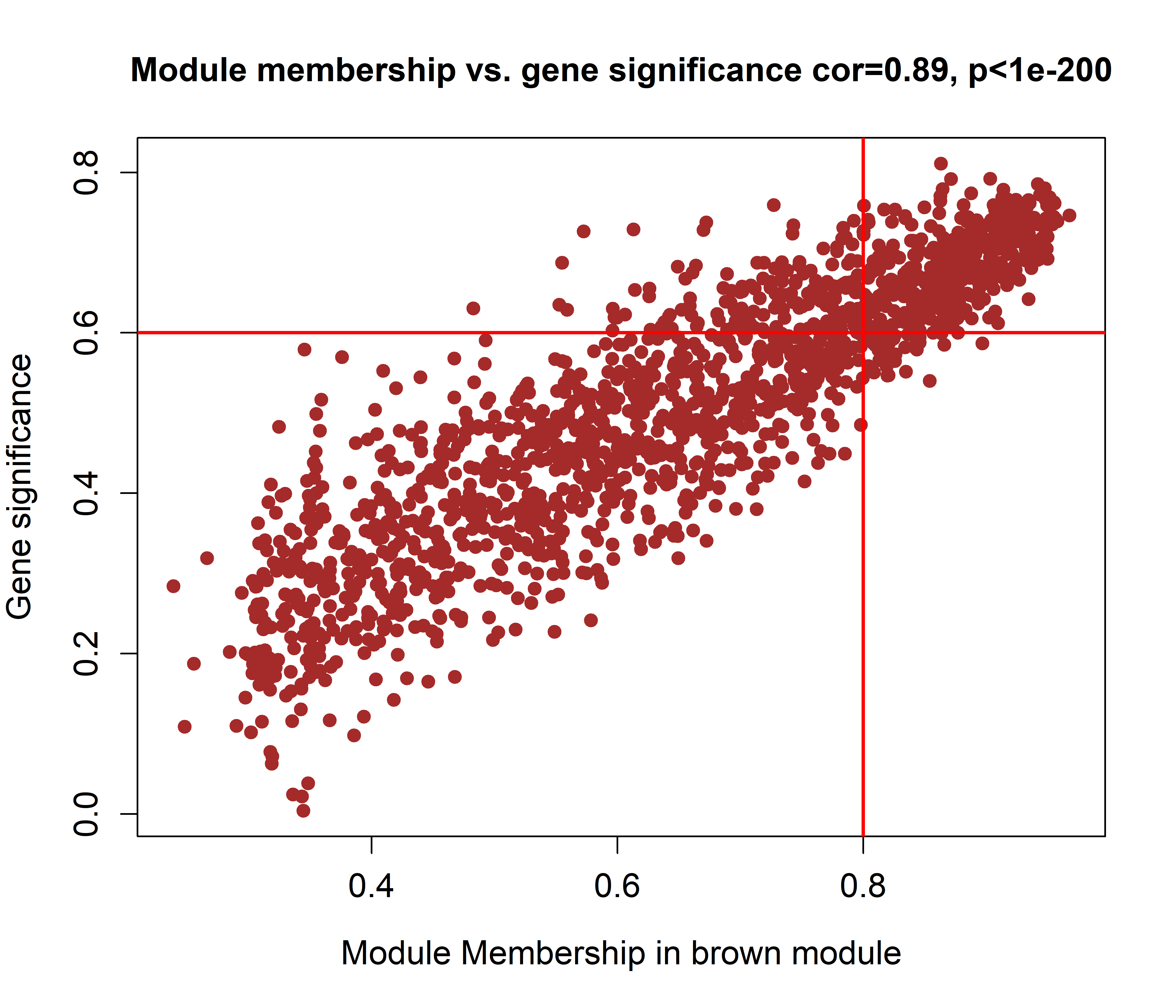

Supplement: Supplemental Information 3 [file peerj-13-20346-s003.zip › supplementary file/03_WGCNA/06.GS_MM.brown.png]

# Module membership vs. gene significance

**cor=0.44, p=1.1e-56**

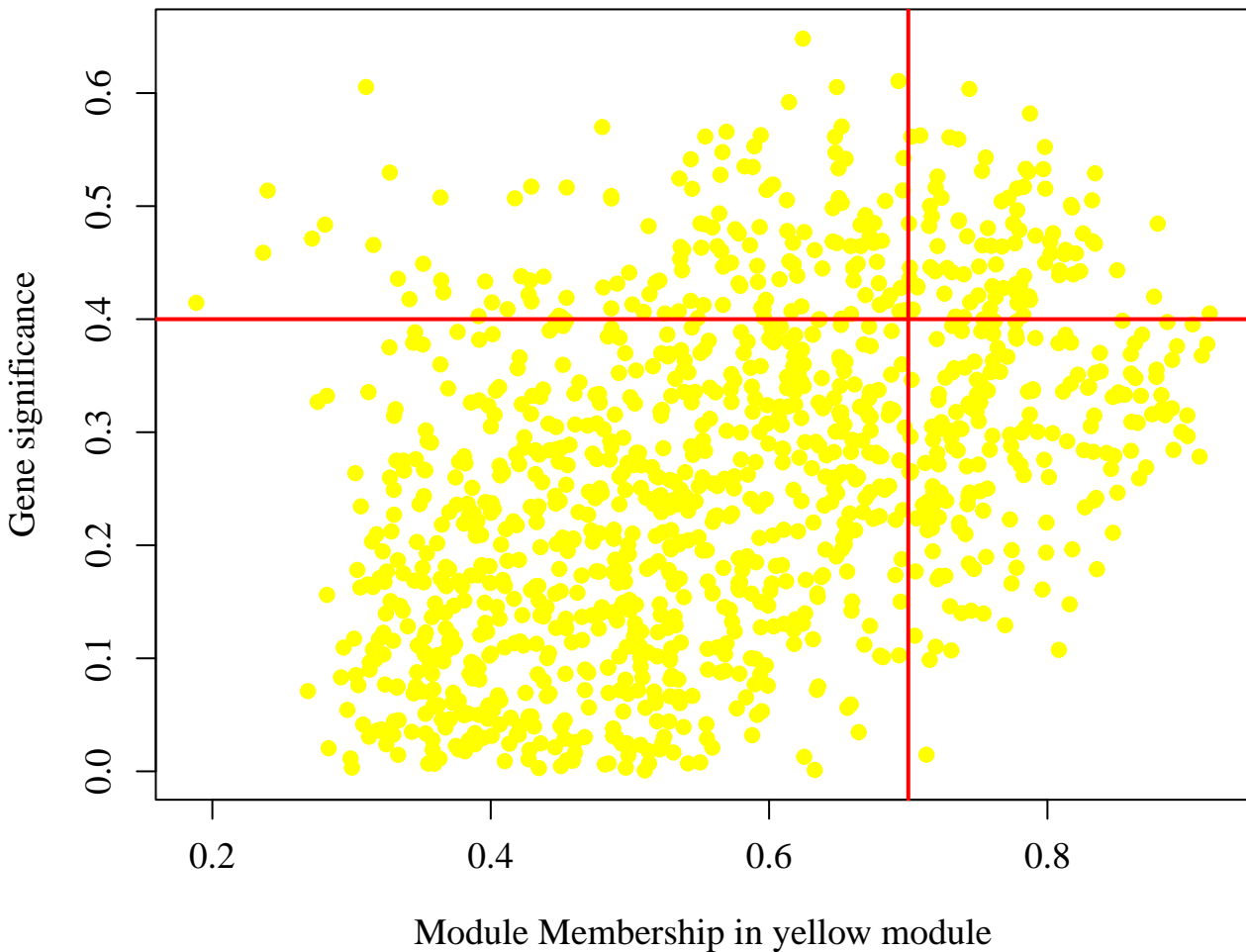

Supplement: Supplemental Information 3 [file peerj-13-20346-s003.zip › supplementary file/03_WGCNA/06.GS_MM.yellow.pdf]

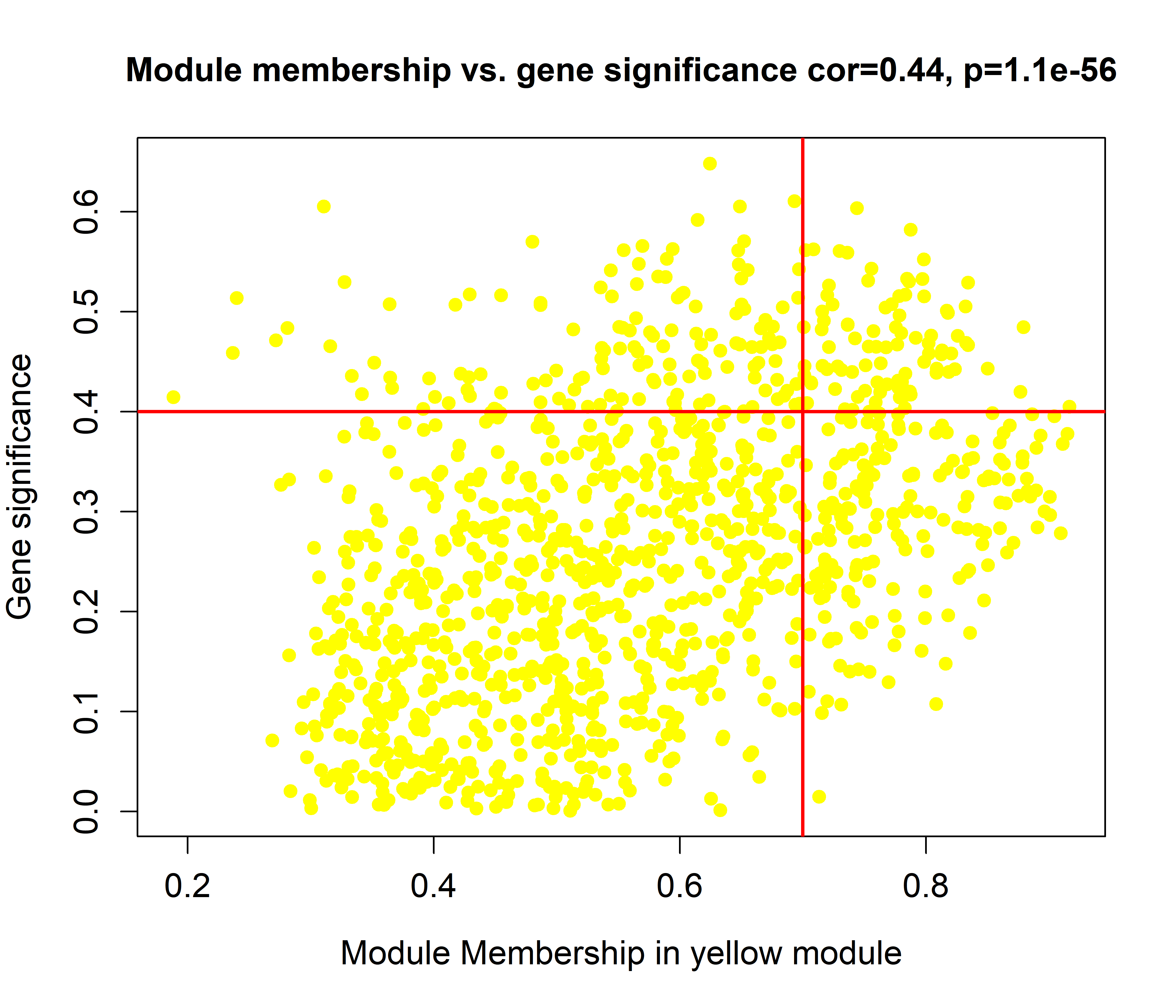

Supplement: Supplemental Information 3 [file peerj-13-20346-s003.zip › supplementary file/03_WGCNA/06.GS_MM.yellow.png]

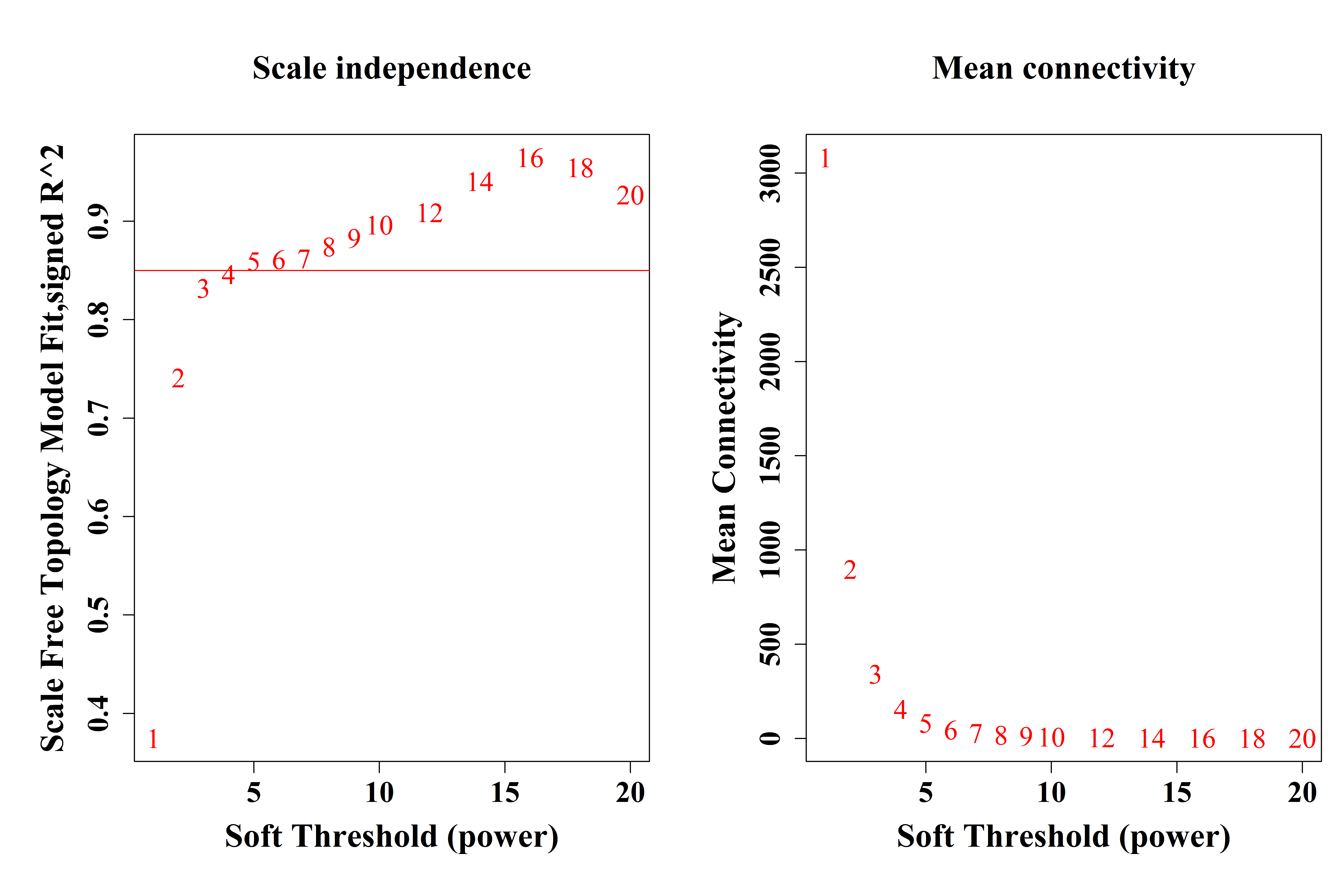

Supplement: Supplemental Information 3 [file peerj-13-20346-s003.zip › supplementary file/03_WGCNA/03.softThreshold.png]

Module–trait relationships

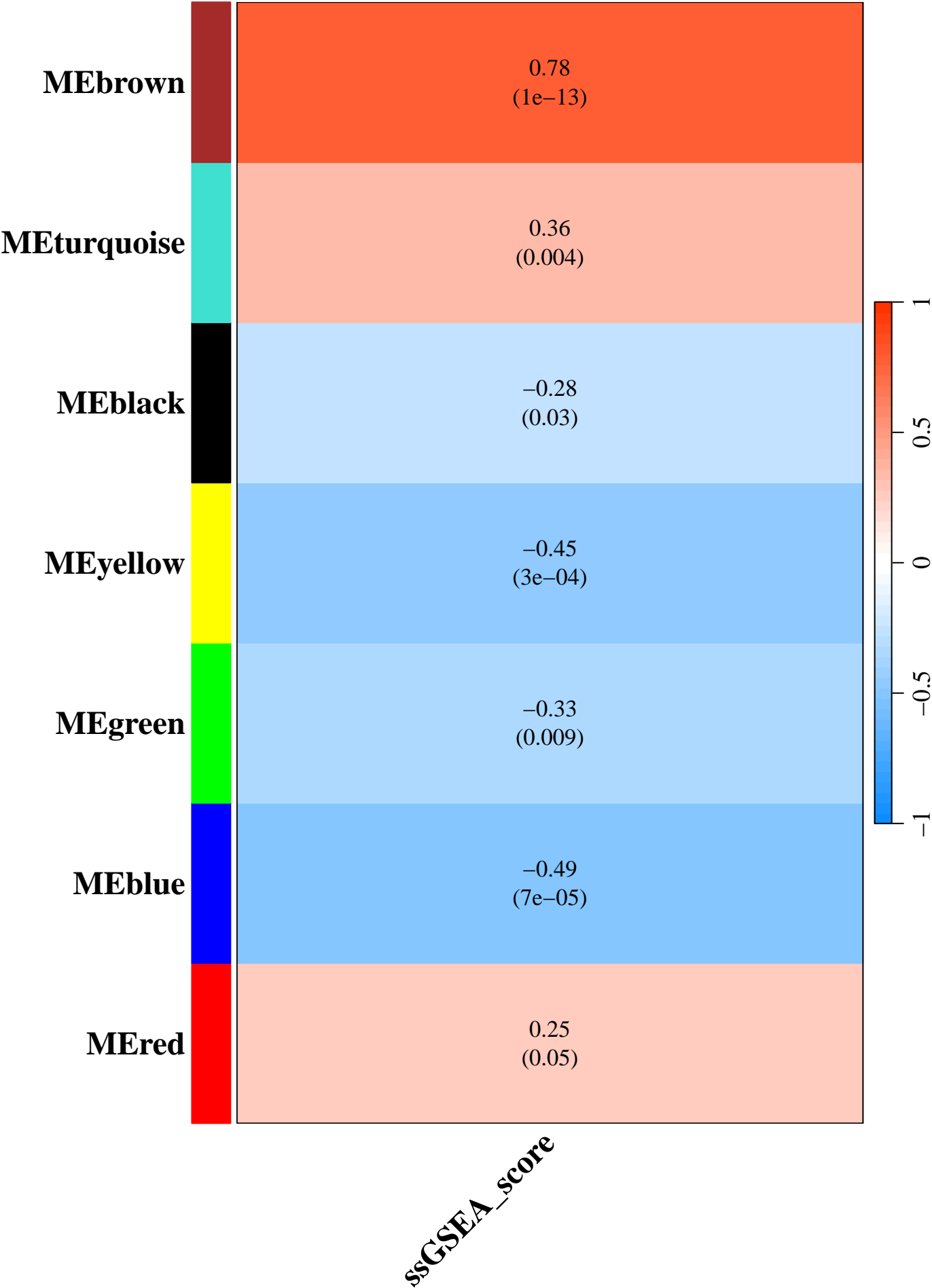

Supplement: Supplemental Information 3 [file peerj-13-20346-s003.zip › supplementary file/03_WGCNA/05.wgcna.Module-trait.heatmap.pdf]

**Scale independence**

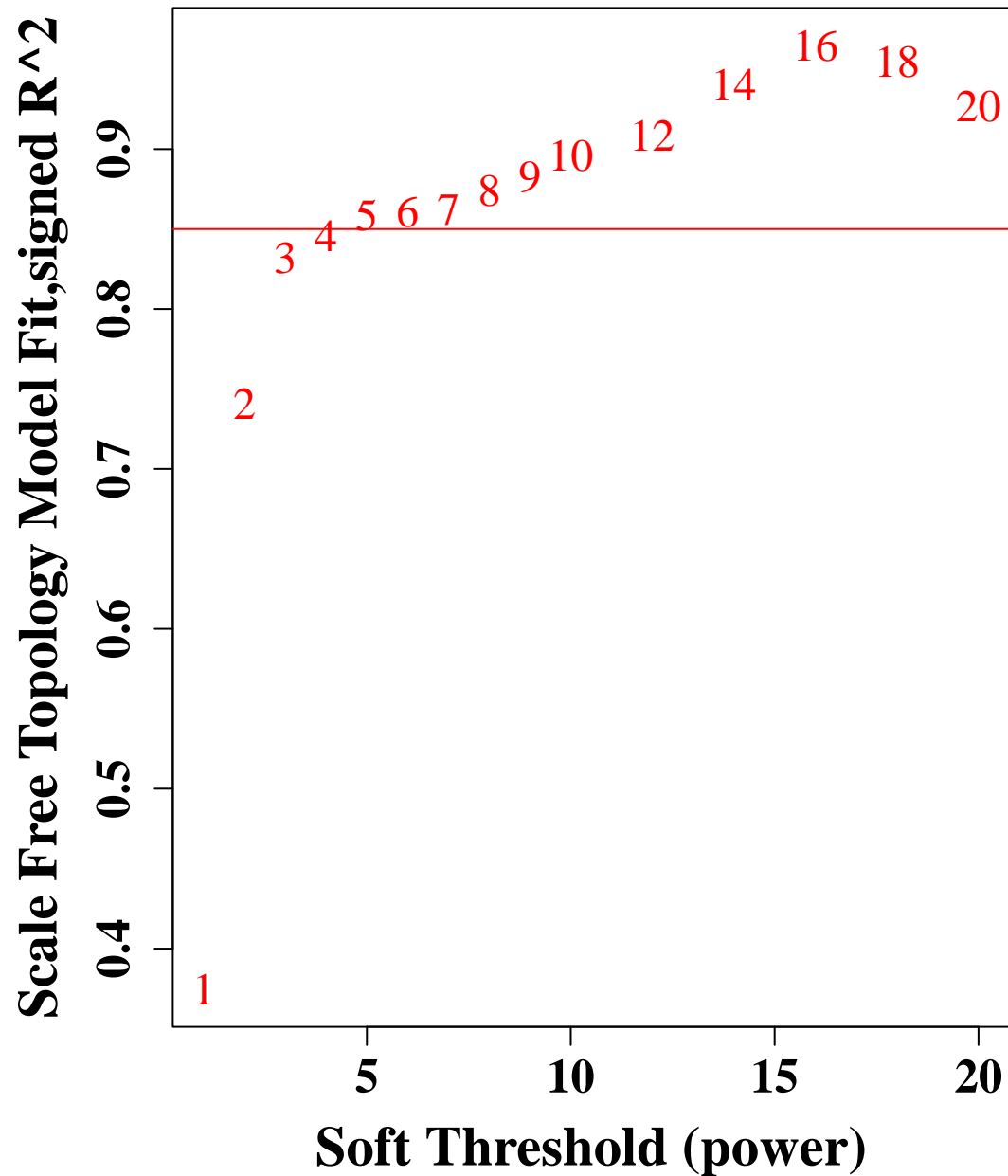

**Mean connectivity**

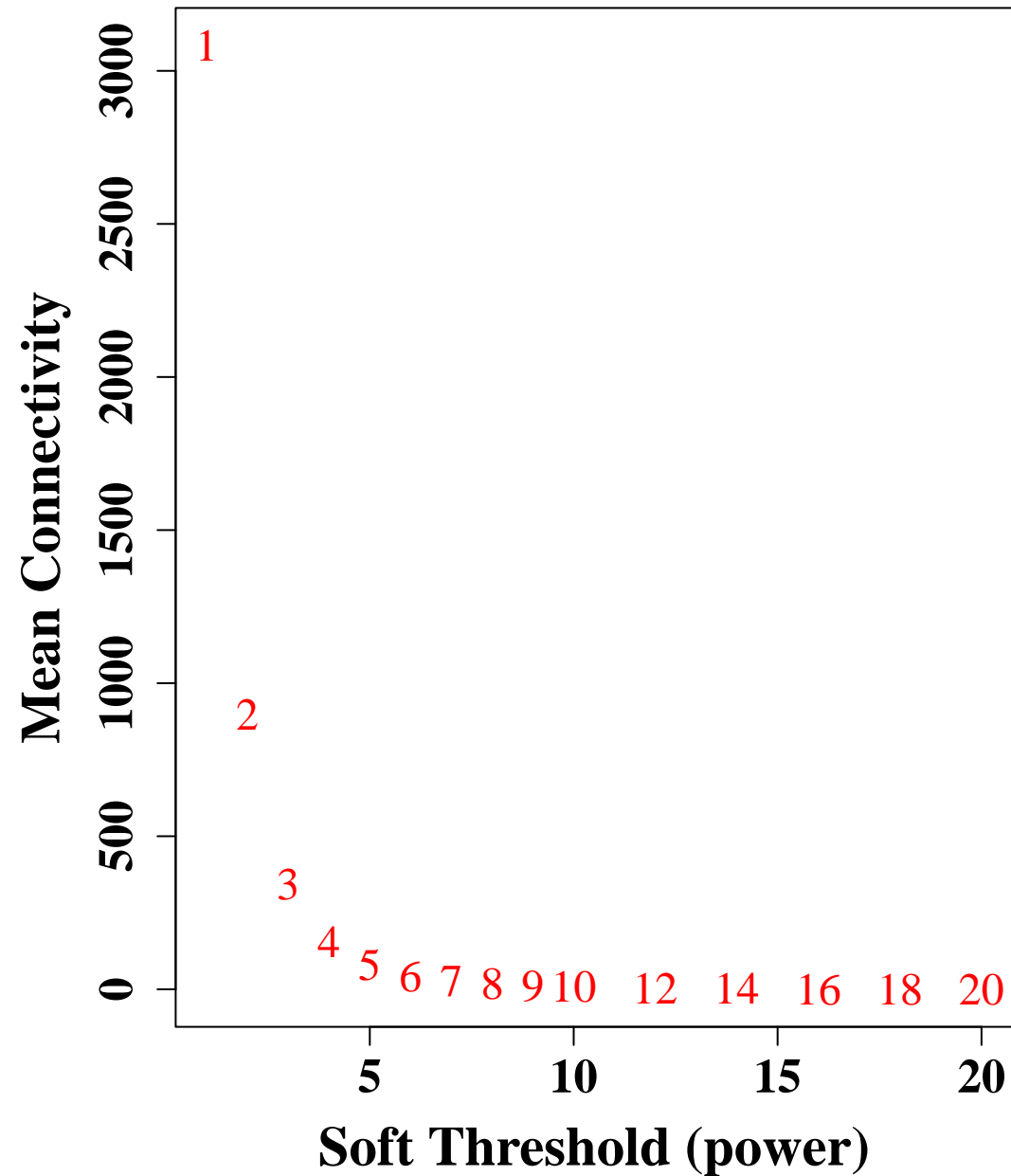

Supplement: Supplemental Information 3 [file peerj-13-20346-s003.zip › supplementary file/03_WGCNA/03.softThreshold.pdf]

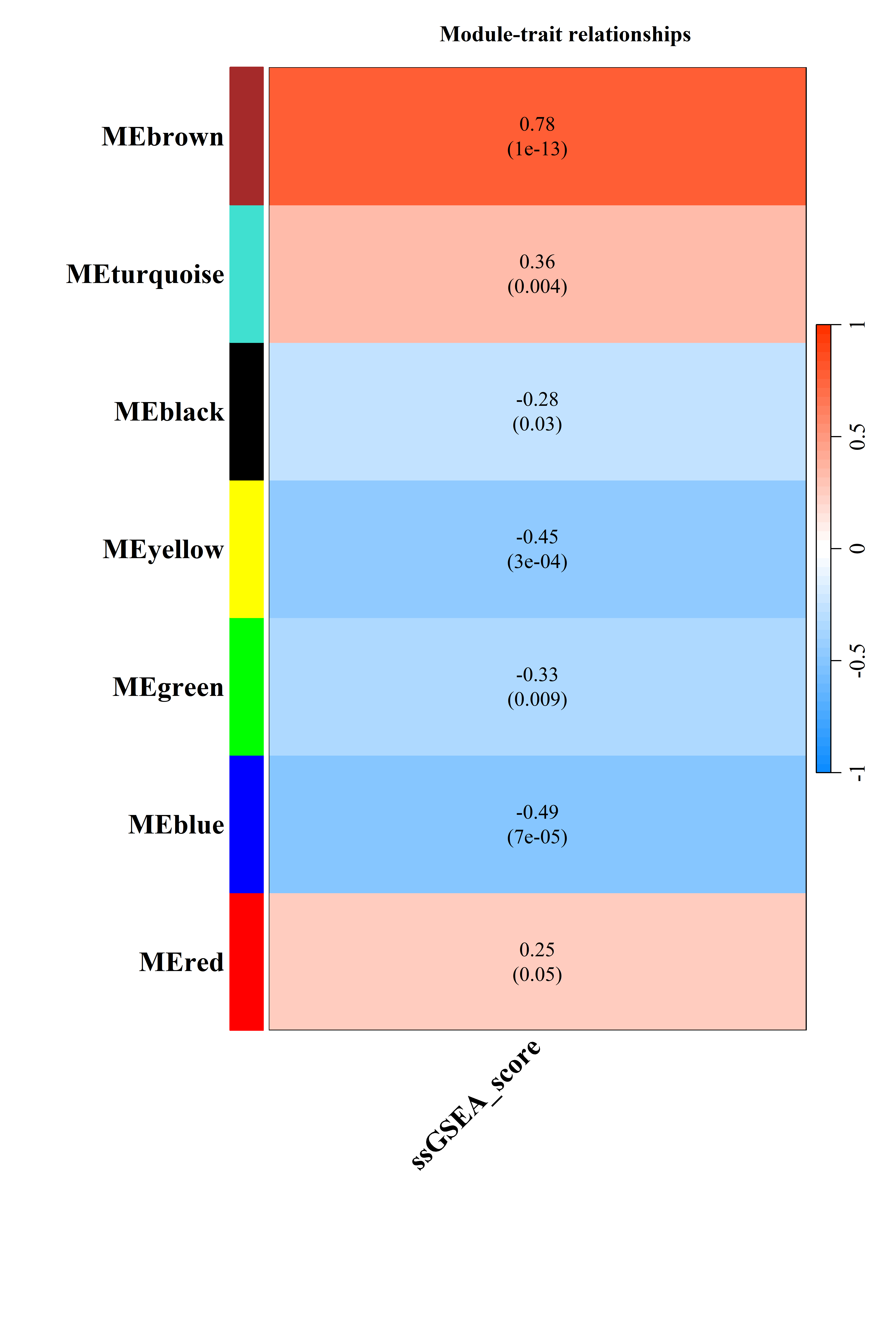

Supplement: Supplemental Information 3 [file peerj-13-20346-s003.zip › supplementary file/03_WGCNA/05.wgcna.Module-trait.heatmap.png]

# Sample Clustering and trait heatmap

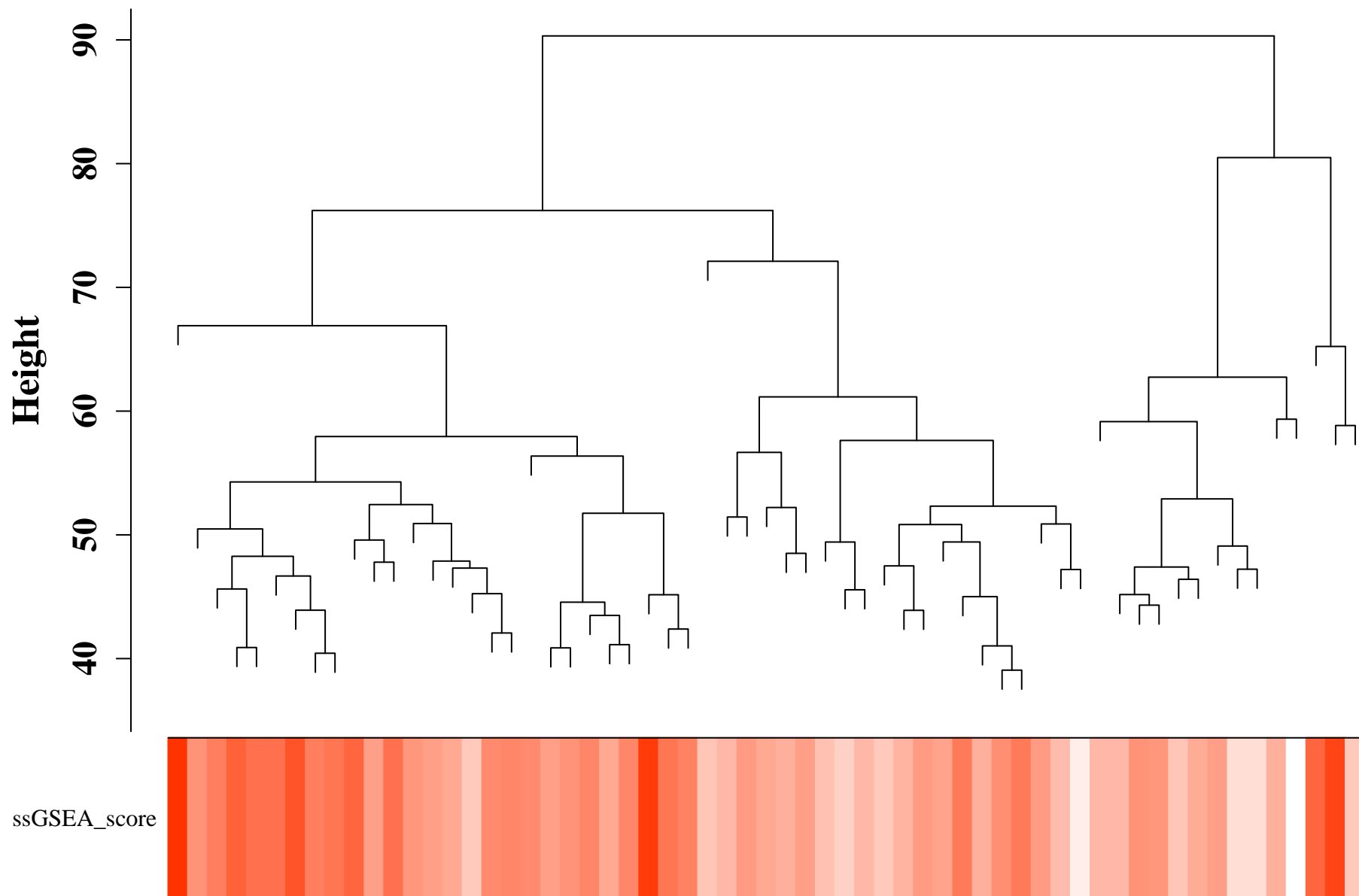

Supplement: Supplemental Information 3 [file peerj-13-20346-s003.zip › supplementary file/03_WGCNA/02.sampleClustering2.pdf]

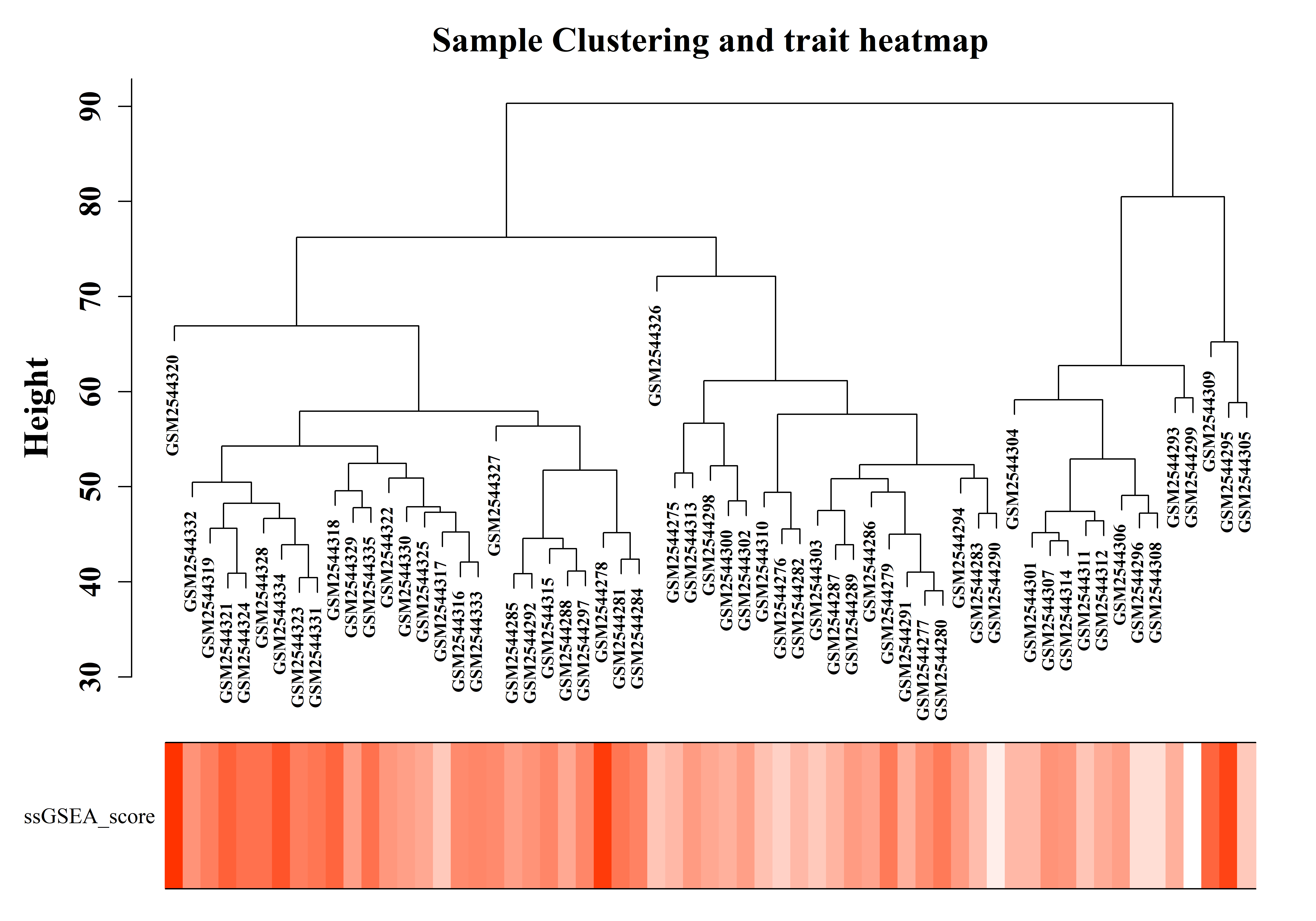

Supplement: Supplemental Information 3 [file peerj-13-20346-s003.zip › supplementary file/03_WGCNA/02.sampleClustering2.png]

# Sample Clustering

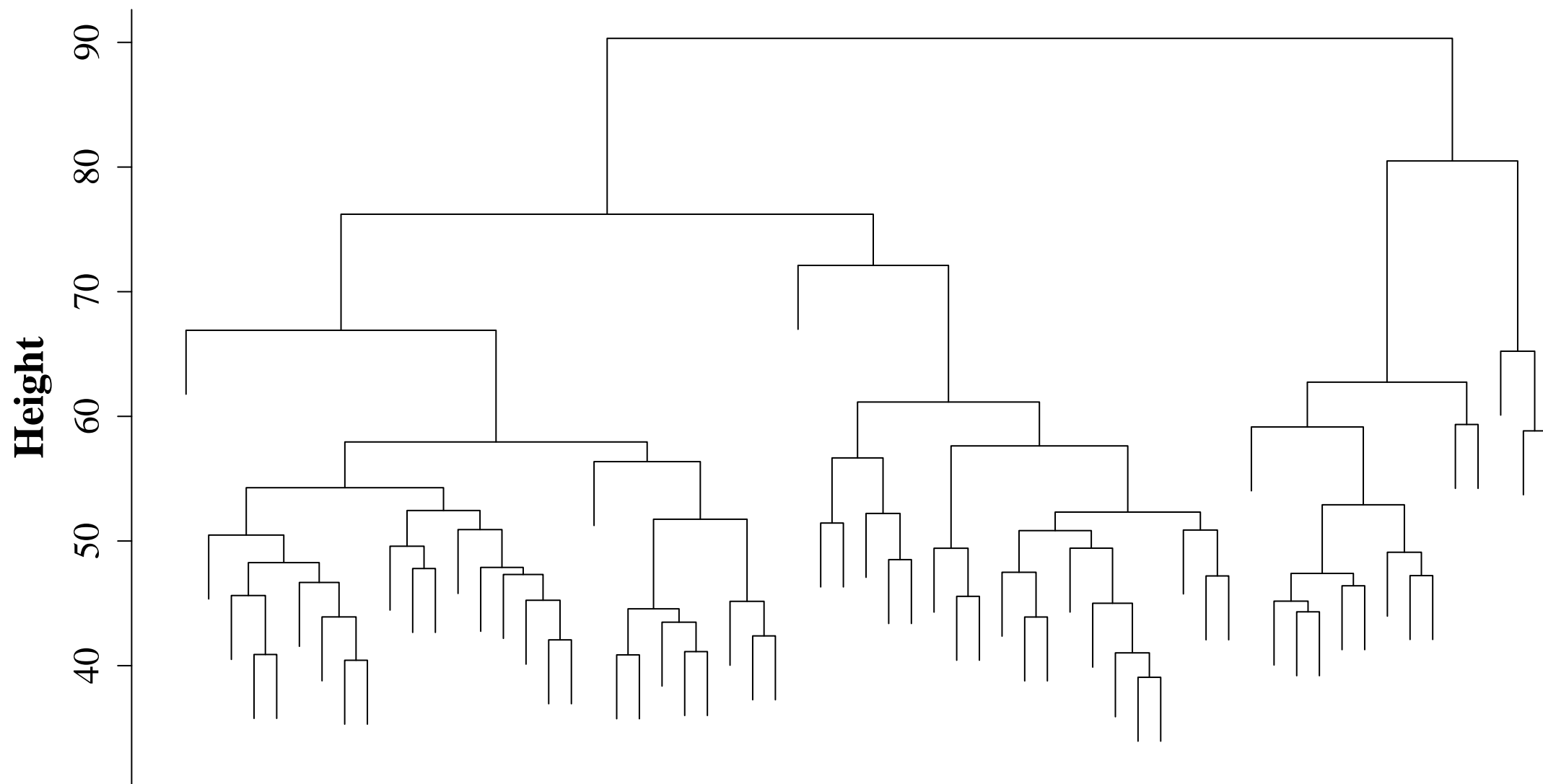

Supplement: Supplemental Information 3 [file peerj-13-20346-s003.zip › supplementary file/03_WGCNA/01.sampleClustering.pdf]

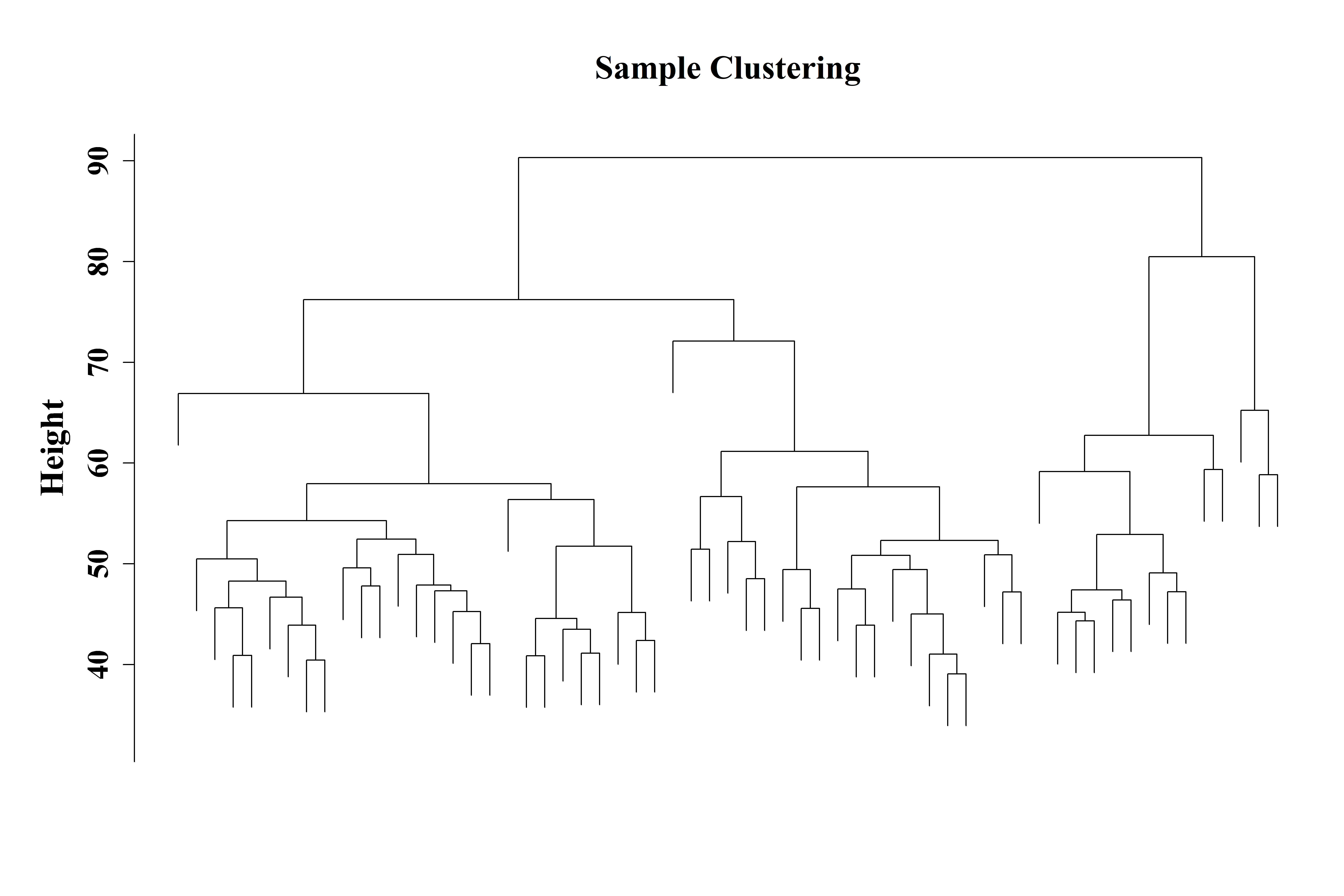

Supplement: Supplemental Information 3 [file peerj-13-20346-s003.zip › supplementary file/03_WGCNA/01.sampleClustering.png]

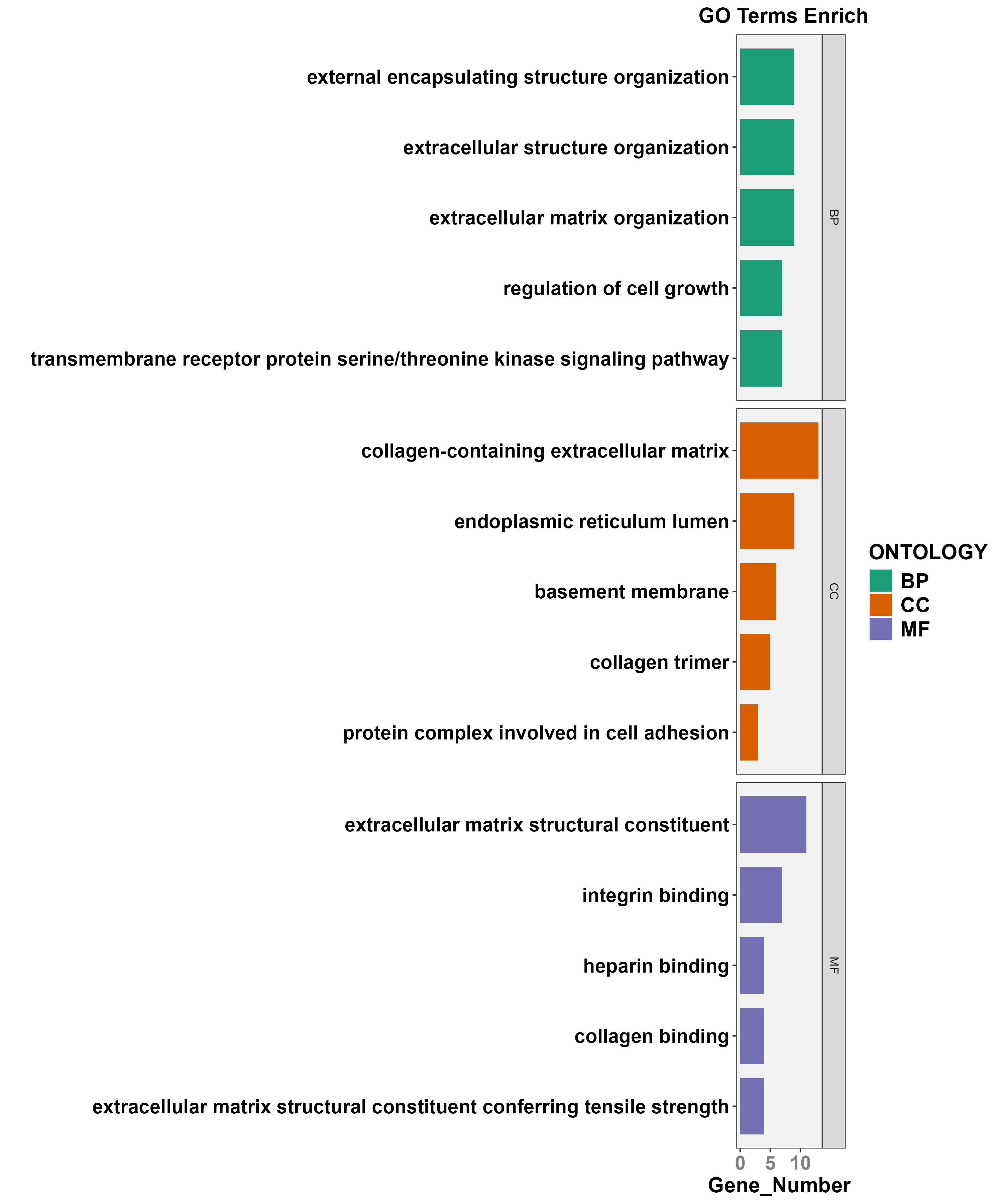

Supplement: Supplemental Information 3 [file peerj-13-20346-s003.zip › supplementary file/09_enrichment/GO/02.go_sig_vertical_bar.png]

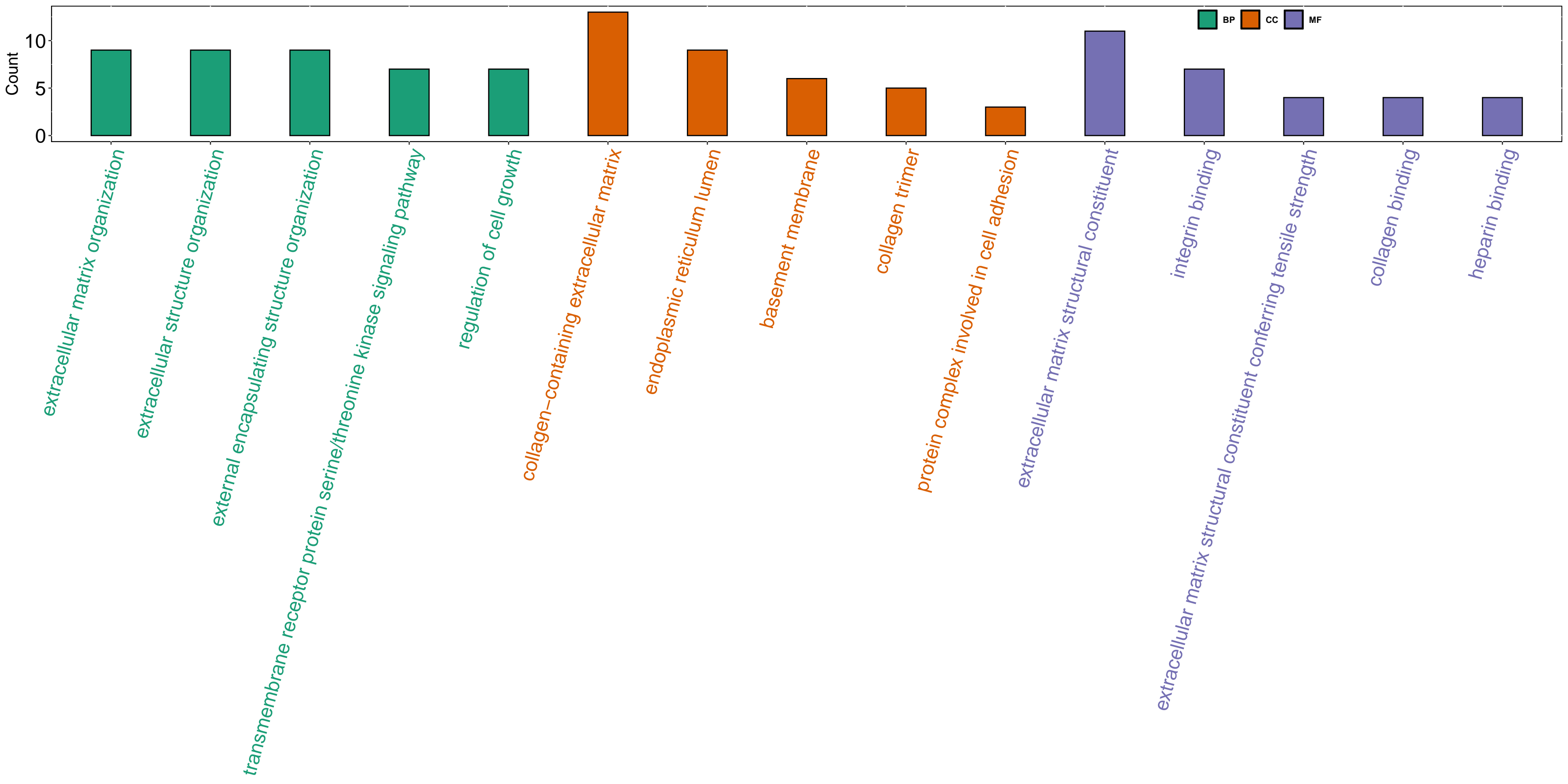

Supplement: Supplemental Information 3 [file peerj-13-20346-s003.zip › supplementary file/09_enrichment/GO/01.go_sig_Horizontal_bar.pdf]

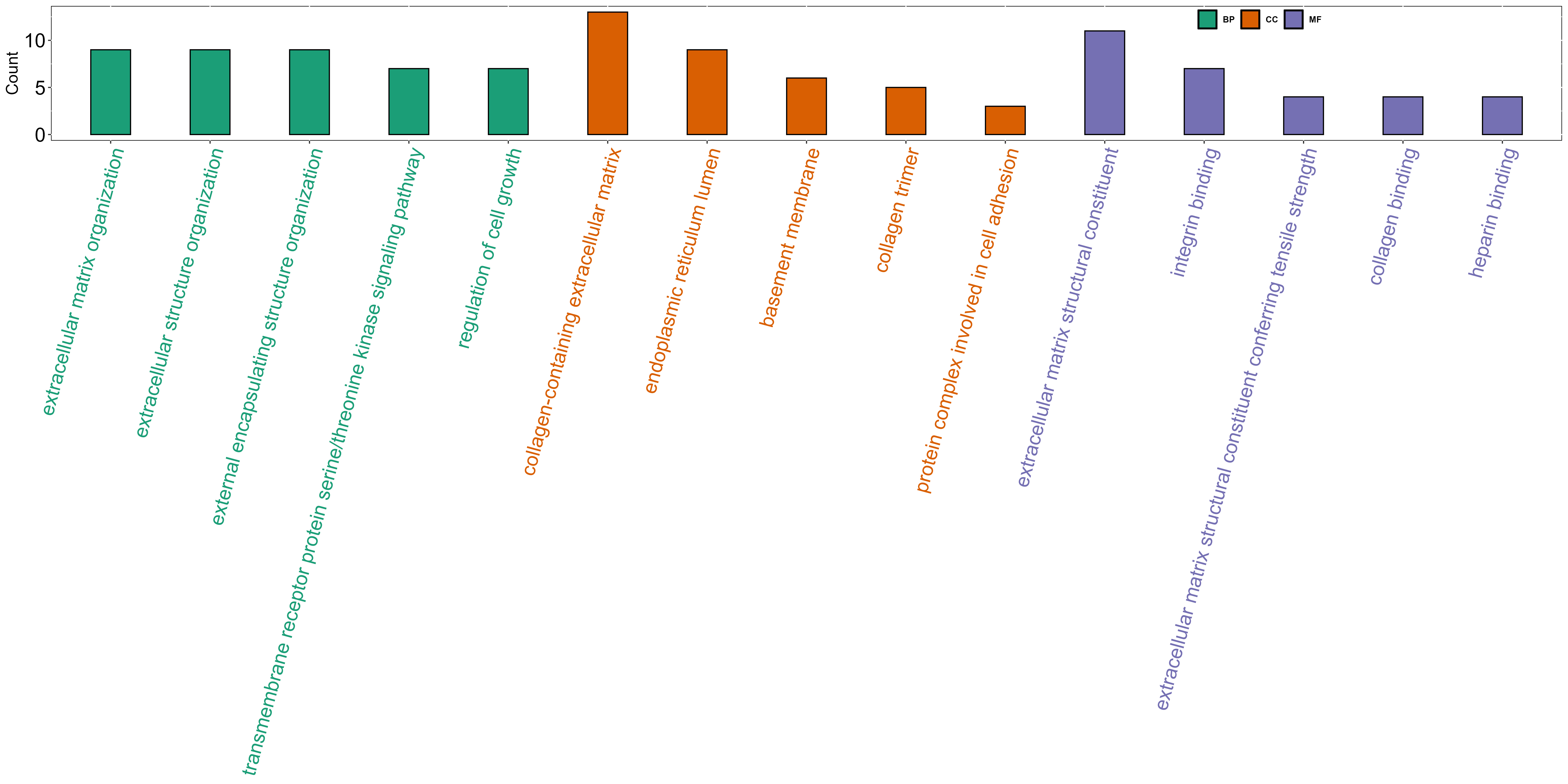

Supplement: Supplemental Information 3 [file peerj-13-20346-s003.zip › supplementary file/09_enrichment/GO/01.go_sig_Horizontal_bar.png]

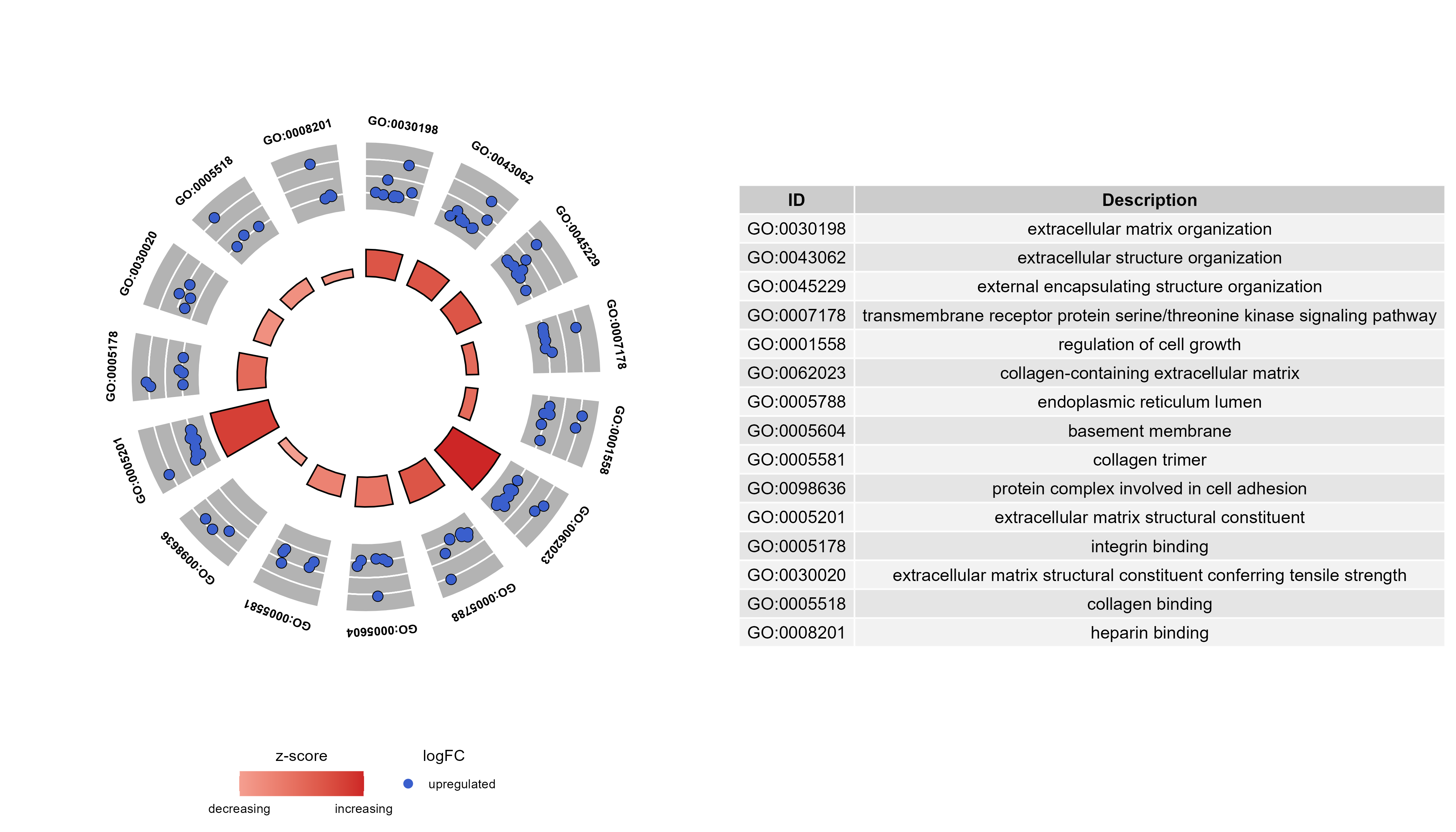

Supplement: Supplemental Information 3 [file peerj-13-20346-s003.zip › supplementary file/09_enrichment/GO/03.go_circle.png]

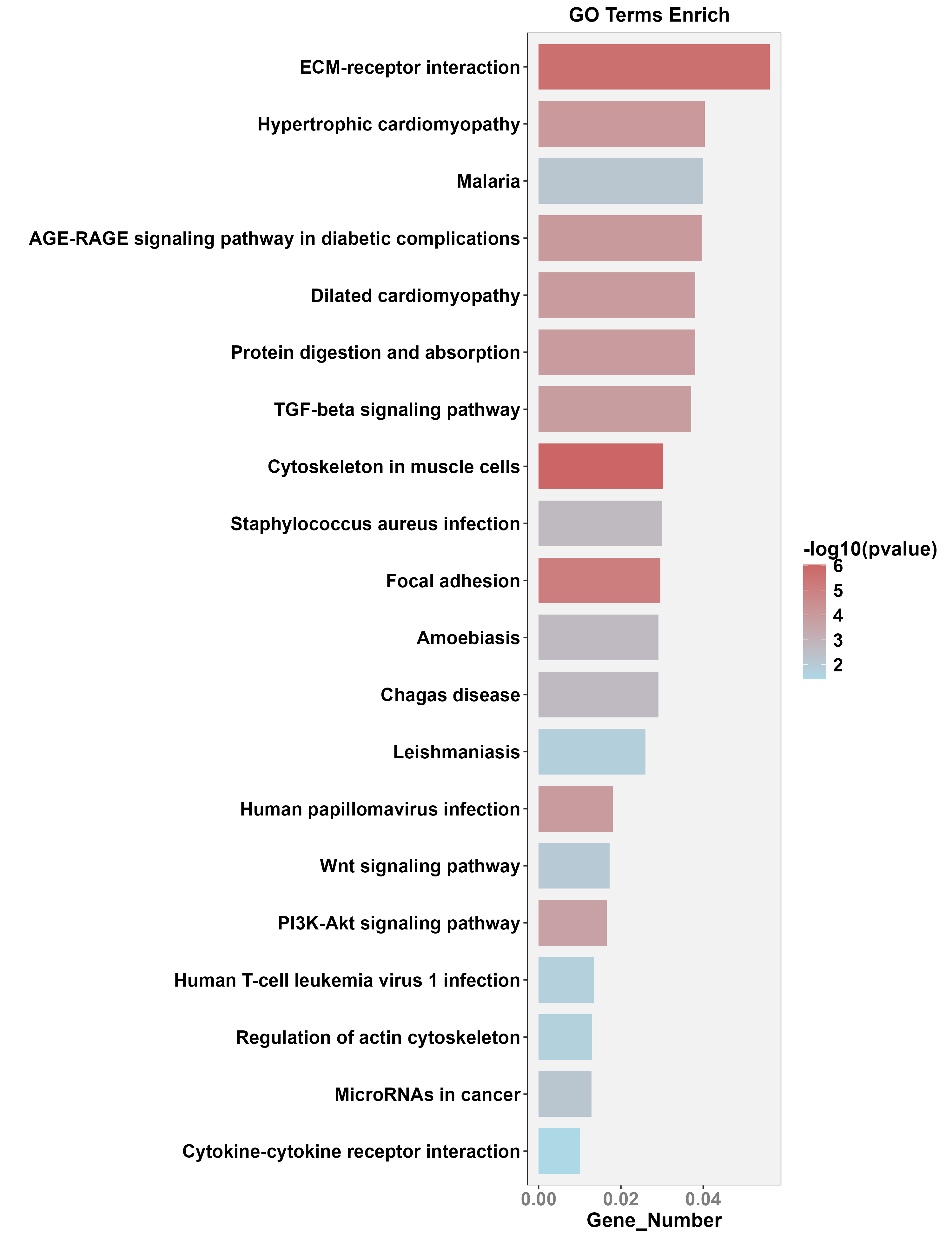

Supplement: Supplemental Information 3 [file peerj-13-20346-s003.zip › supplementary file/09_enrichment/KEGG/02.kegg_res_vertical_bar.png]

# GO Terms Enrich

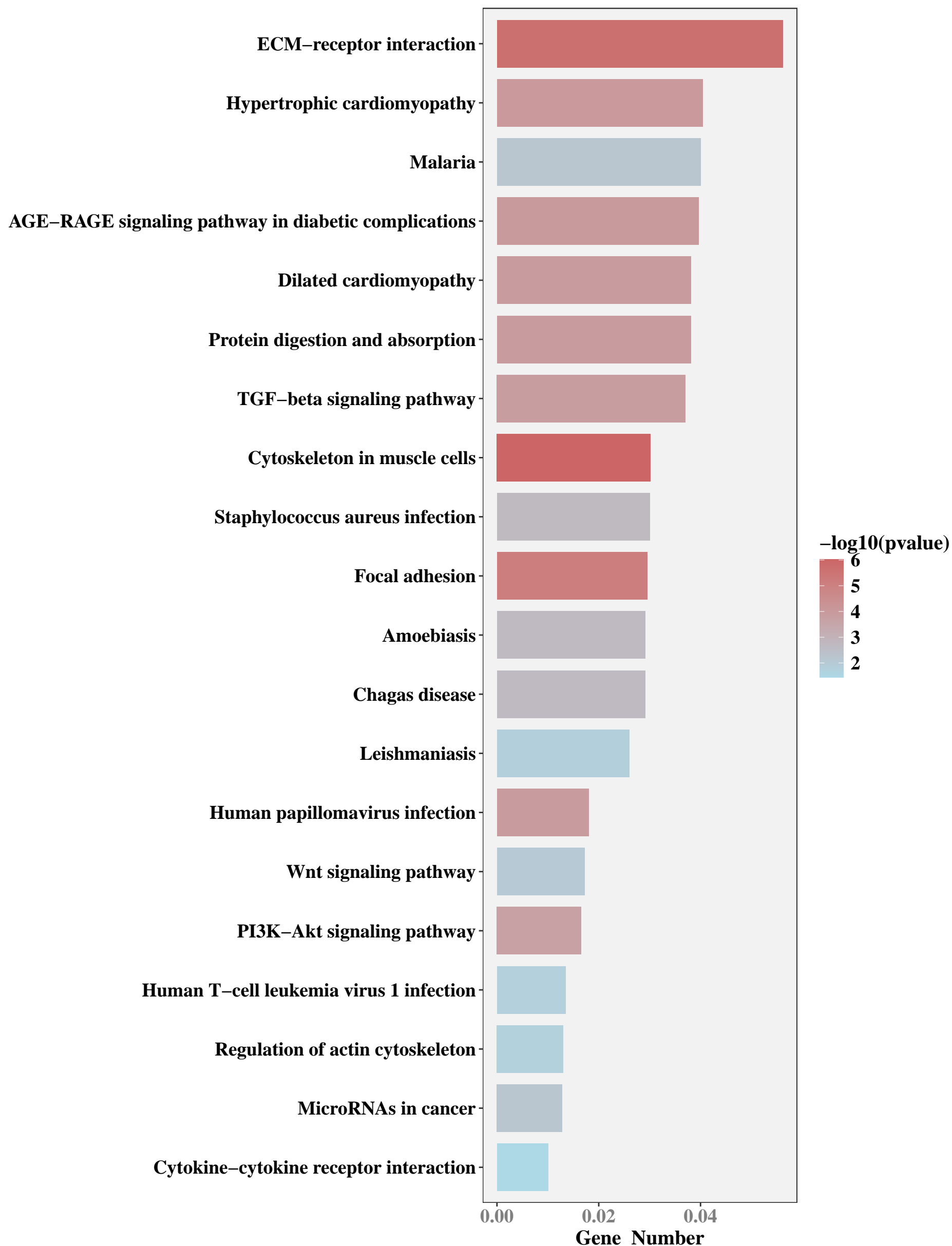

Supplement: Supplemental Information 3 [file peerj-13-20346-s003.zip › supplementary file/09_enrichment/KEGG/02.kegg_res_vertical_bar.pdf]

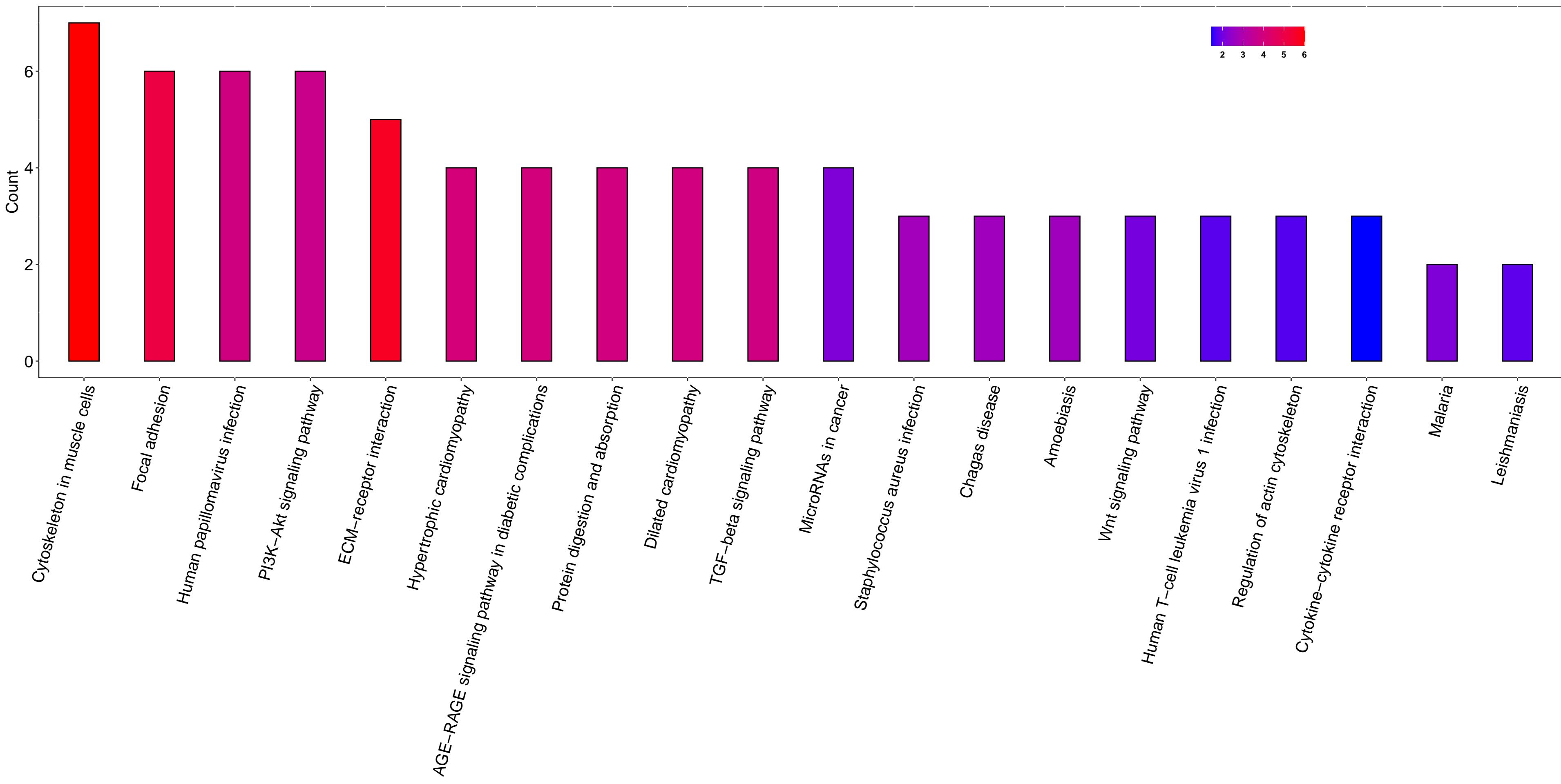

Supplement: Supplemental Information 3 [file peerj-13-20346-s003.zip › supplementary file/09_enrichment/KEGG/01.kegg_res_Horizontal_bar.pdf]

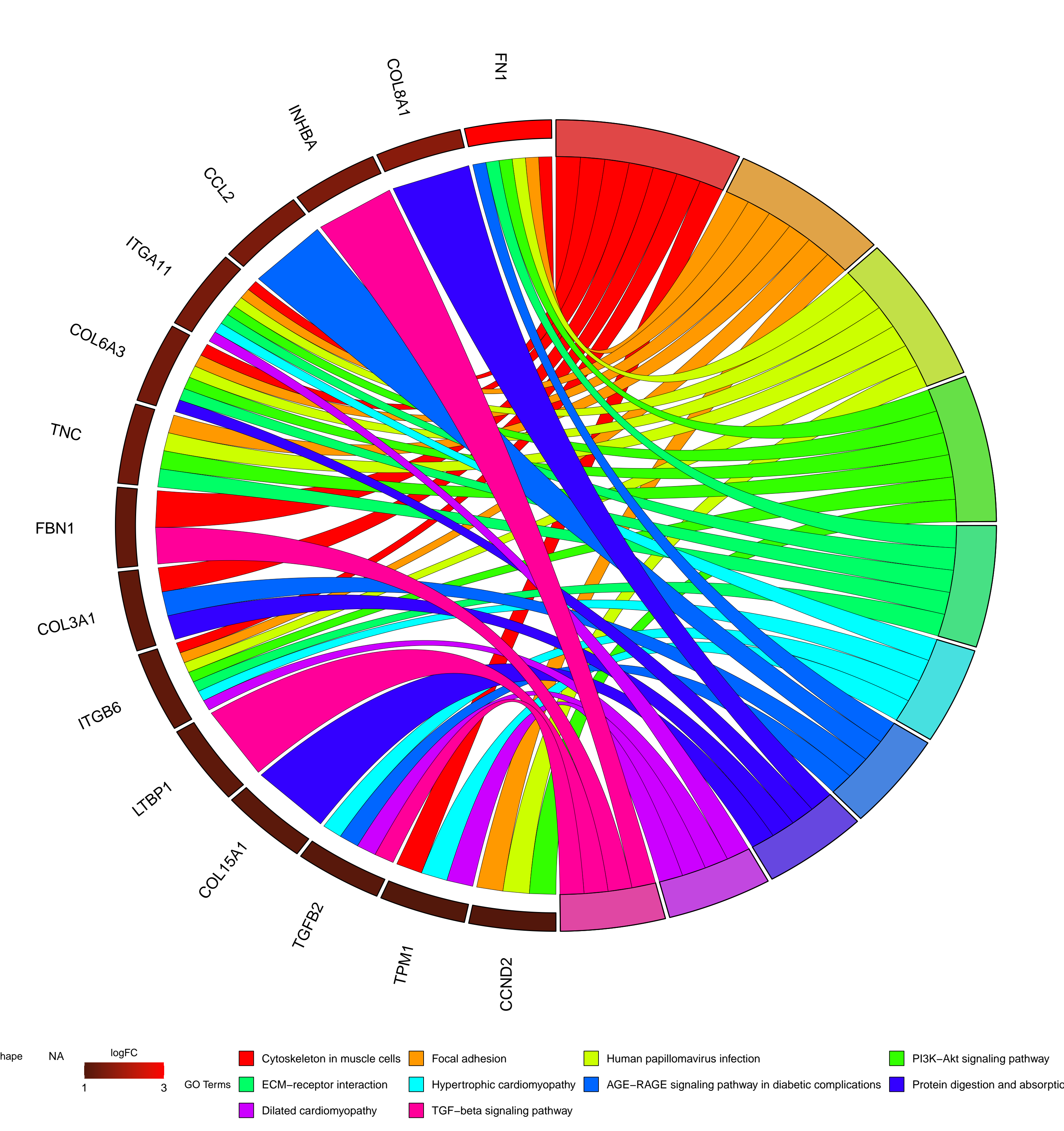

Supplement: Supplemental Information 3 [file peerj-13-20346-s003.zip › supplementary file/09_enrichment/KEGG/KEGG_chord.pdf]

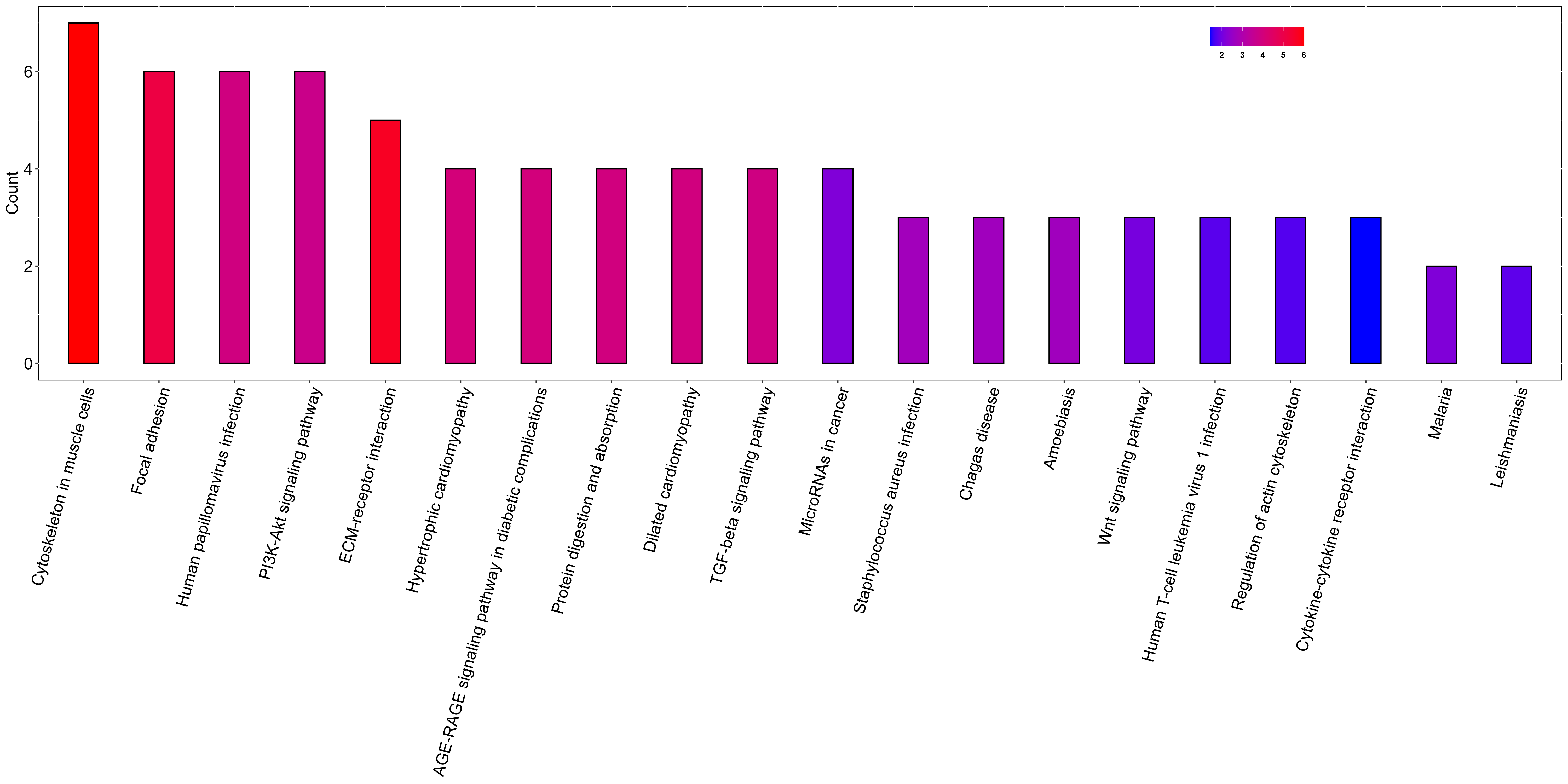

Supplement: Supplemental Information 3 [file peerj-13-20346-s003.zip › supplementary file/09_enrichment/KEGG/01.kegg_res_Horizontal_bar.png]

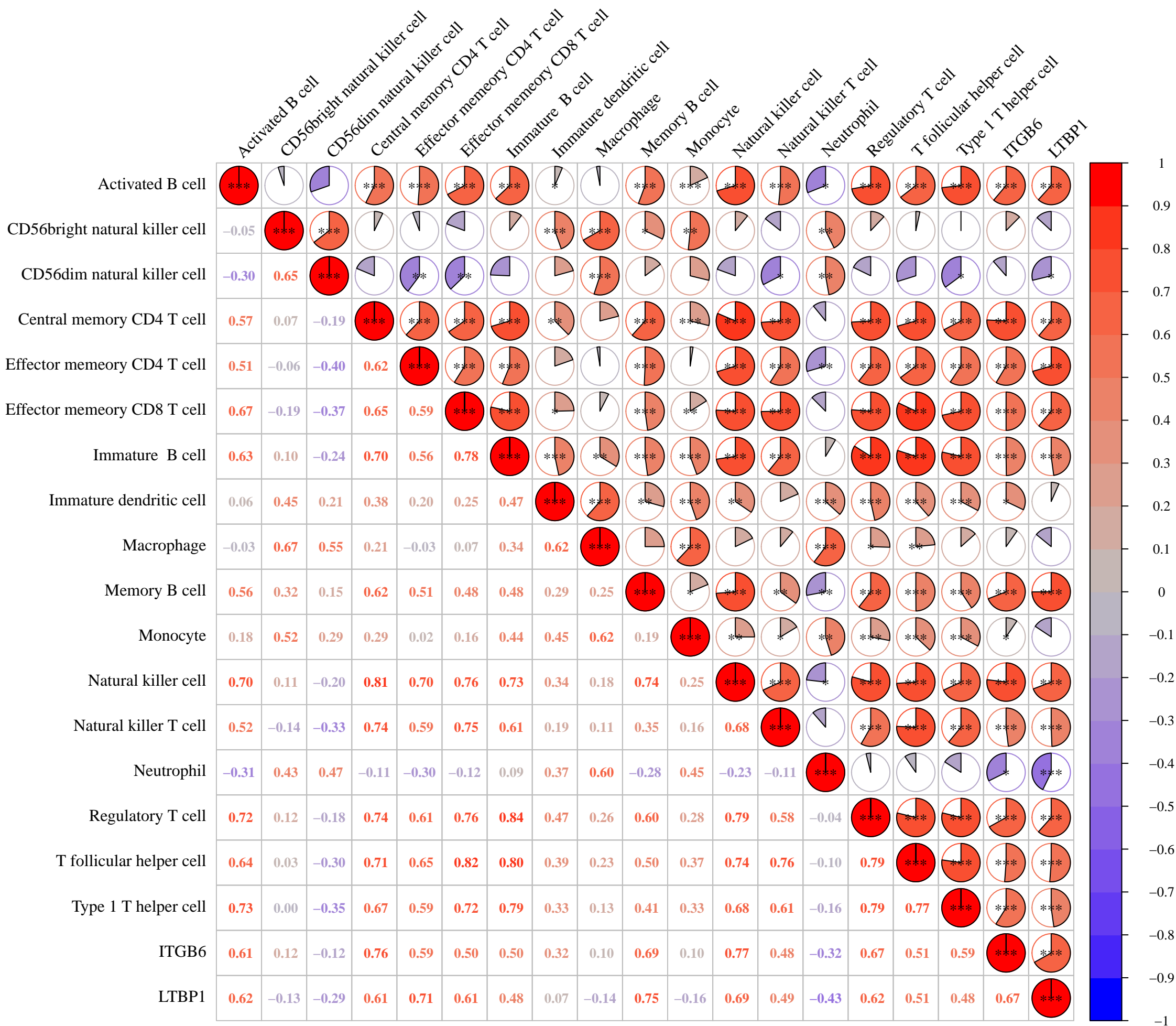

Supplement: Supplemental Information 3 [file peerj-13-20346-s003.zip › supplementary file/11_immune_infiltration/ssGSEA/03.cor_heatmap.pdf]

# ssGSEA

Group ■ disease ■ normal

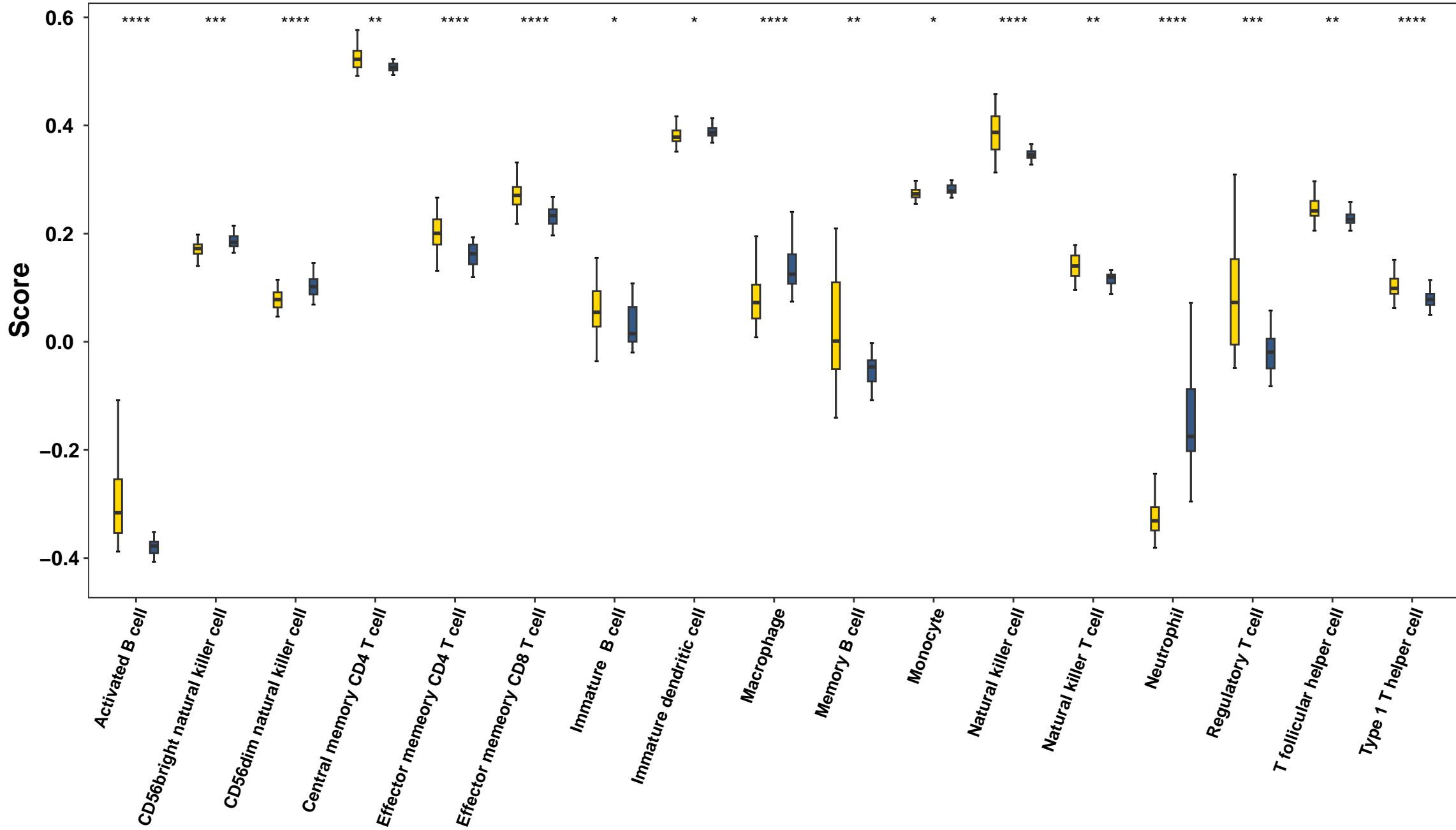

Supplement: Supplemental Information 3 [file peerj-13-20346-s003.zip › supplementary file/11_immune_infiltration/ssGSEA/01.immue_cell_bar.pdf]

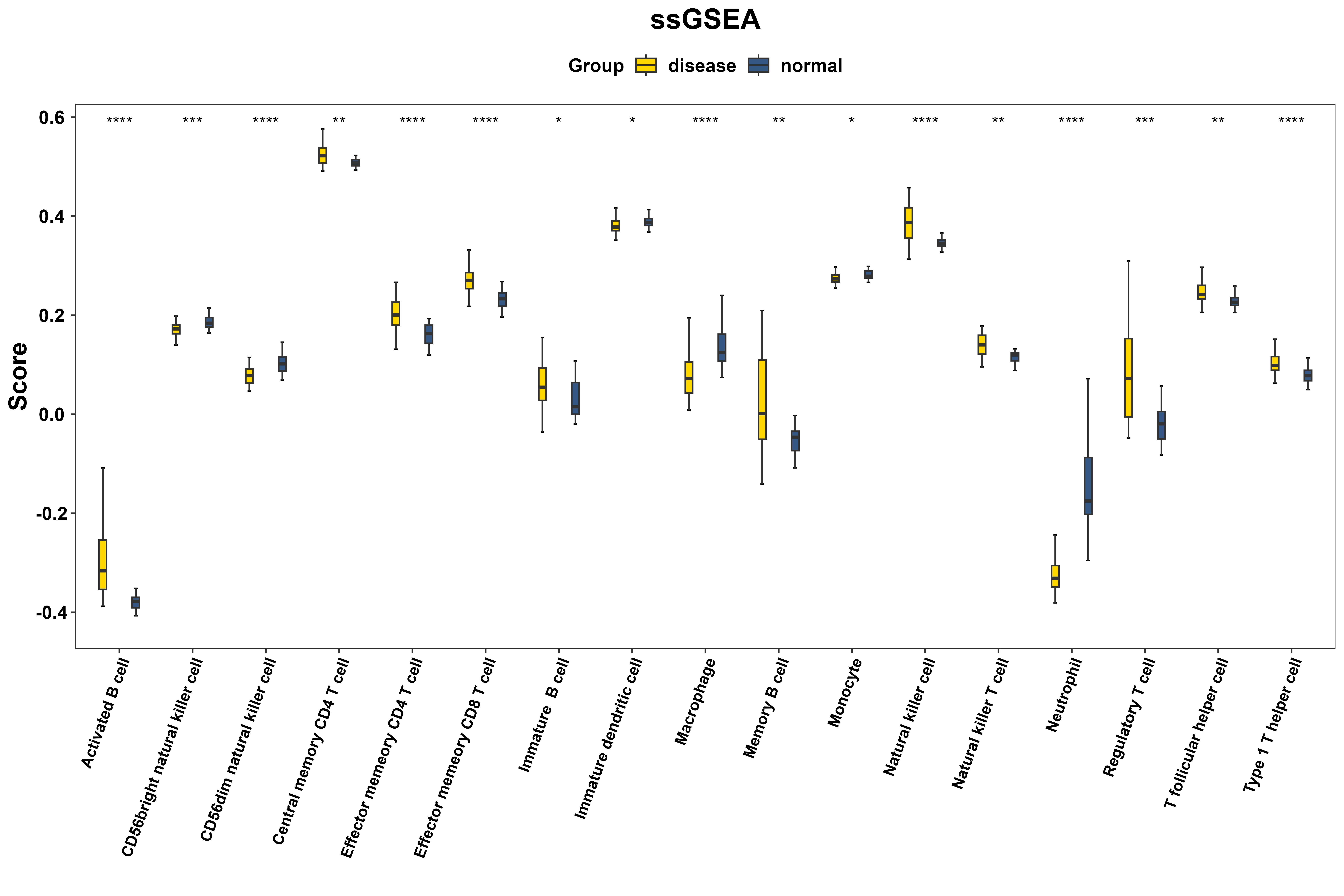

Supplement: Supplemental Information 3 [file peerj-13-20346-s003.zip › supplementary file/11_immune_infiltration/ssGSEA/01.immue_cell_bar.png]

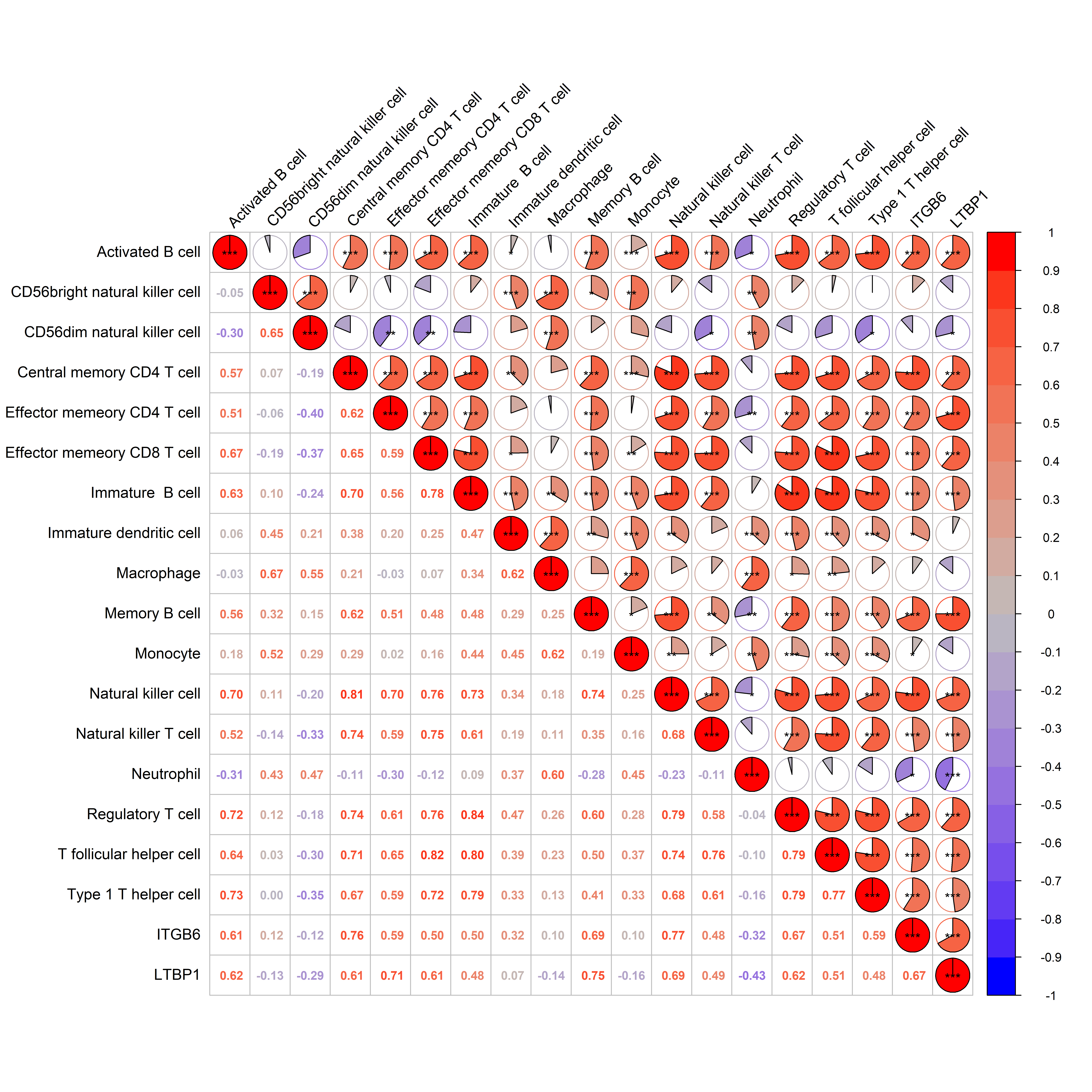

Supplement: Supplemental Information 3 [file peerj-13-20346-s003.zip › supplementary file/11_immune_infiltration/ssGSEA/03.cor_heatmap.png]

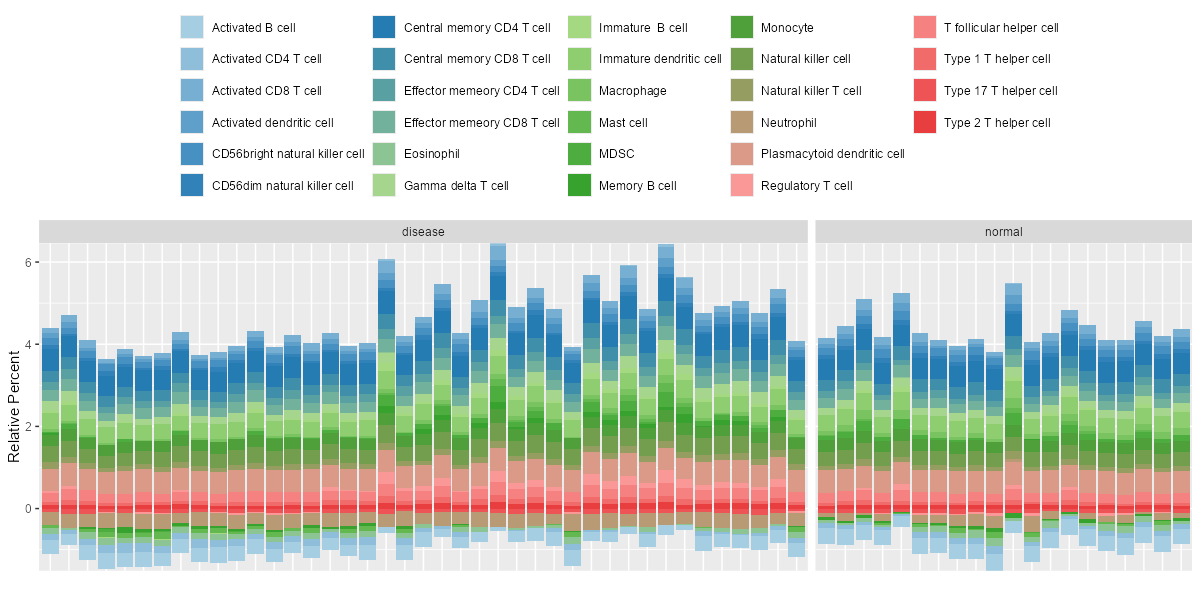

Supplement: Supplemental Information 3 [file peerj-13-20346-s003.zip › supplementary file/11_immune_infiltration/ssGSEA/02.immue_cell_map.png]

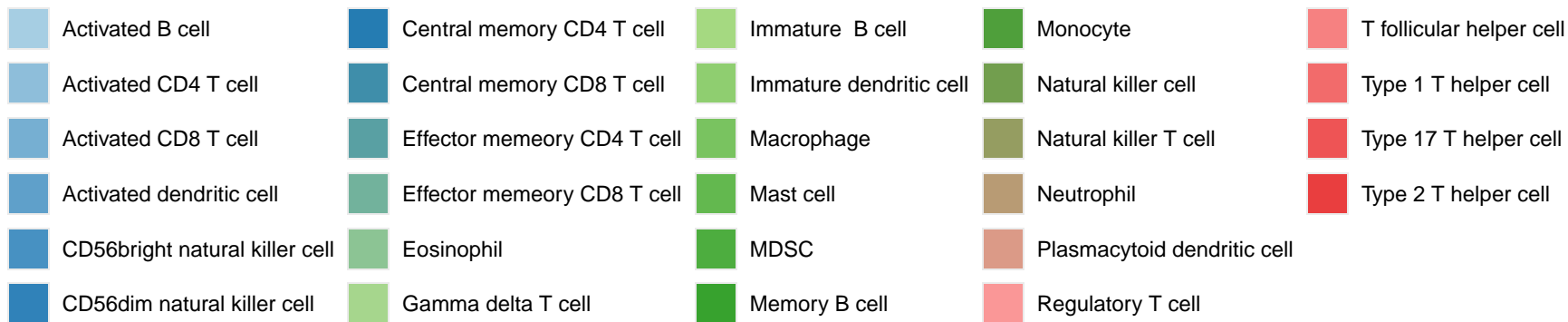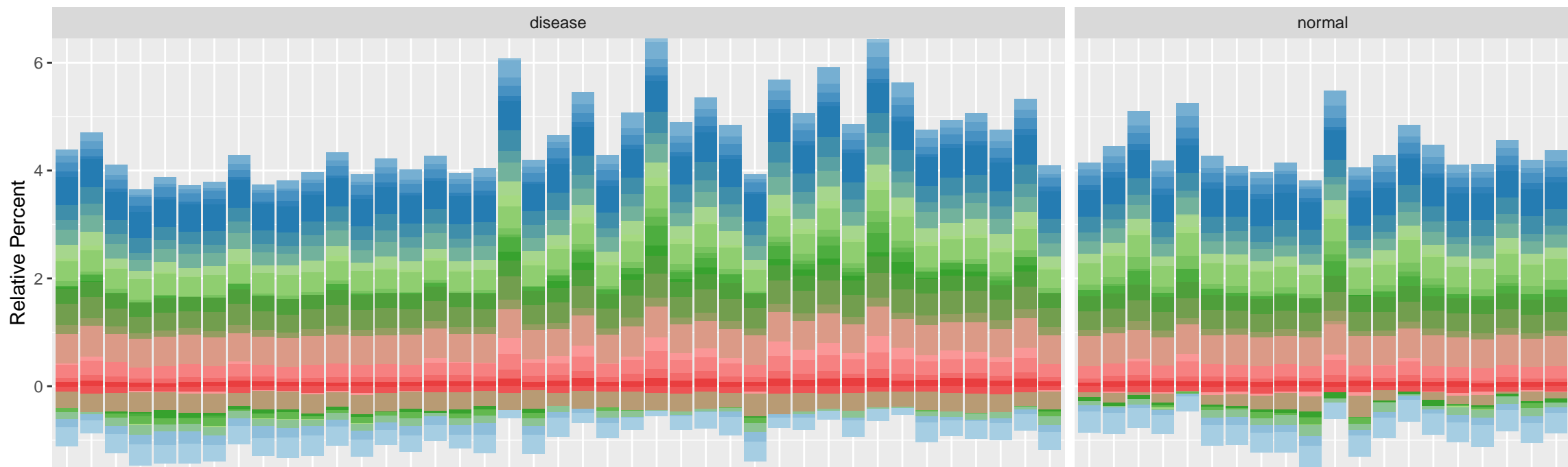

Supplement: Supplemental Information 3 [file peerj-13-20346-s003.zip › supplementary file/11_immune_infiltration/ssGSEA/02.immue_cell_map.pdf]

# Cibersort

Group ■ disease ■ normal

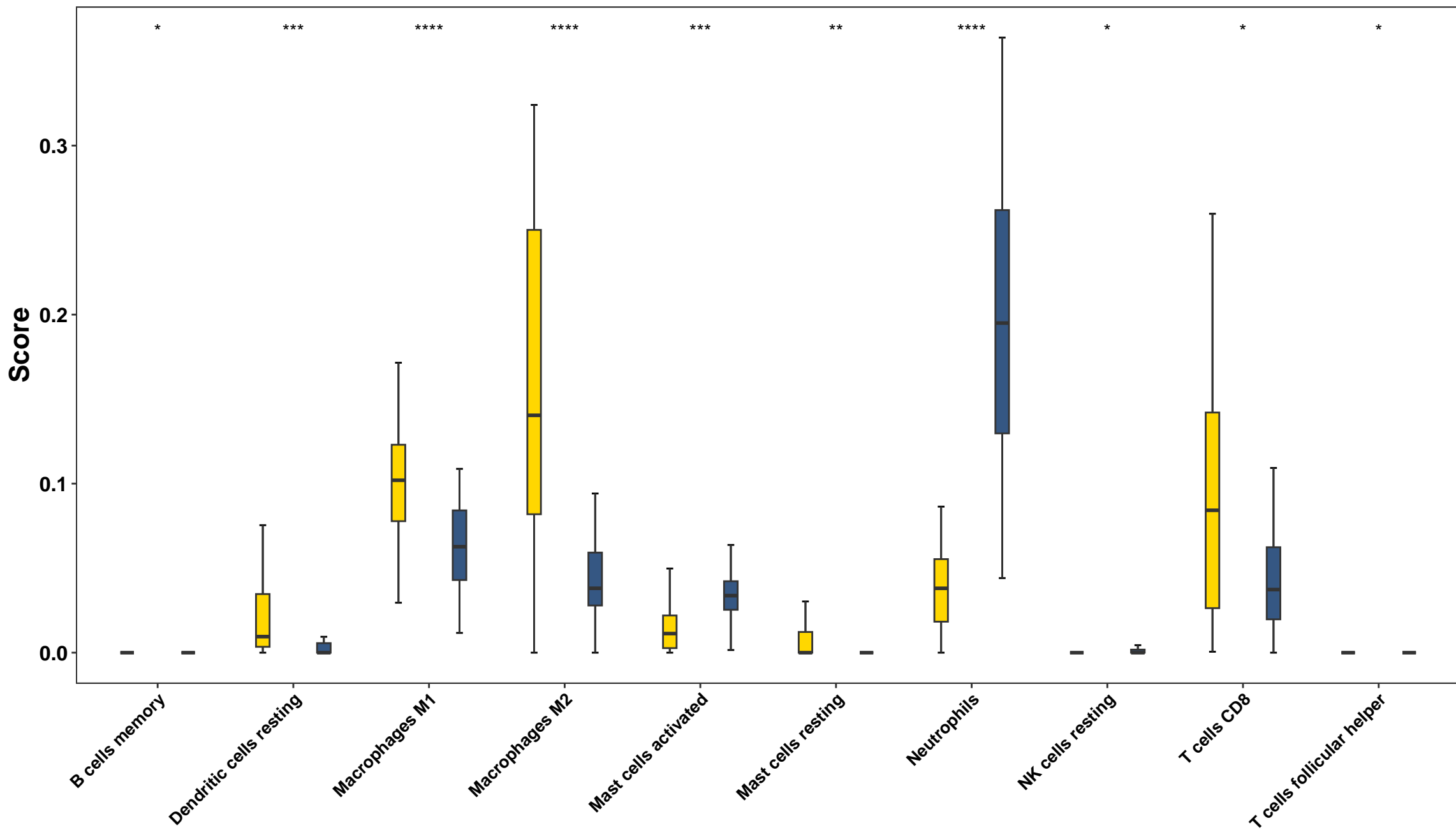

Supplement: Supplemental Information 3 [file peerj-13-20346-s003.zip › supplementary file/11_immune_infiltration/cibersort/01.immue_cell_bar.pdf]

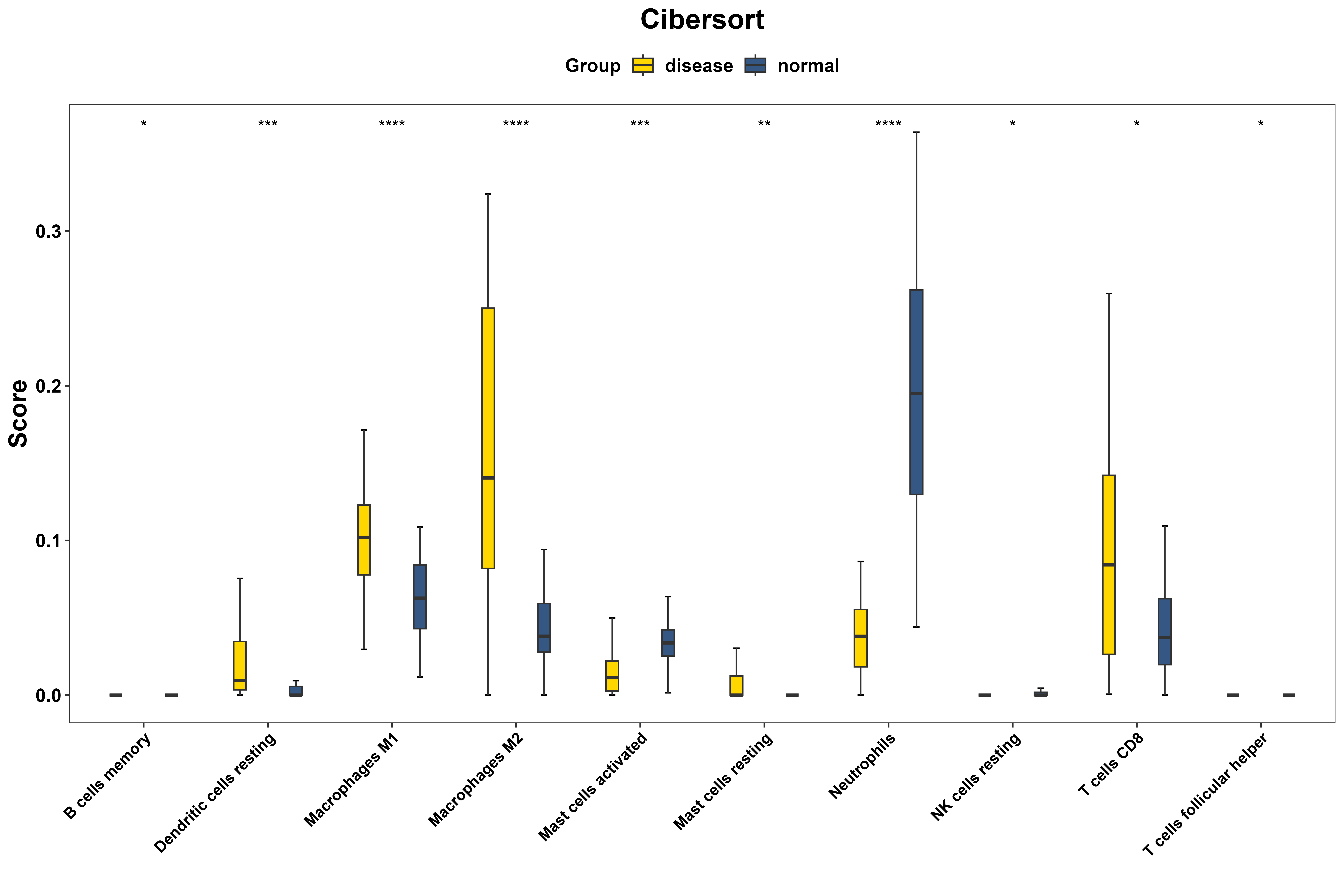

Supplement: Supplemental Information 3 [file peerj-13-20346-s003.zip › supplementary file/11_immune_infiltration/cibersort/01.immue_cell_bar.png]

# LTBP1

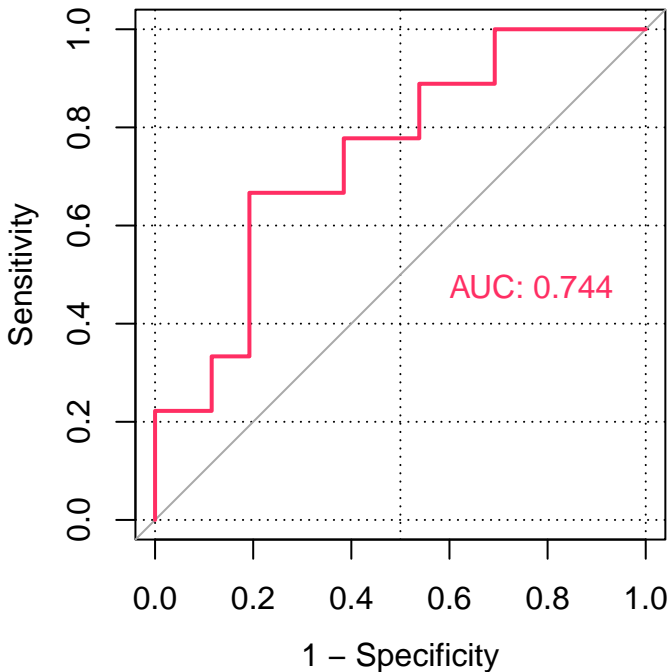

Supplement: Supplemental Information 3 [file peerj-13-20346-s003.zip › supplementary file/07_ROC/GSE30122/02.LTBP1.pdf]

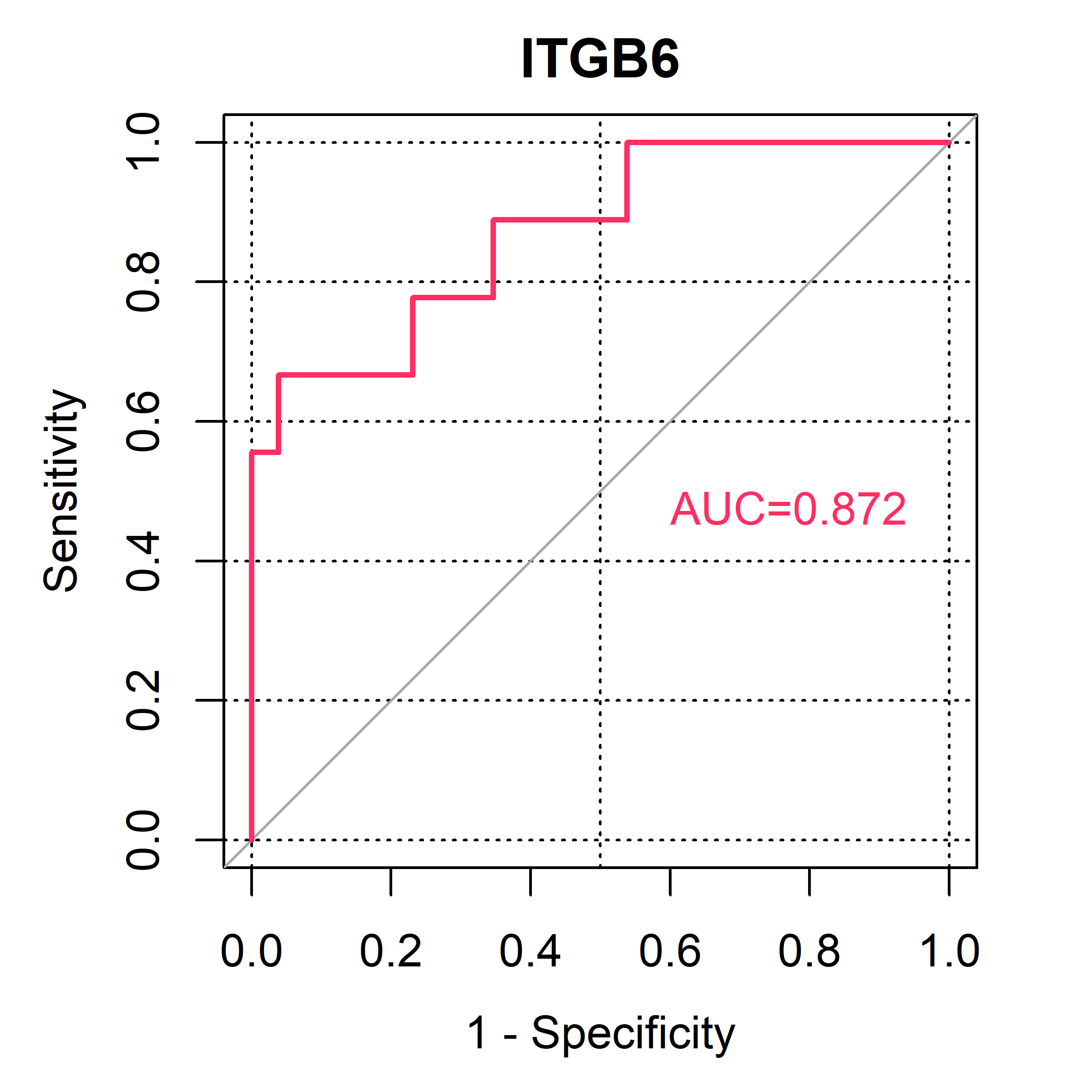

Supplement: Supplemental Information 3 [file peerj-13-20346-s003.zip › supplementary file/07_ROC/GSE30122/01.ITGB6.png]

# ITGB6

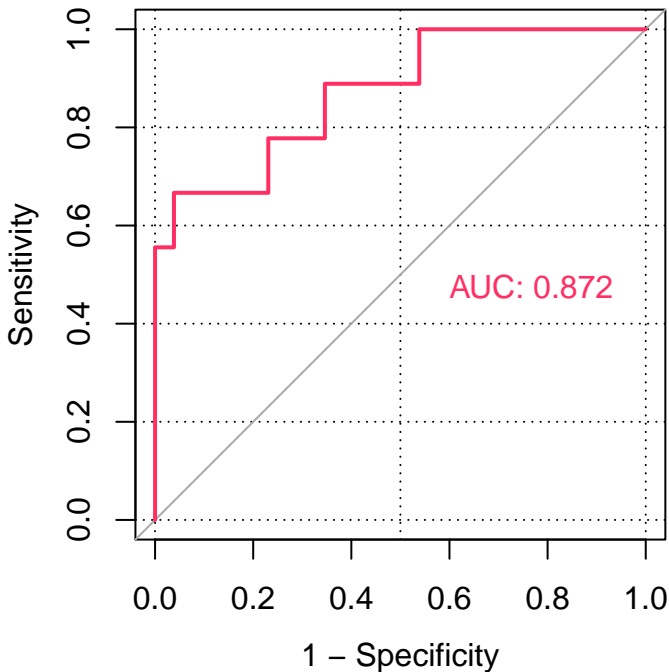

Supplement: Supplemental Information 3 [file peerj-13-20346-s003.zip › supplementary file/07_ROC/GSE30122/01.ITGB6.pdf]

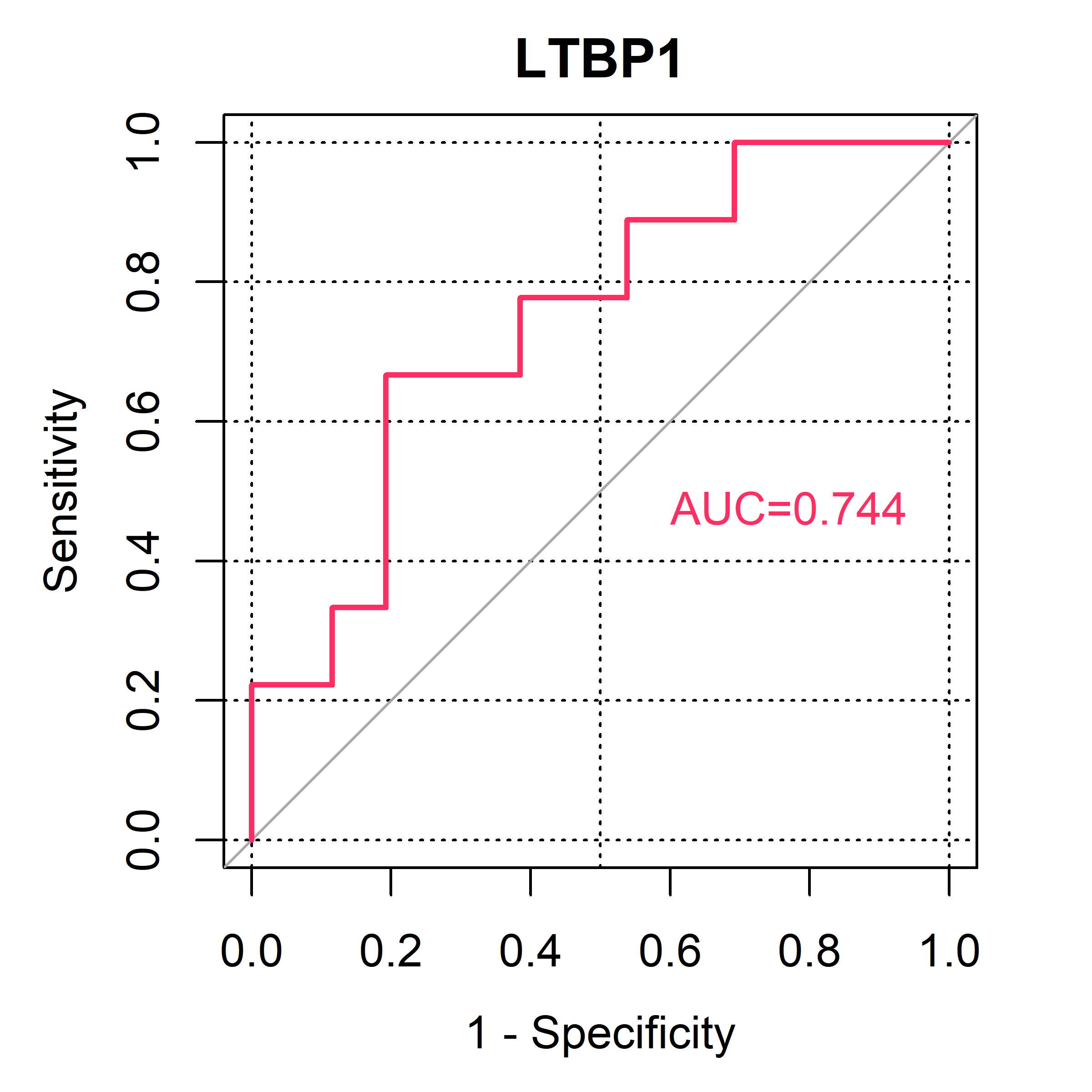

Supplement: Supplemental Information 3 [file peerj-13-20346-s003.zip › supplementary file/07_ROC/GSE30122/02.LTBP1.png]

# LTBP1

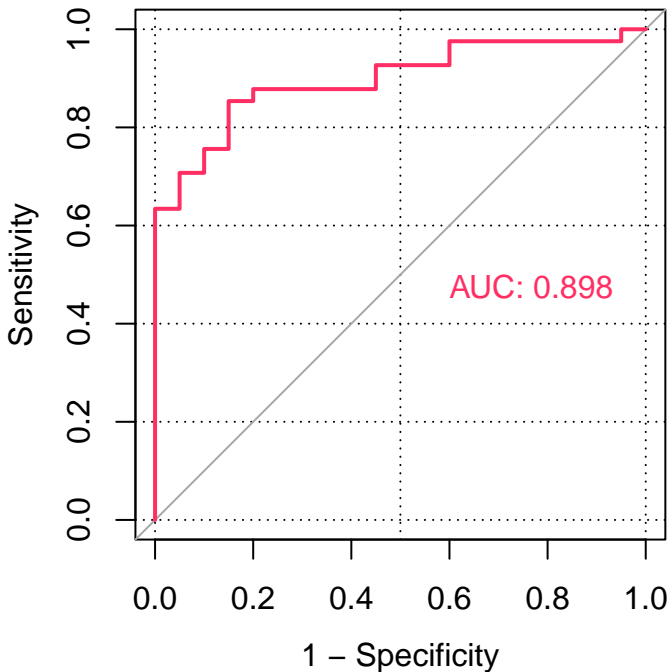

Supplement: Supplemental Information 3 [file peerj-13-20346-s003.zip › supplementary file/07_ROC/GSE96804/02.LTBP1.pdf]

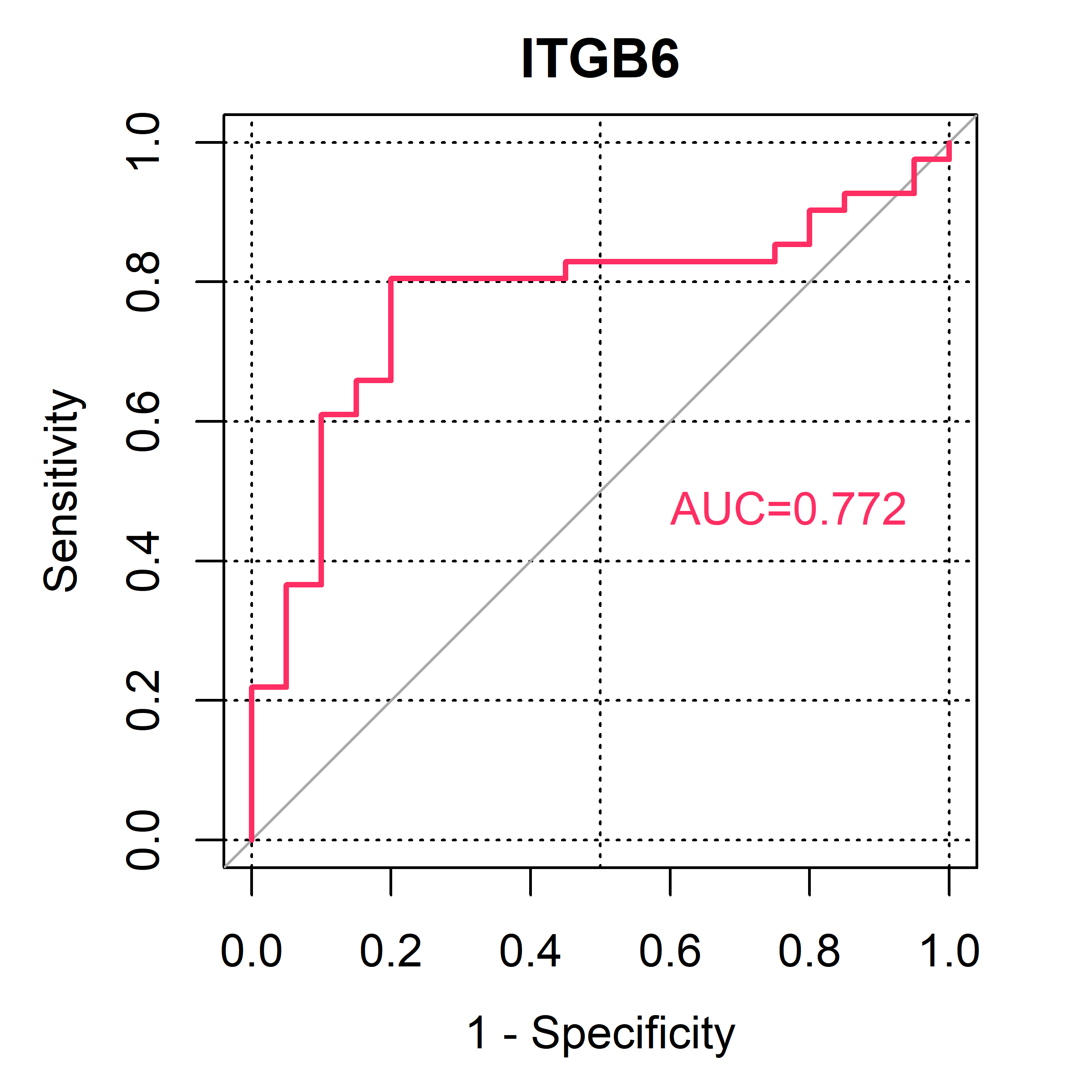

Supplement: Supplemental Information 3 [file peerj-13-20346-s003.zip › supplementary file/07_ROC/GSE96804/01.ITGB6.png]

# ITGB6

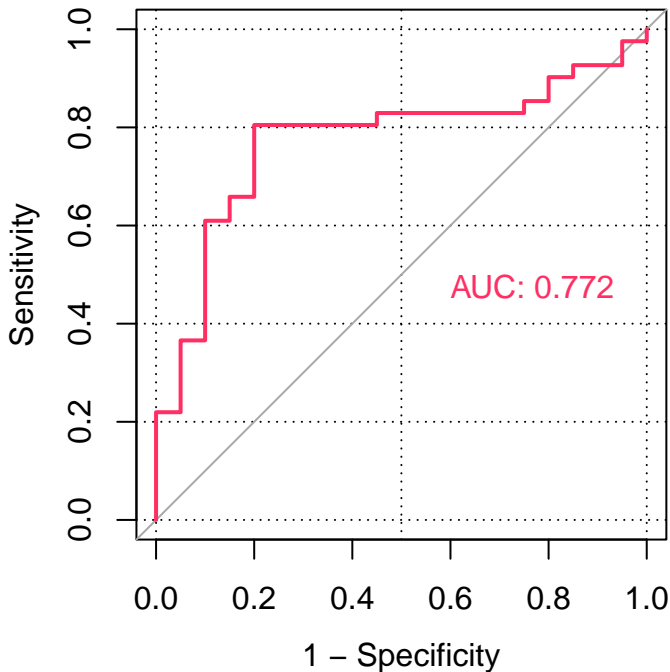

Supplement: Supplemental Information 3 [file peerj-13-20346-s003.zip › supplementary file/07_ROC/GSE96804/01.ITGB6.pdf]

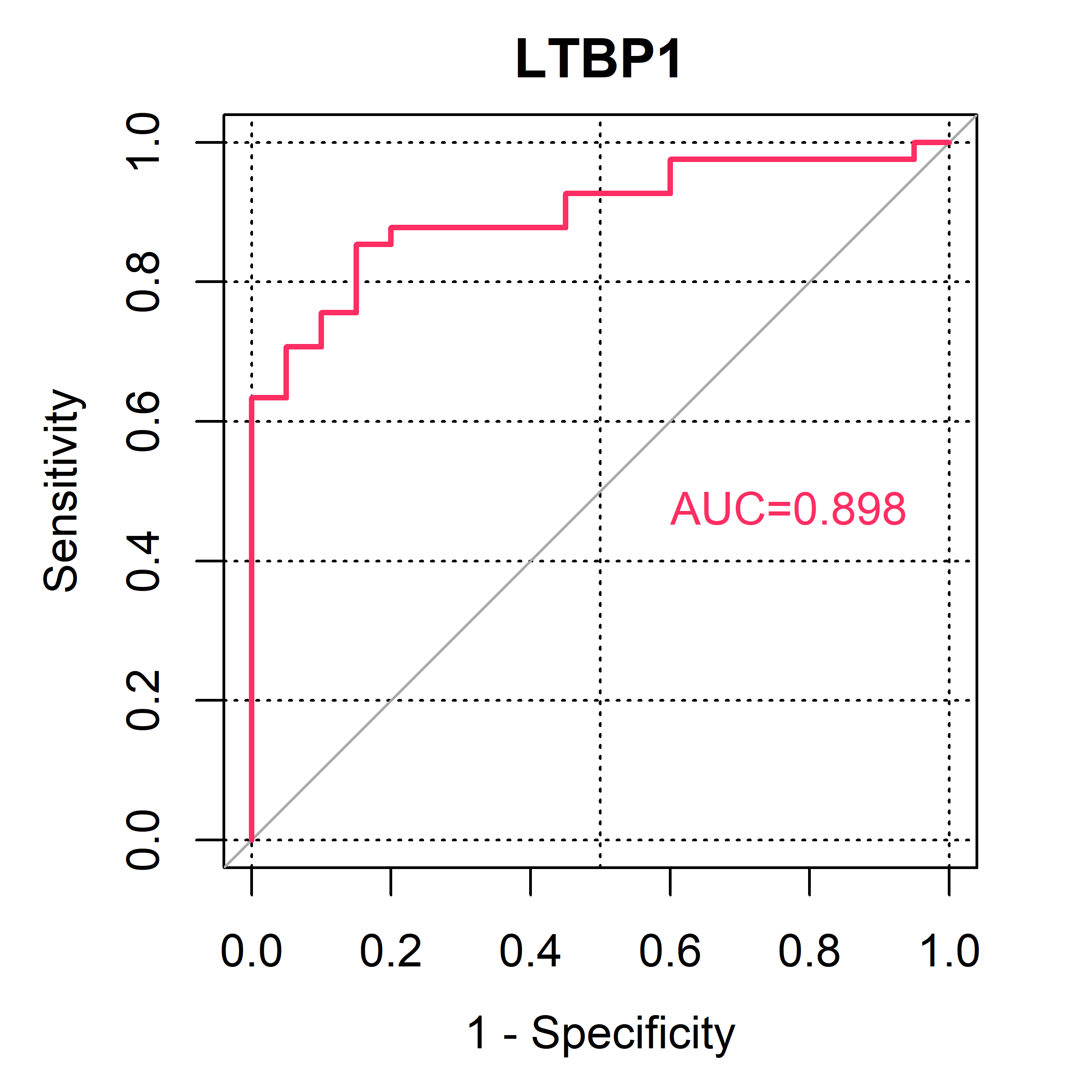

Supplement: Supplemental Information 3 [file peerj-13-20346-s003.zip › supplementary file/07_ROC/GSE96804/02.LTBP1.png]

Train\_Expression of hub\_gene

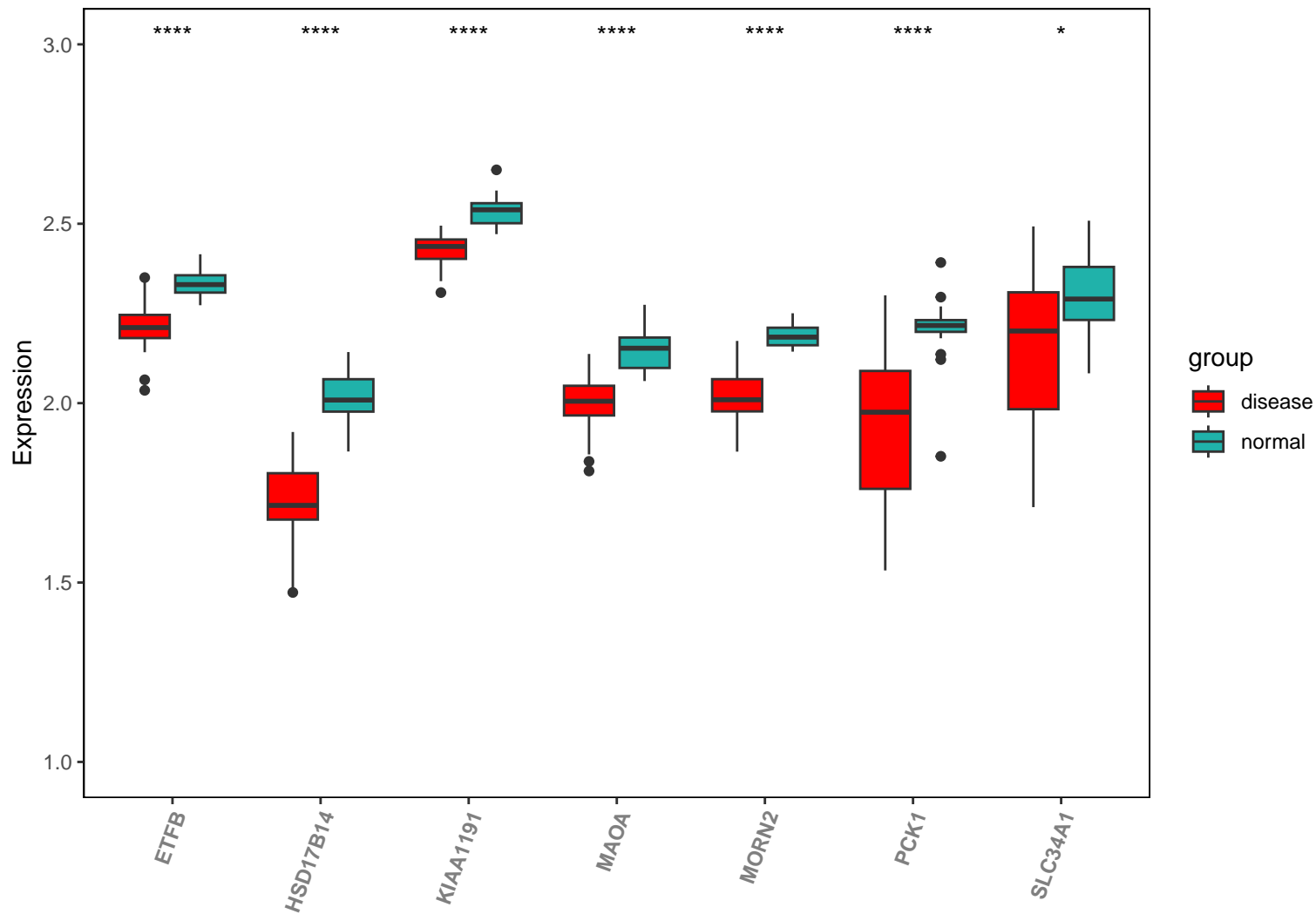

Supplement: Supplemental Information 3 [file peerj-13-20346-s003.zip › supplementary file/06_expression/brown/01.brown_hub_gene_Expression_train.pdf]

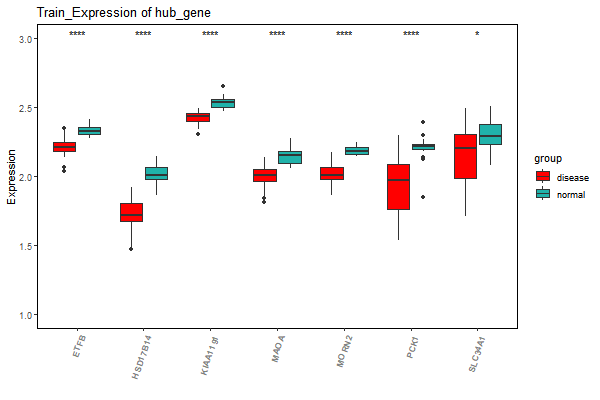

Supplement: Supplemental Information 3 [file peerj-13-20346-s003.zip › supplementary file/06_expression/brown/01.brown_hub_gene_Expression_train.png]

test\_Expression of hub\_gene

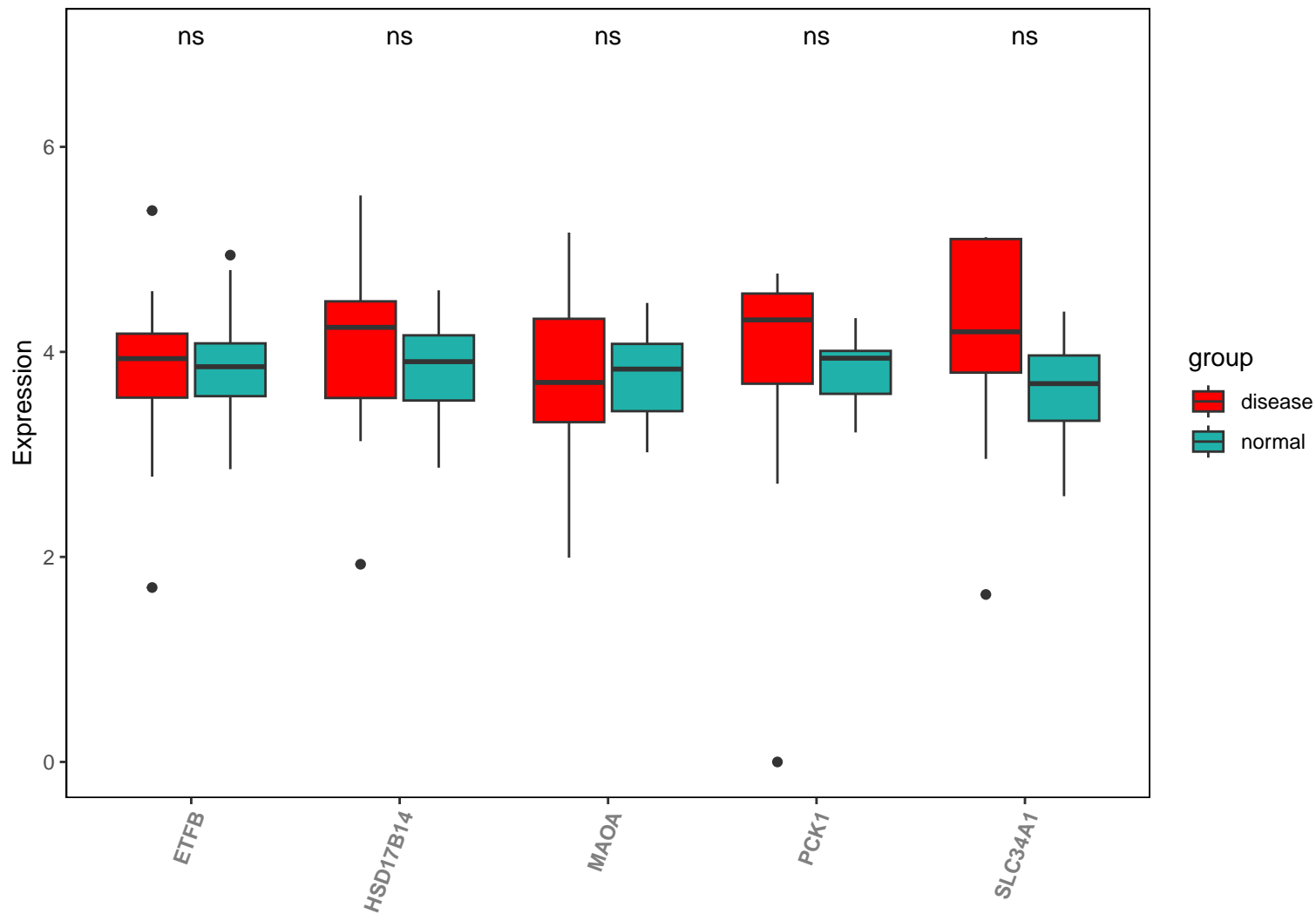

Supplement: Supplemental Information 3 [file peerj-13-20346-s003.zip › supplementary file/06_expression/brown/02.brown_hub_gene_Expression_test.pdf]

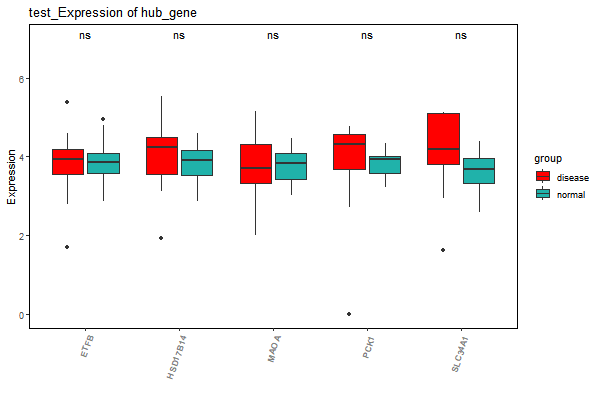

Supplement: Supplemental Information 3 [file peerj-13-20346-s003.zip › supplementary file/06_expression/brown/02.brown_hub_gene_Expression_test.png]

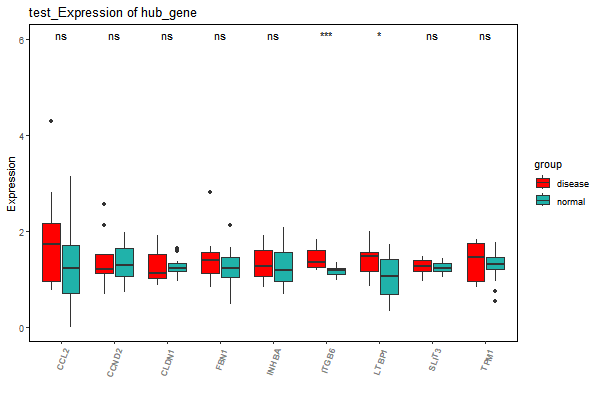

Supplement: Supplemental Information 3 [file peerj-13-20346-s003.zip › supplementary file/06_expression/blue/02.blue_hub_gene_Expression_test.png]

test\_Expression of hub\_gene

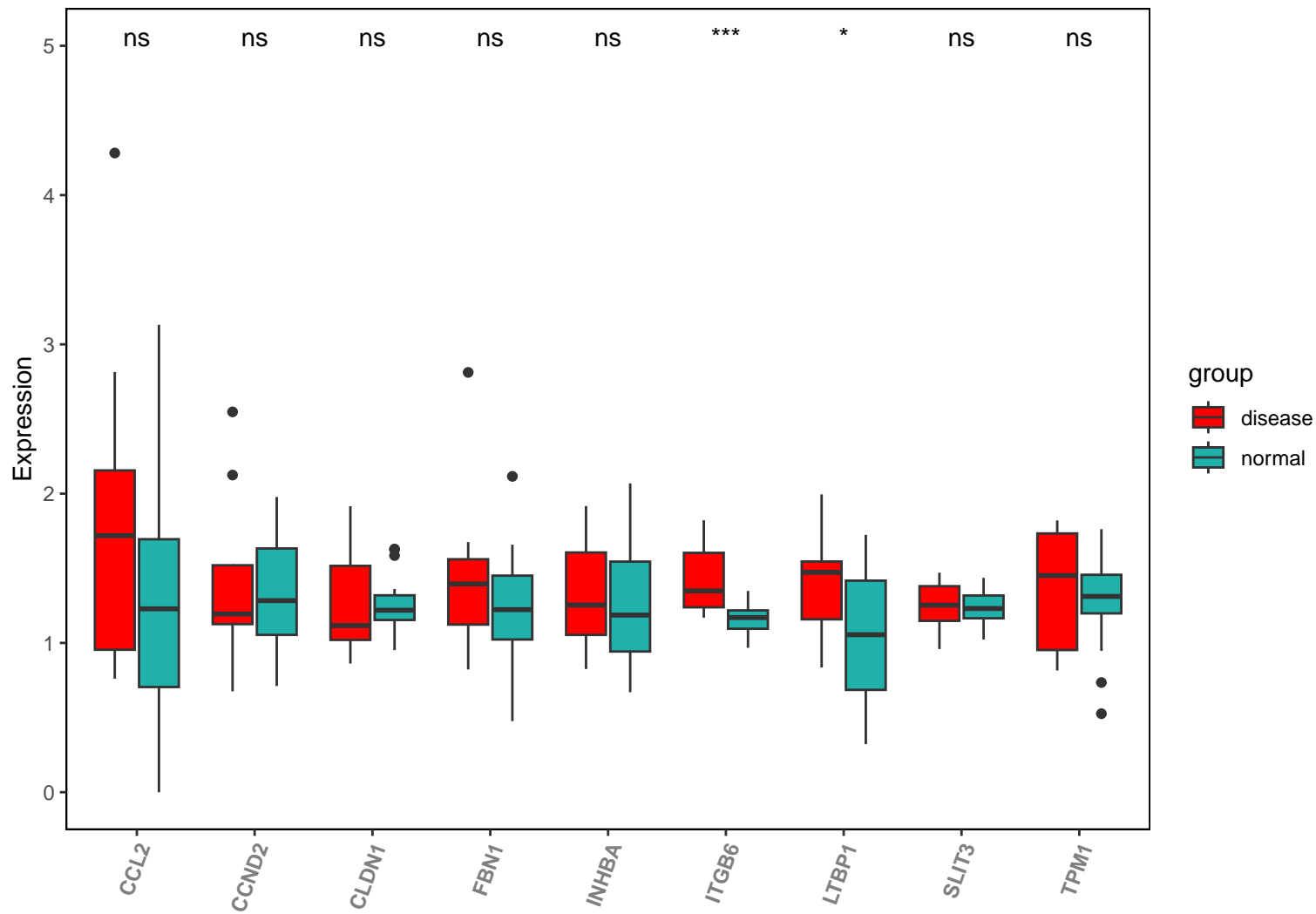

Supplement: Supplemental Information 3 [file peerj-13-20346-s003.zip › supplementary file/06_expression/blue/02.blue_hub_gene_Expression_test.pdf]

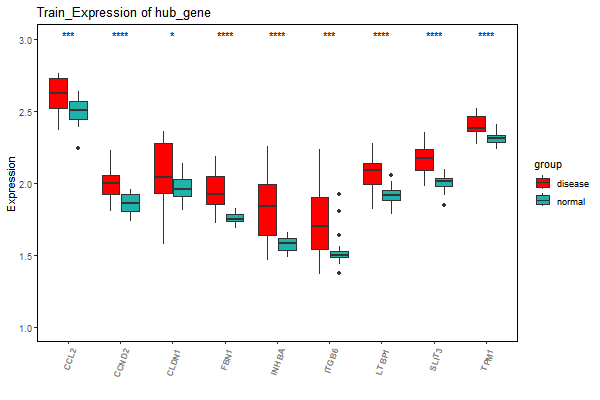

Supplement: Supplemental Information 3 [file peerj-13-20346-s003.zip › supplementary file/06_expression/blue/01.blue_hub_gene_Expression_train.png]

Train\_Expression of hub\_gene

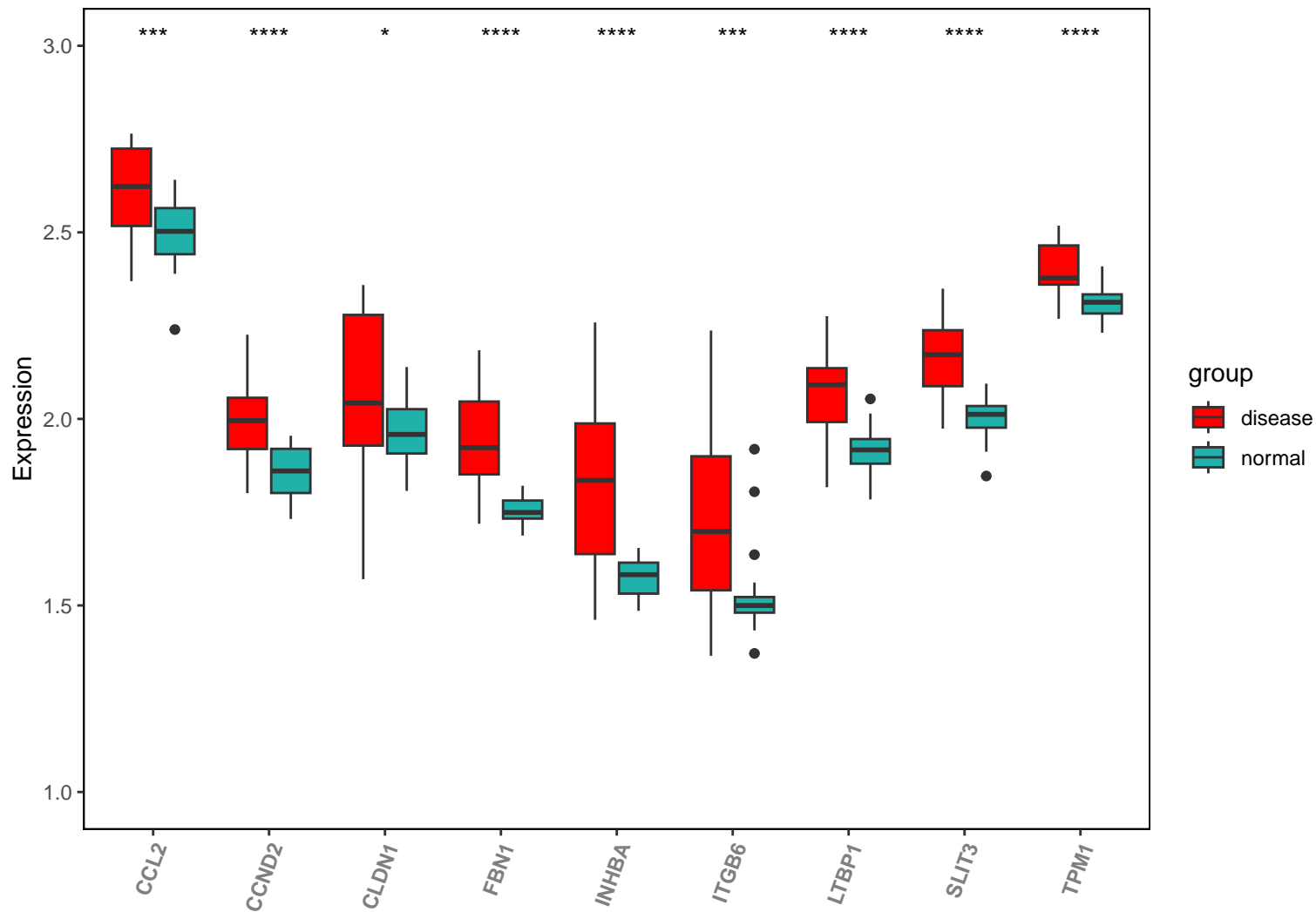

Supplement: Supplemental Information 3 [file peerj-13-20346-s003.zip › supplementary file/06_expression/blue/01.blue_hub_gene_Expression_train.pdf]

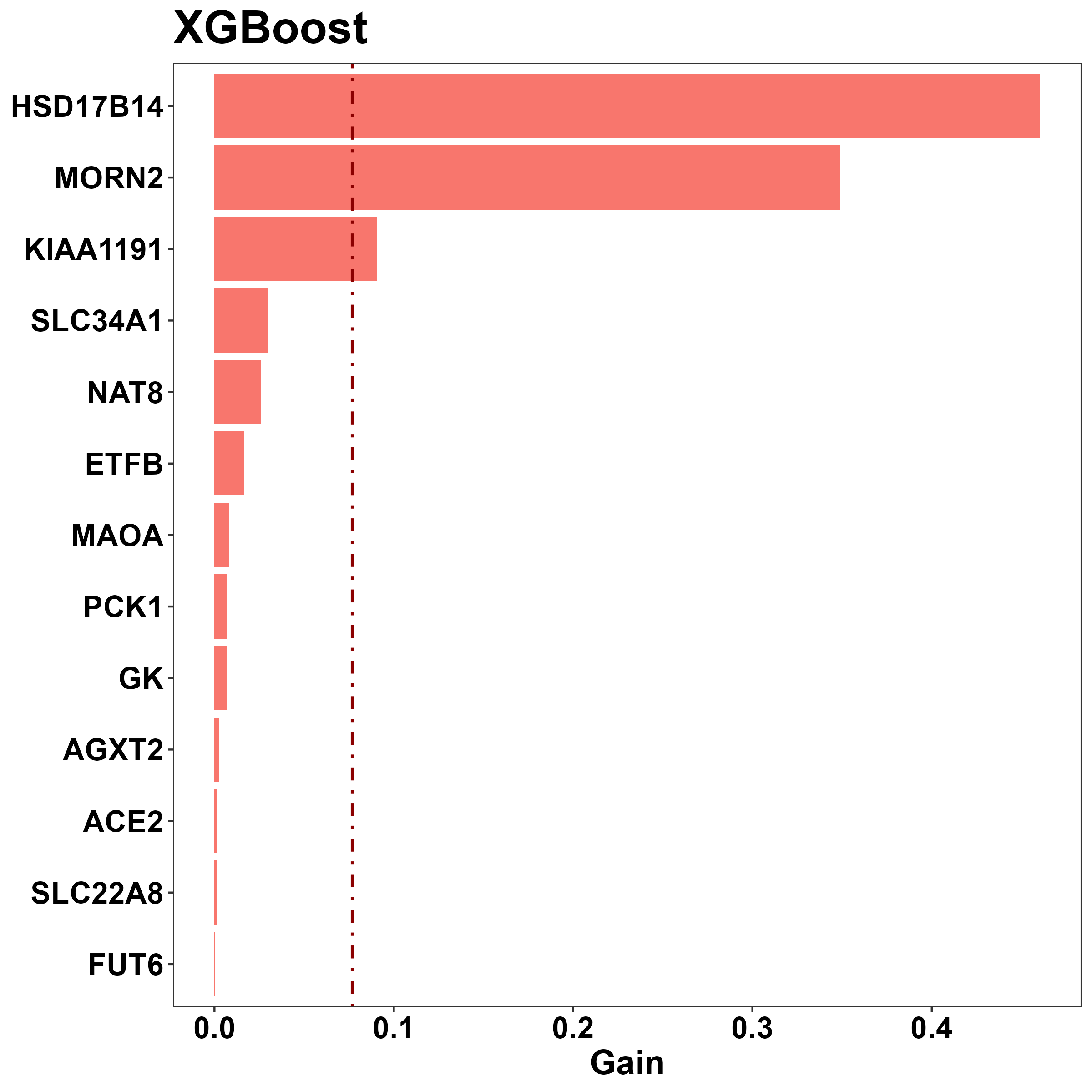

Supplement: Supplemental Information 3 [file peerj-13-20346-s003.zip › supplementary file/05_Machine_learning/xgboost/brown/brown_XGBoost_importance.png]

# XGBoost

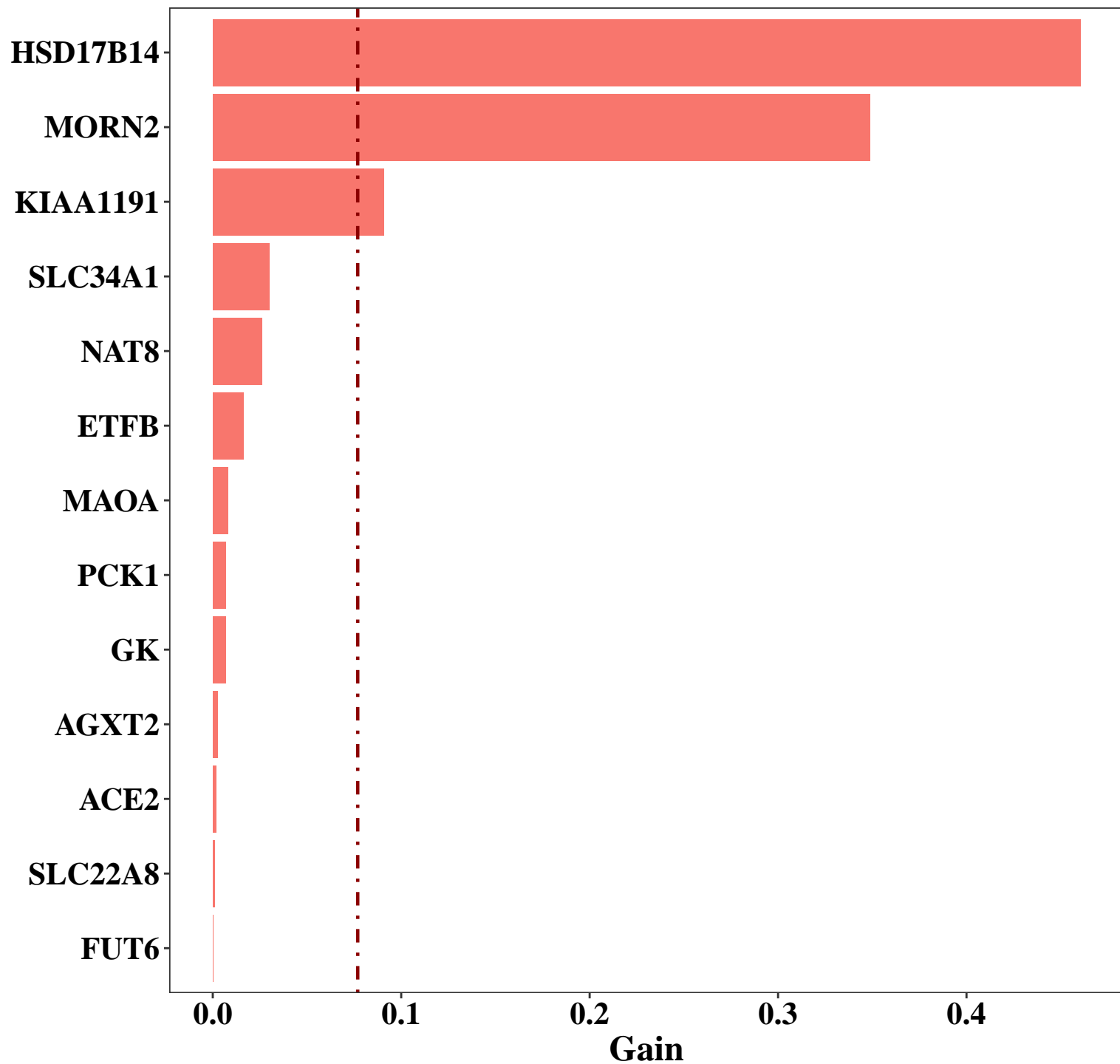

Supplement: Supplemental Information 3 [file peerj-13-20346-s003.zip › supplementary file/05_Machine_learning/xgboost/brown/brown_XGBoost_importance.pdf]

# XGBoost

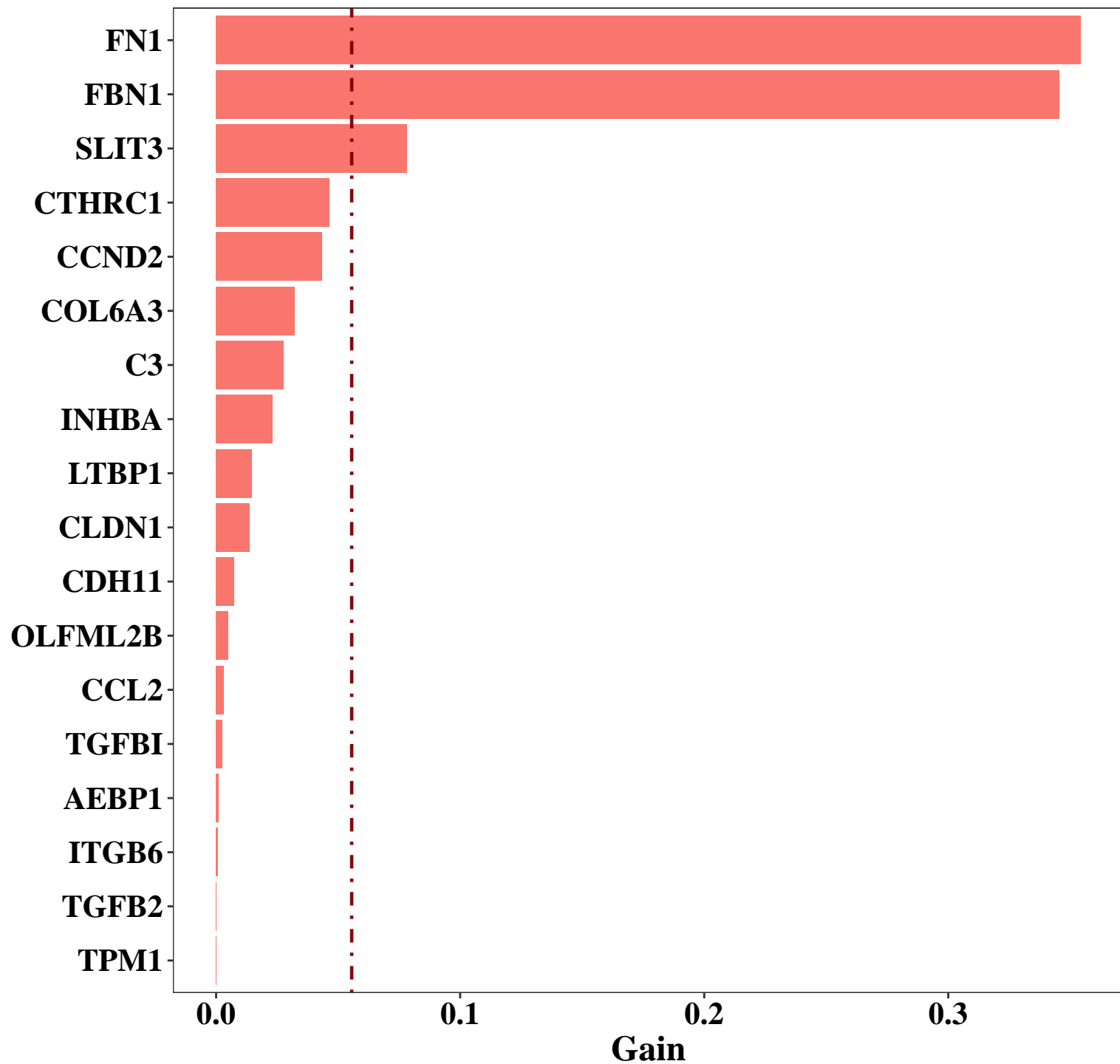

Supplement: Supplemental Information 3 [file peerj-13-20346-s003.zip › supplementary file/05_Machine_learning/xgboost/blue/blue_XGBoost_importance.pdf]

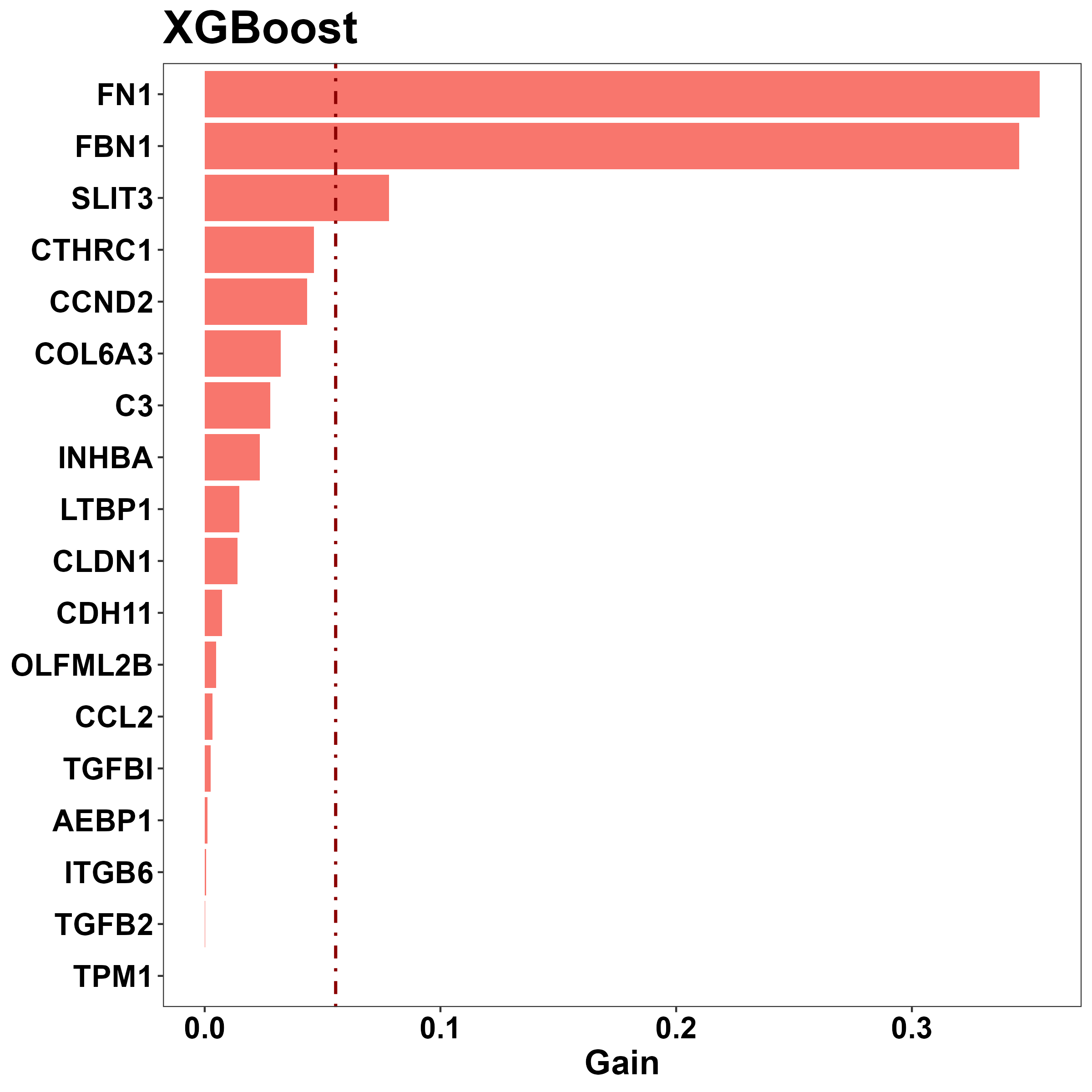

Supplement: Supplemental Information 3 [file peerj-13-20346-s003.zip › supplementary file/05_Machine_learning/xgboost/blue/blue_XGBoost_importance.png]

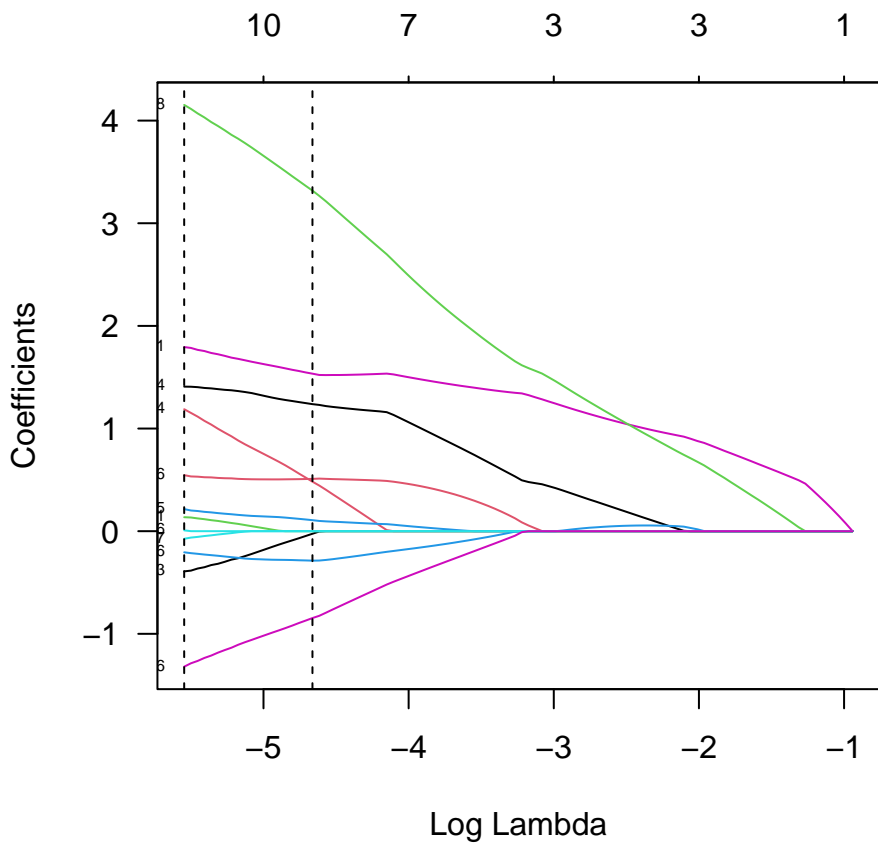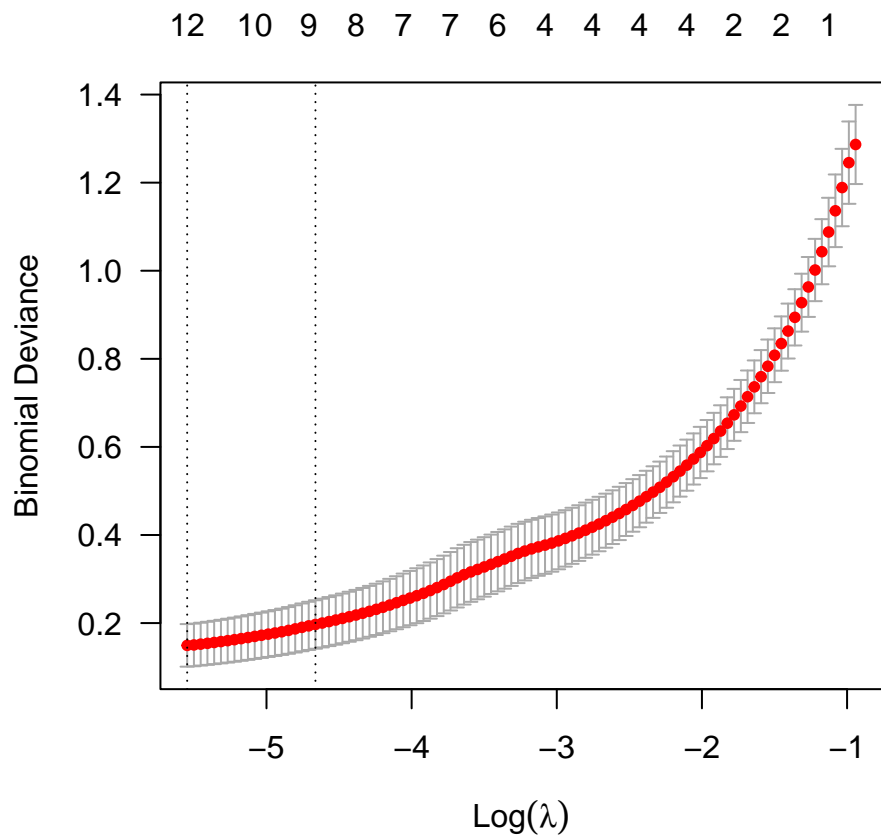

Supplement: Supplemental Information 3 [file peerj-13-20346-s003.zip › supplementary file/05_Machine_learning/lasso/brown/01.brown_lasso_model.pdf]

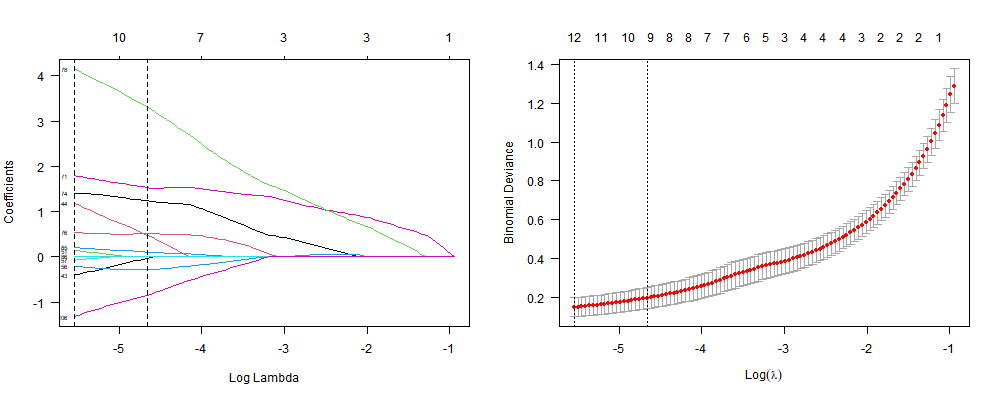

Supplement: Supplemental Information 3 [file peerj-13-20346-s003.zip › supplementary file/05_Machine_learning/lasso/brown/01.brown_lasso_model.png]

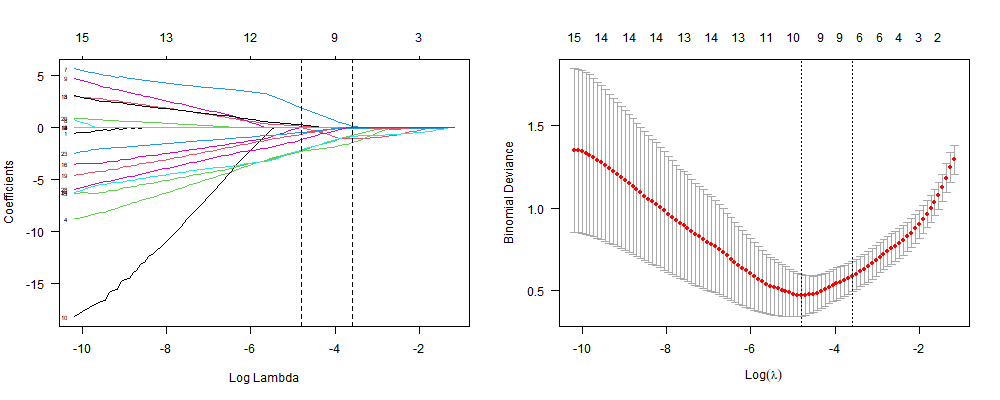

Supplement: Supplemental Information 3 [file peerj-13-20346-s003.zip › supplementary file/05_Machine_learning/lasso/blue/01.blue_lasso_model.png]

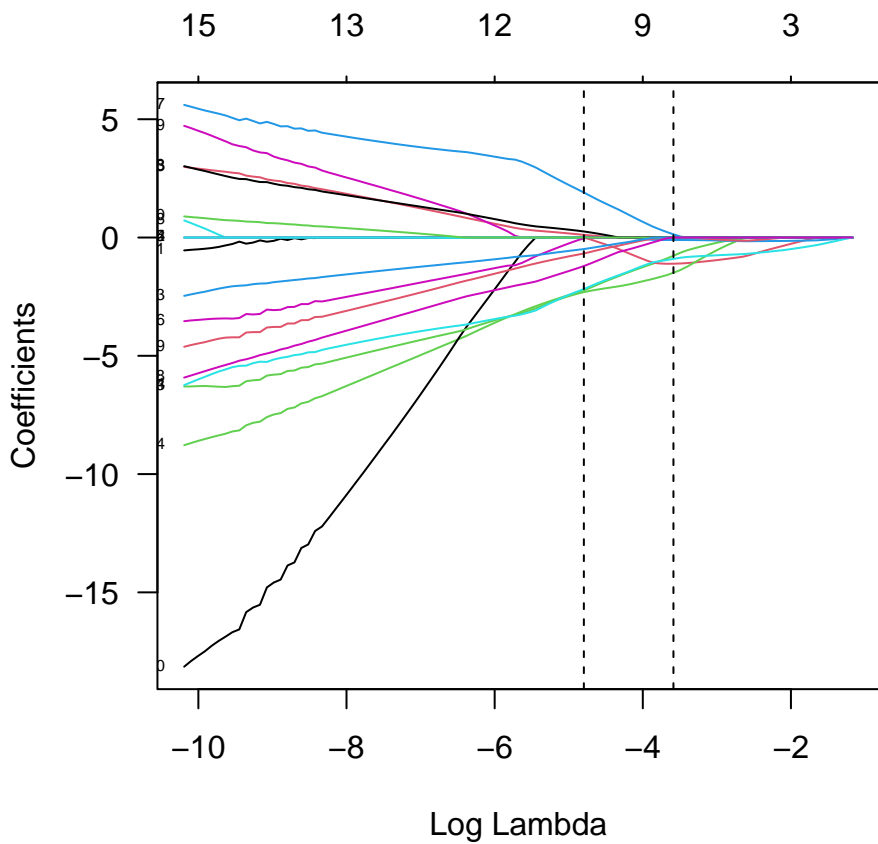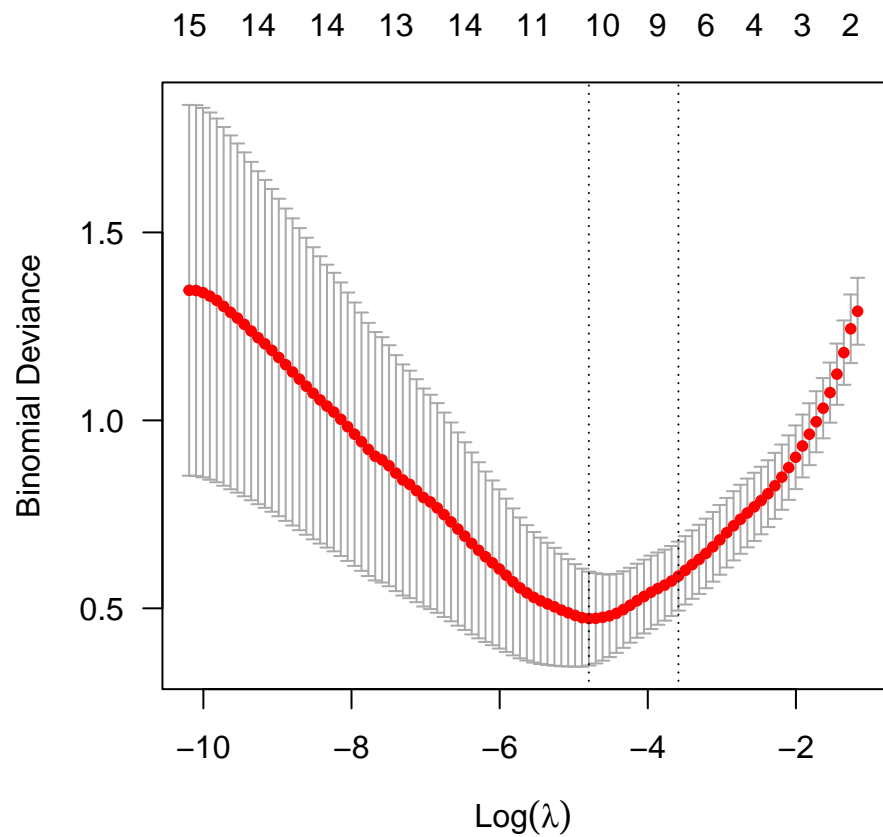

Supplement: Supplemental Information 3 [file peerj-13-20346-s003.zip › supplementary file/05_Machine_learning/lasso/blue/01.blue_lasso_model.pdf]
