# Supplementary material for: The cuproptosis-related gene ITGB6 and LTBP1 may be associated with diabetic kidney disease progression and immune cell infiltration
Source: PeerJ. 2025 Nov 11;13:e20346. doi: 10.7717/peerj.20346 (PMC12617370; doi:10.7717/peerj.20346)
Supplement: Supplemental Information 3 [file peerj-13-20346-s003.zip › supplementary file/13_classification/classification.docx]

| **[clr](javascript:clearChecked(document.listForm);)** **[all](javascript:checkAll(document.listForm);)**  | | \|  \| \| --- \|  \| **[Gene ID](javascript:listAction(document.listForm, '/list/list.do?filterLevel=1&sortField=GENE_ID&listType=1&sortOrder=1&trackingId=8B22855892E5D0F99E01BEA13F3240DD&species=All'))** \| \| --- \| | **Mapped IDs** | \|  \| \| --- \|  \| **[Gene Name](javascript:listAction(document.listForm, '/list/list.do?filterLevel=1&sortField=GENE_NAME&listType=1&sortOrder=1&trackingId=8B22855892E5D0F99E01BEA13F3240DD&species=All'))** \| \| --- \|  \| **[Gene Symbol](javascript:listAction(document.listForm, '/list/list.do?filterLevel=1&sortField=GENE_SYMBOL&listType=1&sortOrder=1&trackingId=8B22855892E5D0F99E01BEA13F3240DD&species=All'))** \| \| --- \|  \| **[Persistent id](javascript:listAction(document.listForm, '/list/list.do?filterLevel=1&sortField=PUBLIC_ID&listType=1&sortOrder=1&trackingId=8B22855892E5D0F99E01BEA13F3240DD&species=All'))** \| \| --- \|  \| **Orthologs** \| \| --- \| | \| [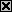](javascript:listAction(document.listForm,%20'/list/modifyPref.jsp?list_type_request=1%26col=GENE_BEST_FAM_SUBFAM_HIT%26url=/list/list.do?page=1%26filterLevel=1%26listType=1%26trackingId=8B22855892E5D0F99E01BEA13F3240DD')) \| \| --- \|  \| **[PANTHER Family/Subfamily](javascript:listAction(document.listForm, '/list/list.do?filterLevel=1&sortField=PANTHER_BEST_HIT&listType=1&sortOrder=1&trackingId=8B22855892E5D0F99E01BEA13F3240DD&species=All'))** \| \| --- \| | \| [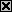](javascript:listAction(document.listForm,%20'/list/modifyPref.jsp?list_type_request=1%26col=GENE_PC_PROC%26url=/list/list.do?page=1%26filterLevel=1%26listType=1%26trackingId=8B22855892E5D0F99E01BEA13F3240DD')) \| \| --- \|  \| **[PANTHER Protein Class](javascript:listAction(document.listForm, '/list/list.do?filterLevel=1&sortField=PANTHER_PROTEIN_CLASS&listType=1&sortOrder=1&trackingId=8B22855892E5D0F99E01BEA13F3240DD&species=All'))** \| \| --- \| | \| [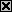](javascript:listAction(document.listForm,%20'/list/modifyPref.jsp?list_type_request=1%26col=GENE_SPECIES%26url=/list/list.do?page=1%26filterLevel=1%26listType=1%26trackingId=8B22855892E5D0F99E01BEA13F3240DD')) \| \| --- \|  \| **[Species](javascript:listAction(document.listForm, '/list/list.do?filterLevel=1&sortField=SPECIES&listType=1&sortOrder=1&trackingId=8B22855892E5D0F99E01BEA13F3240DD&species=All'))** \| \| --- \| |
| --- | --- | --- | --- | --- | --- | --- | --- | --- | --- | --- | --- | --- | --- | --- | --- | --- | --- | --- | --- | --- |
|  | **1.** | [HUMAN\|HGNC=6714\|UniProtKB=Q14766](https://www.pantherdb.org/genes/gene.do?acc=HUMAN\|HGNC=6714\|UniProtKB=Q14766) | LTBP1 | Latent-transforming growth factor beta-binding protein 1 [LTBP1](https://www.pantherdb.org/genes/gene.do?acc=HUMAN\|HGNC=6714\|UniProtKB=Q14766) [PTN002513800](https://www.pantherdb.org/node/node.jsp?id=PTN002513800) [orthologs](https://www.pantherdb.org/genes/gene.do?acc=HUMAN\|HGNC=6714\|UniProtKB=Q14766#orthologs) | [LATENT-TRANSFORMING GROWTH FACTOR BETA-BINDING PROTEIN 1 (PTHR24034:SF140)](https://www.pantherdb.org/panther/family.do?clsAccession=PTHR24034:SF140) | [extracellular matrix structural protein](https://www.pantherdb.org/panther/category.do?categoryAcc=PC00103) | Homo sapiens |
|  | **2.** | [HUMAN\|HGNC=6161\|UniProtKB=P18564](https://www.pantherdb.org/genes/gene.do?acc=HUMAN\|HGNC=6161\|UniProtKB=P18564) | ITGB6 | Integrin beta-6 [ITGB6](https://www.pantherdb.org/genes/gene.do?acc=HUMAN\|HGNC=6161\|UniProtKB=P18564) [PTN002467823](https://www.pantherdb.org/node/node.jsp?id=PTN002467823) [orthologs](https://www.pantherdb.org/genes/gene.do?acc=HUMAN\|HGNC=6161\|UniProtKB=P18564#orthologs) | [INTEGRIN BETA-6 (PTHR10082:SF11)](https://www.pantherdb.org/panther/family.do?clsAccession=PTHR10082:SF11) | [integrin](https://www.pantherdb.org/panther/category.do?categoryAcc=PC00126) | Homo sapiens |

Url： [http://www.pantherdb.org](http://www.pantherdb.org/)

[pantherdb.org/geneListAnalysis.do](https://www.pantherdb.org/geneListAnalysis.do)
