# Supplementary material for: The cuproptosis-related gene ITGB6 and LTBP1 may be associated with diabetic kidney disease progression and immune cell infiltration
Source: PeerJ. 2025 Nov 11;13:e20346. doi: 10.7717/peerj.20346 (PMC12617370; doi:10.7717/peerj.20346)
Supplement: Supplemental Information 3 [file peerj-13-20346-s003.zip › supplementary file/09_enrichment/GO/02.go_sig_vertical_bar.pdf]

capsulating s

xtracellular s

extracellula

reg

hreonine kin

en−containin

endopla

complex inv

lular matrix

stituent conf
