# Supplementary material for: The cuproptosis-related gene ITGB6 and LTBP1 may be associated with diabetic kidney disease progression and immune cell infiltration
Source: PeerJ. 2025 Nov 11;13:e20346. doi: 10.7717/peerj.20346 (PMC12617370; doi:10.7717/peerj.20346)
Supplement: Supplemental Information 3 [file peerj-13-20346-s003.zip › supplementary file/09_enrichment/GO/03.go_circle.pdf]

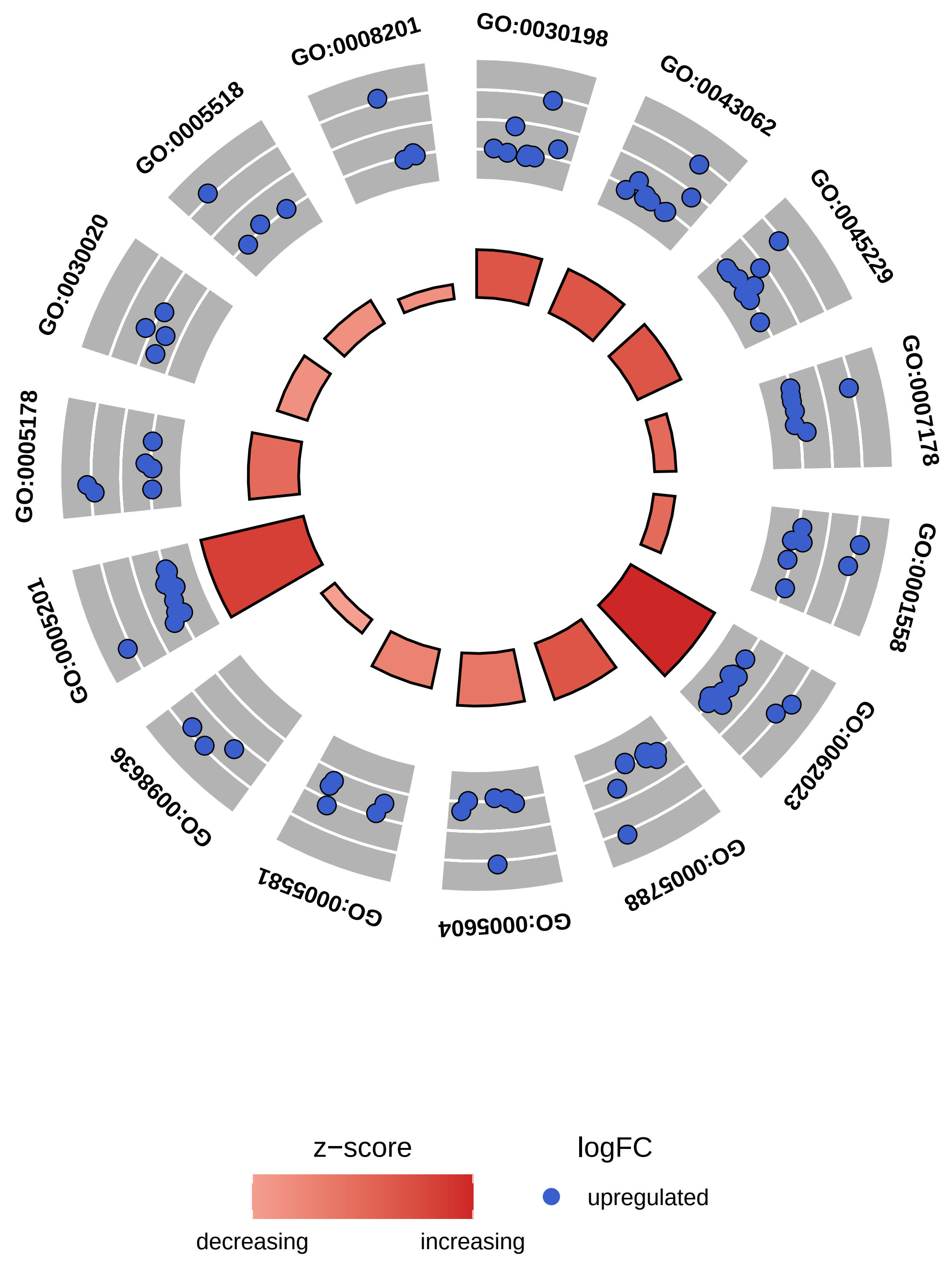

ID	Description
GO:0030198	extracellular matrix organization
GO:0043062	extracellular structure organization
GO:0045229	external encapsulating structure organization
GO:0007178	transmembrane receptor protein serine/threonine kinase signaling pathway
GO:0001558	regulation of cell growth
GO:0062023	collagen−containing extracellular matrix
GO:0005788	endoplasmic reticulum lumen
GO:0005604	basement membrane
GO:0005581	collagen trimer
GO:0098636	protein complex involved in cell adhesion
GO:0005201	extracellular matrix structural constituent
GO:0005178	integrin binding
GO:0030020	extracellular matrix structural constituent conferring tensile strength
GO:0005518	collagen binding
GO:0008201	heparin binding
